# Measuring disparate impact in human and machine decisions

**DOI:** 10.1073/pnas.2509765122

**Published:** 2026-07-20

**Authors:** Jongbin Jung, Sam Corbett-Davies, Johann D. Gaebler, Ravi Shroff, Sharad Goel

**Affiliations:** ^a^Medely, Los Angeles, CA 90401; ^b^ Stanford University, Stanford, CA 94305; ^c^ Department of Statistics, Harvard University, Cambridge, MA 02138; ^d^ Department of Applied Statistics, Social Science, and Humanities, New York University, New York, NY 10012; ^e^ Kennedy School of Government, Harvard University, Cambridge, MA 02138

**Keywords:** discrimination, risk-adjusted regression, included-variable bias, disparate impact

## Abstract

Empirical analyses have grown increasingly important in discrimination litigation with the greater availability of detailed data on individuals and decisions. A popular analytic strategy is to estimate disparities after adjusting for observed covariates, typically with a regression model, in hopes of ferreting out discriminatory intent. This approach, however, is ill-suited to auditing algorithms that are now commonly used to aid decisions, which typically do not include race or other legally protected factors as inputs. Motivated by legal understandings of disparate impact, we introduce an approach that aims to measure “unjustified” disparities in both human and machine decisions. Our method, which we call risk-adjusted regression, proceeds in three steps. In the first step, we combine all available information in a machine learning model to estimate the value, or inversely, the risk, of taking a certain action, such as approving a loan application or hiring a job candidate. Second, we measure disparities in decisions after adjusting for these risk estimates alone. Finally, in the third step, we assess the sensitivity of results to potential mismeasurement of risk. We demonstrate this approach on a detailed dataset of 2.2 million police stops of pedestrians in New York City, and show that traditional statistical tests of discrimination can substantially understate the magnitude of (risk-adjusted) racial disparities.

## Significance

Empirical studies of discrimination typically aim to estimate the effects of race and other legally protected characteristics on decisions. That goal, however, reflects an understanding of discrimination that fails to capture broader concerns of bias even when decisions are formally “race-blind.” Motivated by disparate impact law, we propose an alternative empirical conceptualization of discrimination that instead seeks to quantify “unjustified” disparities. For example, our approach would flag as discriminatory human or algorithmic hiring decisions advantaging members of one group over similarly qualified members of another, regardless of intent or whether group membership was explicitly considered. As facially neutral decision-making by humans and machines becomes more prevalent, our framework provides a practical, principled method for identifying legally or ethically problematic behavior.

There are two main legal doctrines of discrimination in the United States: disparate treatment and disparate impact. Disparate treatment protections bar government agents from acting with “discriminatory purpose” (1); disparate impact statutes, in contrast, bar policies that produce “unjustified” adverse effects on legally protected groups, even in the absence of animus (2). Among empirical researchers and practitioners, disparate treatment has, by far, received more attention. In the most common methodological approach, sometimes known as “kitchen sink” regression, analysts compare decision rates across race or other protected characteristics after adjusting for all available decision factors,^*^ typically using ordinary linear or logistic regression. For instance, in the lending context, one might compare loan officers’ lending rates to Black and White loan applicants after adjusting for income, credit score, and other relevant factors (3). This basic statistical strategy has been used in numerous studies to test for bias across domains including education (4, 5), employment (6), criminal justice (7–11), and medicine (12).

While analyses of disparate treatment are common in both the scientific literature and legal practice, the doctrine is ill-suited to assessing algorithmic decisions. In particular, algorithms are often formally “race blind,” and so cannot treat individuals from different groups differently in the legally relevant sense. For instance, it is rare for protected traits to be explicitly input into the models now common in lending (13), medicine (14), criminal justice (15), and other settings. Accordingly, attempts to audit these algorithms (e.g., with a standard regression analysis) will typically reveal, correctly, that race did not directly impact decisions.

Nonetheless, facially neutral algorithms can still adversely impact groups defined by race, gender, and other traits. For example, race- and gender-blind algorithms in healthcare and criminal justice have been found to be miscalibrated, producing risk estimates that are systematically skewed by group (16). In banking, the ways in which algorithmically generated loan offers are presented and marketed to online applicants can likewise create and exacerbate disparities. Finally, algorithms based on generative AI, which are increasingly used to guide employment decisions, might elevate vague understandings of “leadership” or “fit” in ways that disadvantage certain demographic groups—all without explicitly considering demographic traits (17).

Disparate impact, in contrast to disparate treatment, is concerned with the effects of a policy, not a decision maker’s intentions, and more naturally applies to the analysis of algorithmic decisions. Under the disparate impact standard, a practice may be deemed discriminatory if it has an unjustified adverse effect on protected groups, even in the absence of explicit categorization or animus. The disparate impact doctrine was formalized in the landmark U.S. Supreme Court case *Griggs v. Duke Power Co.* (1971).^†^ In 1955, the Duke Power Company instituted a policy that mandated employees have a high school diploma to be considered for promotion, which had the effect of drastically limiting the eligibility of Black employees. The Court found that this requirement had little relation to job performance, and thus deemed it to have an unjustified disparate impact.

Despite its importance, disparate impact analysis has received comparatively little attention in the empirical community (5, 19–23). Yet kitchen sink regression—the standard approach for assessing disparate treatment claims—is a poor fit for assessing whether practices are rationally justified, the relevant standard in disparate impact cases. Ayres (19) makes the point in the context of the original *Griggs* decision:

“One could imagine running a regression to test whether an employer was less likely to hire African American applicants than white applicants. It would be possible to control in this regression for whether the applicant had received a high-school diploma. Under the facts of *Griggs*, such a control would likely have reduced the racial disparity in the hiring rates. But including in the regression a variable controlling for applicants’ education would be inappropriate. The central point of *Griggs* was to determine whether the employer’s diploma requirement had a disparate racial impact. The possibility that including a diploma variable would reduce the estimated race effect in the regression would in no way be inconsistent with a theory that the employer’s diploma requirement disparately excluded African Americans from employment.”

In short, by including educational status in the regression, one would mask the policy’s unjustified disparate impact. (In *SI Appendix*, section 1.B, we give a simple formal illustration of this statistical phenomenon.)

Ayres (19) terms this phenomenon included-variable bias—“the worry that the statistical estimates of disparate impact are biased because the regression inappropriately includes nonrace variables” (20).^‡^ In *Griggs*, the Court found that education was unrelated to permissible employment aims, and so its inclusion in a regression analysis of disparate impact would have been inappropriate. But whether it is appropriate to control for a particular covariate is not always clear. In other employment contexts, education might be more strongly related to job performance. Similarly, given existing patterns of residential segregation, home ZIP codes tend to be highly correlated with race, weighing in favor of their exclusion when testing for disparate impact. But one could also argue that ZIP codes provide legitimate information relevant to a decision, and so excluding them would itself be problematic. Ayres (20) proposes a middle ground, suggesting that all covariates be included, but their coefficients capped to a “justifiable” level; in practice, however, it is difficult to determine and defend specific constraints on regression coefficients.^§^

Here, we present a statistically principled and logistically straightforward approach for measuring disparate impact in human and machine decisions. Our method, which we call risk-adjusted regression, proceeds in three steps. In the first step, we use all available information, including potential proxies of protected traits, to estimate the expected net benefit of taking a particular action—which we term “risk.”^¶^ For example, in the lending context, one might approximate net benefit by estimating the expected profitability of a loan, conditional on all available covariates; in hiring one could instead estimate an applicant’s expected productivity. Importantly, this step requires access to a rich set of covariates, which may not be available in all cases. In the second step, we assess disparities by regressing decisions (e.g., loan offers) on individual-level risk estimates and protected traits alone, allowing us to measure the extent to which similarly risky individuals receive different decisions. This strategy can be seen as formalizing Ayres’s (20) coefficient-capping procedure—with covariates used only to the extent that they are statistically justified by risk—and thus circumvents the problem of included-variable bias. Finally, we assess the sensitivity of results to potential mismeasurement of risk. In particular, we derive tight analytic bounds on risk-adjusted disparities as a function of how much risk estimates differ from true risk.

To demonstrate this approach, we examine 2.2 million stops of pedestrians conducted by the New York City Police Department between 2008 and 2011. After adjusting for a stopped individual’s statistical risk of carrying a weapon—based in part on detailed behavioral indicators recorded by officers—we find that stopped Black and Hispanic pedestrians are searched for weapons substantially more often than stopped White individuals. We find that these risk-adjusted disparities are considerably larger than disparities suggested by a standard regression that adjusts for all available covariates, underscoring the importance of accounting for included-variable bias. Finally, we show that our results are robust to potentially large errors in risk estimates.

## 1. Statistically Assessing Disparate Impact

Consider estimating risk-adjusted racial disparities in police searches of pedestrians for weapons—the application we discuss in more detail below. Let $A_{i}$ indicate whether the *i*-th stopped pedestrian was searched, $X_{i}$, the information available to the officer when deciding whether to conduct a search, $C_{i}$ the stopped individual’s race, and $W_{i}$ whether the individual is in possession of a weapon—regardless of the officer’s search decision.

Given this setup, we define ex ante risk to be

[1] $$\begin{array}{c} R=\;\mathrm{Pr}(W=1|X). \end{array}$$

In our policing example, $R_{i}$ is the probability that the *i*-th stopped individual, with covariates $X_{i}$, is carrying a weapon.^#^

Our goal is to quantify whether decisions systematically differ for individuals at the same level of risk. Many estimands summarizing such potential differences are possible, but, for simplicity, we consider the following nonparametric quantity:

[2] $$\begin{array}{c} E[\mathrm{Pr}(A=1|C=j,R)-\mathrm{Pr}(A=1|C=1,R)]. \end{array}$$

Eq. **2** defines risk-adjusted disparities to be the difference in the probability of taking an action for group C=j relative to the reference group C=1, after accounting for potential differences in risk across groups.^‖^

To facilitate computation—and, in particular, the sensitivity analysis presented below—we approximate the estimand in Eq. **2** by a linear probability model. Specifically, we estimate Pr(A=1∣C,R) as

[3] $$\begin{array}{c} \sum_{j=1}^{m}{\hat{\beta }}_{j}\cdot 1\left(C=j\right)+{\hat{\beta }}_{R}\cdot R, \end{array}$$

where 1(C=j) indicates membership in group *j* and ${\hat{\beta }}_{j}$ is its corresponding fitted coefficient. The difference between fitted coefficients,

[4] $$\begin{array}{c} d_{j}={\hat{\beta }}_{j}-{\hat{\beta }}_{1}, \end{array}$$

is our formal measure of disparate impact.

In our running example of police searches, if $d_{j}>0$—i.e., if ${\hat{\beta }}_{j}$ is greater than ${\hat{\beta }}_{1}$—it means that members of group *j* are searched more often than members of the reference group who were equally likely to be carrying a weapon. We would say that such elevated search rates are unjustified by risk. We note, however, that $d_{j}>0$ does not imply intentional discrimination. As in *Griggs*, unjustified disparate impact is possible even under a facially neutral policy undertaken without animus.

We have introduced risk-adjusted regression in the context of policing, but our general approach applies to both human and algorithmic decisions across a wide variety of settings. For example, we could similarly consider risk-adjusted disparities in hiring, where *W* represents a prospective employee’s productivity and *A* whether or not they were hired; and lending, where *W* represents the amount a prospective borrower will repay and *A* the bank’s lending decision. In contexts featuring a continuous rather than binary outcome measure *W*, such as these two examples, one would more generally define the expected net benefit *R* to be E[W∣X], the expected outcome given observed covariates *X*.

In our policing application—as well as in these other settings—empirically estimating risk-adjusted disparities $d_{j}$ is complicated by two factors. First, the information available to officers typically differs from that recorded in administrative datasets available to analysts. Second, analysts—and even officers—generally only know whether searched individuals were carrying weapons, but not whether unsearched individuals were carrying weapons. In other words, *W* is only observed when A=1. As a result, one cannot usually compute the risk *R* exactly, motivating our sensitivity analysis described below.

## 2. An Application to Policing

We apply our approach above to investigate the “stop-and-frisk” practices of the New York City Police Department (NYPD). (Reproduction materials for this analysis are available at https://github.com/jgaeb/rar-repro (44).) Police officers in the United States may stop and question pedestrians if they have “reasonable and articulable” suspicion of criminal activity; officers may additionally conduct a “frisk” (i.e., a brief pat-down of one’s outer garments) if they believe the stopped individual is carrying a weapon. Although a policy of stopping and frisking individuals is not inherently illegal, in *Floyd v. City of New York* (44), a federal district court ruled that the NYPD carried out such stops with racial animus, violating the Equal Protection Clause of the Fourteenth Amendment.

The court in *Floyd* was interested in assessing claims of disparate treatment; here, we reanalyze the data with a focus on disparate impact. We specifically consider frisk decisions, as they have a clear goal of ensuring officer safety by recovering weapons, and a well-measured outcome—whether a weapon was in fact found. We study 2.2 million pedestrian stops that occurred between 2008 and 2011. For each stop, we have detailed information on the date, time, and location of the stop; the demographics of the stopped individual (e.g., age, gender, and race); the suspected crime; the reasons prompting the stop (e.g., “furtive movements” or “suspicious bulge”); and additional circumstances surrounding the stop (e.g., evasive responses to questioning, witness reports, or evidence of criminal activity in the vicinity). (This information is recorded in a standardized way on UF-250 forms that officers were required to complete after each stop. A copy of the form can be found online.)

To start, we note that 1.7% of frisks turn up a weapon. White pedestrians are frisked in 44% of police stops, whereas Black and Hispanic pedestrians are frisked in 57% and 58% of stops, respectively, a 13 to 14 percentage point gap. These raw disparities are computed without adjusting for any covariates that might justify the observed differences between groups. At the other extreme is the “kitchen sink regression,” which adjusts for all observed prefrisk covariates in a standard linear probability model. In this case, stopped Black and Hispanic pedestrians are 3 to 4 percentage points more likely to be frisked, relative to White pedestrians with similar recorded characteristics. These kitchen-sink disparities are suggestive of disparate treatment (and similar evidence was indeed presented to the court in *Floyd* to support such an allegation), but they may understate the extent to which the policy imposes an unjustified disparate impact on racial minorities, due to included-variable bias.

### 2.1. Estimating Risk-Adjusted Disparities.

The key ingredient in applying risk-adjusted regression is estimating the risk R= Pr(W=1∣X), as in Eq. **1**, where *W* indicates whether a stopped individual has a weapon and *X* is the information available to the officer when making their frisk decision. As noted above, *X* and *W* are only partially and imperfectly observed, twin challenges that can be mitigated, but not eliminated. Our approach is thus to estimate risk as best we can, and then gauge the robustness of our results to estimation error. For ease of exposition, we adopt a simple estimation strategy, regressing weapon possession (*W*) on the covariates in the recorded data ($\tilde{X}$), fitting our model only on the subset of individuals who were, in reality, frisked. This yields estimates ${\hat{d}}_{j}$ of Eq. **4**, based on these estimated risks.^**^

We start by dividing the original stops into two subsets: 1) the approximately 1 million stops that occurred in 2008 and 2009, which we use to train a risk model; and 2) the remaining 1.2 million stops that occurred in 2010 and 2011, to which we apply the fitted risk model to estimate disparities in frisk decisions. In this way, we produce an estimate of ex ante risk ${\hat{R}}_{i}$ for every pedestrian stopped in the second subset of the data, including those who were not frisked. (See *Materials and Methods* below for further details.)

**Fig. 1.**
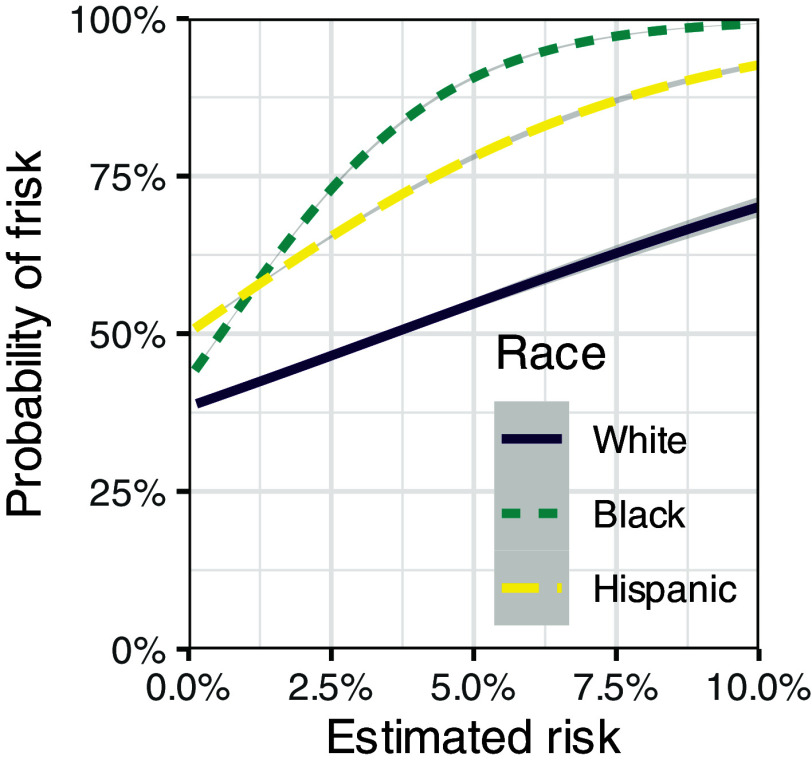
Frisk rates vs. risk, as estimated via logistic regression curves fit separately for each race group. Across risk levels, stopped Black and Hispanic pedestrians are frisked substantially more frequently than comparably risky White individuals, indicative of disparate impact.

Fig. 1 shows frisk rates as a function of estimated risk, disaggregated by race, also known as “risk-decision curves” (35).^††^ At every level of risk, stopped Black and Hispanic pedestrians are frisked at a much higher rate than stopped White individuals, a gap that is suggestive of disparate impact in frisk decisions.

**Fig. 2.**
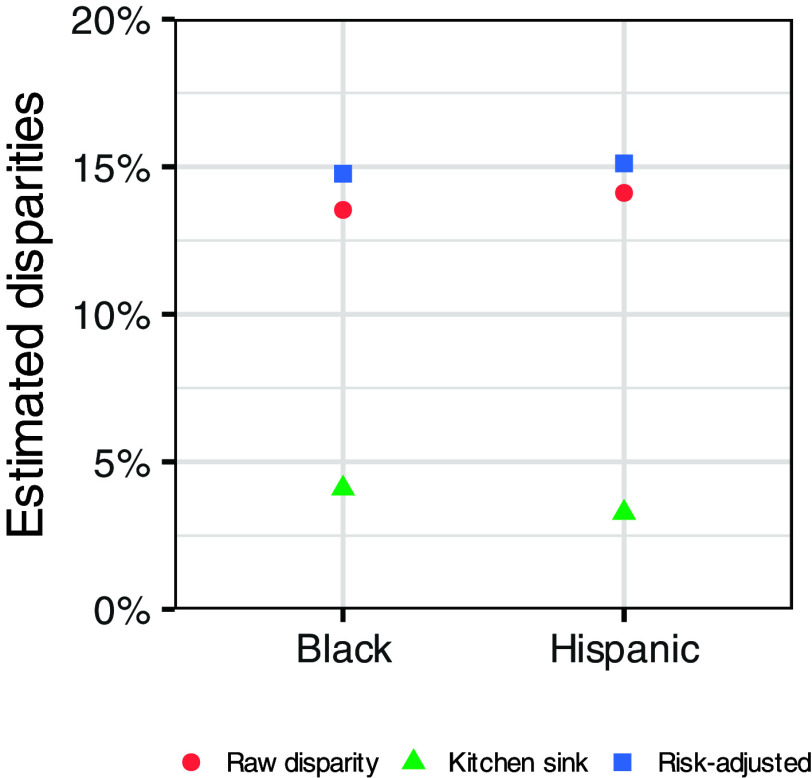
Racial gaps in frisk rates adjusting for different sets of covariates, where the *y*-axis shows the percentage point difference relative to stopped White individuals. The red dots show the raw disparities in frisk rates. As a measure of discrimination, raw disparities suffer from omitted-variable bias: there may, in theory, be legitimate reasons why Black and Hispanic pedestrians are more likely to be frisked. The green triangles show the estimated race effects in a kitchen-sink regression, adjusting for all prefrisk covariates. These estimates suffer from included-variable bias because they adjust for features that are correlated with race but unrelated to risk. The blue squares show the results of our risk-adjusted regression, adjusting exclusively for estimated risk of weapon possession. In all cases, estimated SE are less than 0.2 percentage points, and so are not visible in the plot.

To add quantitative detail to these qualitative results, we next compute risk-adjusted disparities, fitting the linear probability model in Eq. **3** on the second half of the NYPD data, computing estimates for the Black–White disparity ${\hat{d}}_{\text{Black}}$ and Hispanic-White disparity ${\hat{d}}_{\text{Hispanic}}$. Fig. 2 shows the results, together with the raw disparities and those estimated from a kitchen-sink model. We find that stopped Black and Hispanic pedestrians were about 15 percentage points more likely to be frisked than White pedestrians who were equally likely to be carrying a weapon. Further, the risk-adjusted disparities are in fact greater than the raw disparities in frisk rates. To understand why, we note that stopped White pedestrians were, on average, more likely to be carrying a weapon yet less likely to be frisked than racial minorities; as a result, the risk-adjusted gap in frisk rates is even larger than the raw, unadjusted gap. Finally, we see that the kitchen-sink regression dramatically underestimates the extent of disparate impact faced by minorities. In this case, the kitchen-sink model adjusts for a variety of features—including whether the stopped individual made “furtive movements”—that are correlated with race but are poor predictors of weapon possession, skewing estimates of disparate impact.^‡‡^

### 2.2. Sensitivity Analysis.

Computing risk-adjusted disparities depends critically on knowing individual-level risks. But we can only approximate these risks, both because officers observe factors that are predictive of risk but are not recorded in our data, and because outcomes (i.e., weapon possession) are only partially observed. It is thus important to gauge the sensitivity of our estimates of disparate impact to errors in our estimates of risk. We accordingly develop a method of sensitivity analysis that bounds the difference between $d_{j}$ and ${\hat{d}}_{j}$ when we constrain the true risks $R_{i}$ and estimated risks ${\hat{R}}_{i}$ to differ by some maximum amount, i.e., when

[5] $$\begin{array}{c} \frac{1}{n}\sum_{i=1}^{n}\left|{\hat{R}}_{i}-R_{i}\right|\leq \epsilon \end{array}$$

for some average absolute error *ϵ*.^§§^ The relationship between ${\hat{R}}_{i}-R_{i}$ and $d_{j}$ is nonconvex and involves a potentially large number of optimization variables, hindering computation. In *SI Appendix*, section 2, we derive an efficient algorithm for performing the sensitivity analysis, which we implement in the rar package for R, available on CRAN.

**Fig. 3.**
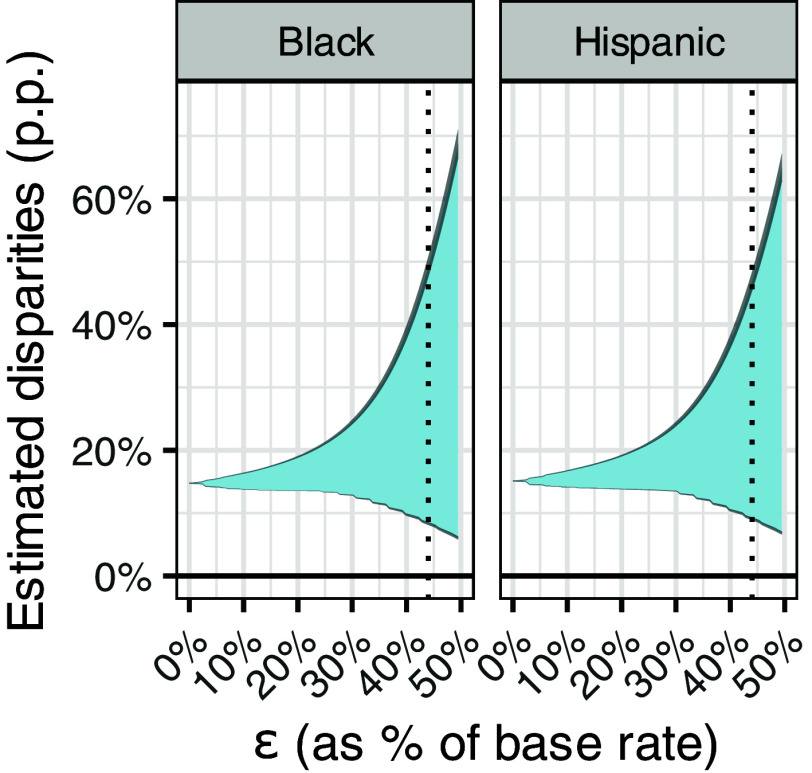
Sensitivity of the risk-adjusted disparities in frisk decisions to mismeasurement of risk. The blue bands bound our estimates of disparate impact as a function of the average absolute difference between true and estimated risks *ϵ*, relative to the base rate (1.7%). The dotted line at 44% (ϵ=0.7 p.p.) corresponds to a simulated situation with severe confounding. The small gray bands along the top and bottom of the blue bands represent 95% percentile bootstrapped CI (N=1,000; 48).

Fig. 3 displays our bounds on disparate impact as a function of the mismatch between true and estimated risk—operationalized in terms of *ϵ*, as defined in Eq. **5**. To ease interpretation, the horizontal axis in the plot is expressed in terms of the relative average absolute difference between true and estimated risks: *ϵ* divided by the overall weapon recovery rate among frisked individuals (1.7%). Our analysis shows that large risk-adjusted disparities remain even if we allow the true risk to differ considerably from our risk estimates. In particular, we would find large disparate impacts for both Black and Hispanic pedestrians even if the true risks differed from our risk estimates by 50% of the base rate.

It is impossible to know the precise extent to which our risk estimates differ from the true risk. To understand the plausible magnitude of the discrepancy, we conduct a simulation in which we remove a key set of risk-relevant covariates from the data, estimate risk based on the reduced information, and then measure differences between the original and new risk estimates. Specifically, we remove variables listed in two sections of the UF-250 stop forms that describe the “circumstances” prompting the encounter. These sections each consist of 10 binary variables—including, for example, “fits description,” “actions indicative of casing,” and “changing direction at sight of officer”—that are crucial for establishing the legal basis of the stop. We then compute the average absolute difference between our original risk estimates based on the full, uncensored data and the risk estimates based on the redacted data. We find that this value is 0.7 percentage points—or 44% of the base rate—which we take as an estimate of *ϵ* in a scenario with severe missing data. As shown in Fig. 3, this level of error (indicated by the dotted vertical lines) would yield an estimate of disparate impact that is, at a minimum, greater than 7.5 percentage points for both Black and Hispanic individuals. This sensitivity analysis suggests our results are robust to substantial mismeasurement of risk.

## 3. Discussion

Our primary policing application examined human decisions, but similar considerations apply to algorithms—especially those based on generative AI. Consider, for instance, hiring tools based on large language models (LLMs), which aim to naturalistically evaluate potential job candidates based on their resumes and interview performance, much as human recruiters do (17). To promote broad usefulness, LLMs undergo a complex, largely self-supervised training process very different from the fitting of conventional risk models (50, 51). This process is still not fully understood, and there is no guarantee that the hiring recommendations of conventional LLMs will correspond to statistically valid ex ante estimates of productivity. As a result, these systems, like the humans they emulate, have the potential to produce large risk-adjusted disparities.^¶¶^ While the widespread deployment of LLM-based decision tools promises to increase efficiency and reduce certain forms of human bias in the hiring process and beyond, disparate impact may remain a persistent problem.

In view of this form of discrimination’s continuing importance, we have sought to develop a simple, intuitive framework for formalizing and measuring disparate impact. Our approach, however, is subject to some important limitations. First, our method requires access to an outcome to estimate risk. In some instances, this information is not available to analysts. In other cases, it is not even clear how to rigorously define the relevant outcome. For example, in college admissions, decision makers often care about multiple factors in ways that are hard to quantify (5, 53). Second, to credibly estimate risk, analysts need sufficiently rich covariate data. Our sensitivity analysis helps mitigate omitted-variable bias, but it cannot replace better data. Third, and relatedly, analysts may not have access to race or other relevant protected characteristics, complicating the analysis. There has, however, been recent progress on estimating disparities using auxiliary data sources (e.g., refs. 54 and 55). Finally, we have modeled decisions using linear probability models with constant slope across groups. It is straightforward to estimate risk-adjusted disparities with more flexible decision models. However, relaxing this assumption makes it harder to gauge the sensitivity of the inferred disparities to inaccurate risk estimates.

Throughout our analysis, we have estimated “disparate impact” by a regression coefficient on protected-group identity in a model that adjusts for estimated risk. This procedure is analogous to current practice in discrimination studies, where we simply replace the usual set of control variables with a single variable capturing risk. As seen by our formalization of disparate impact in Eq. **2**, we are effectively measuring a particular weighted average of differences in decision rates across individuals of similar risk. While intuitively reasonable, this definition raises subtle questions of law and policy.

Consider, for example, Fig. 1, where we plot race-specific frisk rates as a function of risk. Stopped Black and Hispanic pedestrians are frisked more often than stopped White pedestrians at every level of risk. As a result, one would find that racial minorities face disparate impact regardless of how one averages across risk levels; the precise number might change, but the qualitative conclusion would remain the same. However, comparing Black and Hispanic pedestrians, the direction of the disparity depends on the risk level one considers. (Such a comparison between minority groups is unusual in disparate impact cases, but it illustrates the underlying theoretical issue.) Low-risk Hispanic individuals are frisked more often than low-risk Black individuals, but high-risk Black individuals are frisked more often than their high-risk Hispanic counterparts. Consequently, a conclusion of disparate impact between Black and Hispanic pedestrians would depend heavily on the precise definition applied. The analysis is further complicated if the risk distributions differ substantially between groups. If, hypothetically, stopped Hispanic pedestrians were mostly low-risk and stopped Black pedestrians mostly high-risk, majorities of both groups could argue that they were treated more harshly than members of the other group who were equally likely to be carrying a weapon.^##^

The crossing of risk-decision curves that we see in Fig. 1 is a potentially widespread phenomenon, and, to our knowledge, disparate impact law has not yet resolved the underlying conceptual ambiguity it invokes. Many discussions of disparate impact tacitly assume that policies either consistently harm or help groups defined by protected traits. Such thinking can be seen in the original *Griggs* ruling, where the Supreme Court aimed to proscribe policies that acted as “built-in headwinds” for racial minorities. But, formally, disparate impact law concerns facially race-neutral policies, not intentional discrimination. The Court has addressed related questions, for instance holding in *Connecticut v. Teal* (1982) that a particular stage of a selection process can result in disparate impact even if the process as a whole advantages a protected group(57). But there is no theoretical or empirical guarantee that any policy or stage of a selection process will adversely impact all members of a particular group.

A related issue is the extent to which concern for unjustified disparities compels decision makers to act optimally. For example, Fig. 1 suggests that officers are only marginally responsive to risk, with the lowest-risk individuals still frisked more than 40% of the time. If, instead, officers frisked only the people with high probability of carrying a weapon, they could frisk far fewer individual s—and, in particular, far fewer minority individuals—while recovering almost the same number of weapons (47). A more efficient frisk strategy could thus reduce the burdens of policing on racial minorities while still maintaining public safety. Such efficiency is indeed one of the aims of statistical risk assessment tools that are now used in the criminal justice system and beyond to guide high-stakes decisions (15, 56, 58,60). If these tools are shown to reduce racial disparities, are policymakers obliged—legally or ethically—to adopt them? The role of efficiency in disparate impact claims has largely gone unanswered by the courts, adding yet another subtlety to defining and measuring disparities. Researchers have only recently taken up these questions (15, 21, 61–63).

By foregrounding the role of risk in understanding disparities, we have aimed to clarify some of the thorny conceptual issues at the heart of disparate impact analysis. While there are still important unresolved questions, we believe that our statistical approach provides practitioners with a tractable way to assess disparities in many domains while avoiding some important pitfalls of traditional methods. Looking forward, we hope this work spurs further theoretical and empirical research on discrimination at the intersection of statistics, economics, law, and public policy.

## 4. Materials and Methods

To estimate risk of weapon possession in Section 2, we use gradient boosted decision trees, a nonlinear model popular in the machine learning community for its predictive performance, restricting to stops in the first subset of data in which a frisk was conducted. Predictive performance and model checks presented in *SI Appendix*, Fig. S10 indicate that the model yields predictions with reasonable performance and that predictions are well-calibrated across groups. The distribution of risk estimates produced by the model are shown in *SI Appendix*, Figs. S8 and S9. (*SI Appendix*, Figs. S11–S17 reproduce these results and the results in Figs. 1–3 with race excluded from the risk-prediction model.) For details on the sensitivity analysis, including parameter choices for the optimization, see *SI Appendix*, section 2.

## Supplementary Material

Appendix 01 (PDF)

## Data Availability

“Stop-question-frisk” records and reproduction materials for this analysis have been deposited in GitHub (64).

## Footnotes

^*^An important exception is adjusting for close proxies of protected characteristics, such as hair or eye color. For more detailed discussion of disparate treatment and disparate impact jurisprudence, see *SI Appendix*, section 1.

^†^The legal landscape surrounding disparate impact has recently shifted in the U.S., following an executive order deprioritizing disparate impact enforcement at the federal level (18). The order also revokes certain regulatory provisions that adopt a disparate impact framework, though statutory provisions such as Title VII of the Civil Rights Act governing disparate impact at the state and federal level remain in force.

^‡^Included-variable bias differs from post-treatment bias or what is sometimes termed “adjusting for bad controls” (24). In particular, the aim of the risk-adjusted regression framework we introduce is not to estimate a causal quantity, but rather the extent to which decisions are normatively or legally justified.

^§^Such problems have prompted a search for alternatives to regression-based approaches. Most prominently, Becker (25) proposed the outcome test, which is based not on the rate at which decisions are made, but on the success rate of those decisions. In the context of banking, Becker argued that even if, hypothetically, racial minorities were less creditworthy than White applicants, minorities who were granted loans should still be found to repay their loans at the same rate as White applicants who were granted loans. If loans to minorities had a higher repayment rate than loans to White borrowers, it suggests that lenders applied a double standard (intentionally or not), granting loans only to exceptionally qualified minorities—potentially violating disparate impact laws. The outcome-based approach has been applied almost as broadly as simple regression to study discrimination in, for example, policing (26–29), lending (30), and scientific publication (31). Becker’s outcome test, however, has significant statistical shortcomings, most notably the problem of inframarginality (28), which can lead the test to incorrectly suggest an absence of discrimination (32). Refinements of Becker’s test, such as the threshold test (33, 34), and, more recently, the robust outcome test (35) address inframarginality, but involve alternative assumptions and can be less informative than regression-based approaches when detailed covariate information is available.

^¶^In many cases, the expected net benefit is well captured by the probability of a particular outcome, which is why we call it risk. In lending, for example, it is common to approximate the profitability of loan by a loan applicant’s risk of default. In policing, searches are often motivated by the likelihood, i.e., risk, that an individual possesses contraband such as weapons or drugs. Our net benefit framing implicitly assumes that the underlying utility is justifiable. For example, a bank can reasonably seek to maximize profits, but their utility cannot reflect a “taste” for discrimination (36).

^#^To allow for the possibility of statistical discrimination (37–39) in the decision maker’s course of action, risk may be conditioned on group membership as well as other observables—formally, by including *C* among the covariates *X*. However, whether the inclusion of race or other protected attributes in risk models is legally appropriate is uncertain. Employers, for instance, are barred from statistically discriminating (40); statistical discrimination in medical decision making, however, is common if controversial (14, 16), though it has in some instances been successfully challenged in court (41). The empirical results in our main policing application remain essentially unchanged whether or not one assumes *X* encodes *C*; see *SI Appendix*, Figs. S11–S17.

^‖^To ensure this quantity is well defined, we assume that:

0<Pr(C=j∣R)<1, for all j=1,…,m.

This condition is immediately implied by an analogue of the standard overlap condition: 0<Pr(C=j∣X)<1 (42, 43).

^**^Our approach to estimating risk is not the only one, or even the best in any given instance. For example, when an appropriate sample exists, one could partially address this selection issue by leveraging some subpopulation of individuals that were randomly frisked, as is sometimes done at roadside checkpoints and airports. Alternatively, one could look for officers who frisk almost everyone they stop—similar to recent approaches for studying judicial decision-making (e.g., refs. 45 and 46). Both of these alternatives come with their own limitations, meaning that sensitivity analysis is still critical to credible inference.

^††^The curves show that even pedestrians with very low probabilities of carrying weapons have around a 40% probability of being frisked, indicating that officers’ frisk decisions are fairly inefficient. If officers frisked pedestrians according to a threshold rule, searching them if and only if their risk were above some cutoff, they could recover the same number of weapons while frisking fewer people (47).

^‡‡^We exclude hair color and eye color from the kitchen-sink model, since these are obvious proxies for race that are effectively unrelated to risk, and would thus be excluded in most traditional legal and statistical analyses of discrimination. As we would expect, including these variables as controls exacerbates the problem of included-variable bias, but our results show that such bias can occur even if obviously problematic variables are excluded.

^§§^Our approach, detailed in *SI Appendix*, section 2, is related to, but distinct from, well-known methods of sensitivity analysis in the causal inference literature that aim to gauge the impacts of unobserved confounding (49). In particular, as our statistical problem differs from that in the standard causal inference setting, those methods cannot be applied straightforwardly in our context. Our method, moreover, is particularly well-adapted to our setting, yielding reasonably tight bounds in practice that depend only on the interpretable parameter *ϵ*, without making additional assumptions about the form of the error ${\hat{R}}_{i}-R_{i}$. See *SI Appendix*, section 2 for further discussion of our method and alternatives.

^¶¶^In contrast, traditional supervised machine learning models trained to directly predict relevant outcomes (e.g., job performance) minimize risk-adjusted disparities by design—though one must take care to avoid “label bias” (52).

^##^Some scholars have similarly investigated interactions between race and other decision-making criteria. For example, Espenshade et al. (4) find that preferences for underrepresented minorities in college admissions is greatest for applicants with SAT scores in the 1200–1300 range, and the effect is attenuated at lower scores. That analysis, however, found no score ranges where minority applicants faced an absolute disadvantage relative to White applicants with equal scores. We do not know of any research that has found a change in the direction of the disparities like we see between Black and Hispanic pedestrians in Fig. 1.
