# Supplementary material for: Measuring disparate impact in human and machine decisions
Source: Proc Natl Acad Sci U S A. 2026 Jul 20;123(30):e2509765122. doi: 10.1073/pnas.2509765122 (PMC13416680; doi:10.1073/pnas.2509765122)
Supplement: Supplementary file 1 — Appendix 01 (PDF) [file pnas.2509765122.sapp.pdf]

PNAS

Supporting Information for

Measuring Disparate Impact in Human and Machine Decisions

Jongbin Jung, Sam Corbett-Davies, Johann D. Gaebler, Ravi Shroff, and Sharad Goel

Sharad Goel.
E-mail: sgoel@hks.harvard.edu

This PDF file includes:

Supporting text
Figs. S1 to S17
SI References

Supporting Information Text

1. Theories of Discrimination

We briefly review understandings of disparate treatment and disparate impact in U.S. law. In particular, first, we review the distinctions between disparate treatment and disparate impact, especially as it pertains to included-variable bias, and discuss risk-adjusted regression's role within the three-pronged legal test of disparate impact. Next, we give a simple formal illustration of included-variable bias in the context of *Griggs*. In the final subsection, we provide proofs of the mathematical content of our formal illustration.

1.A. Disparate treatment and disparate impact. Disparate treatment derives force from the Equal Protection Clause of the U.S. Constitution's Fourteenth Amendment, and, as noted in the main text, prohibits government agents from acting with "discriminatory purpose" (1). Although equal protection law bars policies undertaken with animus, it allows for the limited use of protected attributes to further a compelling government interest. For example, until recently, certain affirmative action programs for college admissions were legally permissible to further the government's interest in promoting diversity. In 2023, the U.S. Supreme Court overturned the legality of such affirmative action programs, ruling that it was unlawful to explicitly consider race in college admissions decisions (2).

The most widespread statistical test of such intentional discrimination is ordinary linear or logistic regression, in which one estimates the likelihood of favorable (or unfavorable) decisions across groups defined by race, gender, or other legally protected traits. In this approach, the investigator adjusts for all potentially relevant risk factors, excluding only clear proxies for the protected attributes. Barring omitted-variable bias, non-zero coefficients on the protected traits suggest those factors influenced the decision maker's actions; in the absence of a compelling justification, such evidence is suggestive of a discriminatory purpose. It is difficult—and perhaps impossible—to rigorously define the *influence*, or causal effect, of largely immutable traits like race on decisions (3, 4), but a regression of this type is nevertheless considered a reasonable first step to identify discriminatory motive, both by criminologists and by legal scholars (5, 6). For an equal protection claim to succeed in court, however, one typically needs additional documentary evidence (e.g., acknowledgement of an illegitimate motive) to bolster the statistical evidence.

Disparate impact, in contrast, inquires into a policy's effects, rather than the intentions of the legislature enacting it or the decision maker executing it. A practice is deemed discriminatory under this standard if it results in an "unjustified adverse impact" on a protected group, irrespective of the policy makers' intentions or whether protected traits play an explicit role in the administration of the policy. In *Griggs*, for instance, the Duke Power Company's motivation for instituting its education promotion requirement was irrelevant to the Court's decision; even if enacted without discriminatory purpose, the policy was deemed discriminatory in its effects and hence illegal. The doctrine stems from statutory rules, rather than constitutional law, and applies only in certain contexts, such as employment (via Title VII of the 1964 Civil Rights Act) and housing (via the Fair Housing Act of 1968). Apart from federal statutes, some states have passed more expansive disparate impact laws, including Illinois and California.

The legal test of disparate impact developed over half a century of case law has three principal elements (7):

1. **Adverse impact:** The plaintiff first must establish that the policy disproportionately impacts the minority group.

2. **No Justification:** Next, the defendant must establish that the adverse impact has a substantial justification rooted in a legitimate policy goal.

3. **Less discriminatory alternative:** Even if the disparate impact is justified, the plaintiff can nevertheless prevail if they demonstrate that there is an alternative feasible policy with less adverse impact on the minority group.

Risk-adjusted regression is concerned with the second element, namely, whether the adverse impact has some legitimate policy justification.

As discussed in the main text, the standard statistical test for disparate treatment is a "kitchen sink" regression, where one examines the residual explanatory power of protected group status after including all other available covariates as controls. Because it obscures the distinction between legitimate bases for differences in decisions and distinctions which are actually unrelated to the putative policy goal, "kitchen sink" regression is generally an inappropriate empirical method for measuring disparate impact. To assess claims of *unjustified* disparate impact, in, for example, *Griggs*, one would ideally compare decision rates for similarly *qualified* groups of applicants (e.g., similarly qualified White and Black candidates), rather than the more fine-grained comparison of applicants who are "similarly situated"—that is, generally similar, including in regards unrelated to their qualification. In general, effecting this comparison merely through the inclusion and exclusion of different regression covariates is difficult: If one does not, or cannot, adjust for sufficiently many covariates, omitted-variable bias may skew results; conversely, if one does adjust for a rich set of covariates, included-variable bias may corrupt conclusions.[1]

[1] A related body of work conceptualizes discrimination as race-specific differences in decision error rates (8–12). For example, one might consider differences in loan rejection rates between White and Black applicants who in reality would repay their loans (i.e., a counterfactual false negative rate). Recent work, however, has observed that by conditioning on *ex post* (potential) outcomes—as opposed to *ex ante* risk, as we do—such approaches suffer from inframarginality and associated statistical issues, rendering them problematic measures of common legal and policy understandings of discrimination (13–16).

1.B. A formal illustration of included-variable bias. We give a simple formal illustration of included-variable bias in the context of *Griggs v. Duke Power Co.* Consider the following data-generating process, depicted by the DAG below.

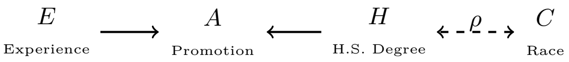

Here, C indicates the race of an employee, with $C = 0$ representing a White employee and $C = 1$ a Black employee; and H indicates whether an employee has a high-school degree. We assume that H and C have a joint distribution given by

$$\Pr(H = 1, C = 1) = \Pr(H = 0, C = 0) = \frac{1+\rho}{4}, \qquad \Pr(H = 1, C = 0) = \Pr(H = 0, C = 1) = \frac{1-\rho}{4}.$$

In particular, it follows that both H and C are marginally $\text{Bern}(\frac{1}{2})$ with correlation ρ, $-1 < \rho < 1$. Importantly, we need not assume that race C "causes" an employee to have a high school degree but only that race and having a high school degree have some correlation ρ (although, in this particular example, a causal mechanism like discriminatory enrollment policies is plausible).

Further, $E \sim \text{Unif}(0, 1)$ denotes an employee's level of experience, interpreted as the proportion of employees who have been employed for less time than them. Finally, by A, we denote whether the employee was promoted. Recalling the details of *Griggs*, we assume that

$$\Pr(A = 1 \mid E, H) = E \cdot H.$$

That is, only employees with high school degrees are promoted, and their probability of promotion depends on their experience.

A natural quantity to consider is the average difference in promotion rates for "similarly situated" Black and White employees—that is, the average difference in promotion rates for employees with the same experience level and high school degree. In this case, it is straightforward to show that

$$\mathbb{E}[\Pr(A = 1 \mid E, H, C = 1) - \Pr(A = 1 \mid E, H, C = 0)] = 0. \qquad [6]$$

Since race does not factor into promotion decisions, the average difference in hiring rates for similarly situated Black and White employees is zero, suggesting an absence of disparate *treatment*. However, if, following the Court's reasoning in *Griggs*, one were to consider the average difference in promotion rates for employees with the same *experience level*—regardless of whether they had a high school degree—one would obtain

$$\mathbb{E}[\Pr(A = 1 \mid E, C = 1) - \Pr(A = 1 \mid E, C = 0)] = \frac{\rho}{2}. \qquad [7]$$

Following the facts of *Griggs*, we expect $\rho < 0$, indicating that Black employees are less likely than White employees to have attained a high-school degree. As a result, as shown in Eq. (7), Black employees at the same level of experience are less likely to be promoted. In particular, this estimand captures the disparate *impact* of the promotion policy. This stylized example illustrates the importance of conditioning on the appropriate variables to avoid included-variable bias in studies of discrimination. Here, high-school degree attainment is inappropriate to condition on because it has little relationship to job performance.

Non-parametric estimands like those shown in Eqs. (6) and (7) can be statistically challenging to estimate in practice, so it is common instead to use linear regression or other parametric models to approximate these estimands. Imagine if an analyst were to fit a linear kitchen-sink regression model of A given experience level (E), high school degree (H), the interaction between these two terms ($E \cdot H$), and race (C),

$$\Pr(A = 1 \mid E, H, C) = \beta_0 + \beta_E \cdot E + \beta_H \cdot H + \beta_{E:H} \cdot E \cdot H + \beta_C \cdot C. \qquad [8]$$

Here, β_C is typically interpreted as the magnitude of "discrimination." In this example, $\hat{\beta}_C$ tends to zero in large samples, suggesting a lack of disparate *treatment*. But, despite fitting a correctly specified model of decisions, the analyst would fail to detect the disparate impact of the hiring policy.

Imagine, instead, that the analyst regressed decisions only on years of experience (E) and race (C), as the Court indicated they should, i.e.,

$$\Pr(A = 1 \mid E, C) = \beta'_0 + \beta'_E \cdot E + \beta'_C \cdot C. \qquad [9]$$

In this case, as with the non-parametric estimand in Eq. (7), $\hat{\beta}'_C$ tends to $\frac{\rho}{2}$, reflecting the fact that it correctly captures the disparate impact of the promotion policy. (In this specific example, the parametric and non-parametric estimands are identical, but this is not always the case.)

Following the Court's ruling, we have assumed that any use of education is *unjustified*, while use of experience is *justified* when making promotion decisions. As a result, the key expression of interest is that given in Eq. (7), which quantifies disparities in decision rates after conditioning on experience (E) alone. In practice, however, all covariates are usually at least weakly informative about one's qualifications. In the context of *Griggs*, high school degree attainment, even among employees with the same level of experience, is likely at least somewhat predictive of job performance. The more general definition in Eq. (1) provides one useful way of conceptualizing disparate impact in this more realistic setting.

1.C. Proofs. We begin by proving the results for the parametric formulation in Eqs. (8) and (9), and then use the parametric results to prove the non-parametric claims given in Eqs. (6) and (7).

1.C.1. Linear Regression Formulation. Recall that the OLS coefficient estimates are given in this case by

$$\left(\sum_{i=1}^{n}\begin{bmatrix} 1 & E_i & H_i & E_i\cdot H_i & C_i \\ E_i & E_i^2 & E_i\cdot H_i & E_i^2\cdot H_i & E_i\cdot C_i \\ H_i & E_i\cdot H_i & H_i^2 & E_i\cdot H_i^2 & H_i\cdot C_i \\ E_i\cdot H_i & E_i^2\cdot H_i & E_i\cdot H_i^2 & E_i^2\cdot H_i^2 & E_i\cdot H_i\cdot C_i \\ C_i & E_i\cdot C_i & H_i\cdot C_i & E_i\cdot H_i\cdot C_i & C_i^2 \end{bmatrix}\right)^{-1}\left(\sum_{i=1}^{n}\begin{bmatrix} A_i \\ E_i\cdot A_i \\ H_i\cdot A_i \\ E_i\cdot H_i\cdot A_i \\ C_i\cdot A_i \end{bmatrix}\right)$$

Note that because of the matrix inversion, we can divide both sums by n. Therefore, applying the continuous mapping theorem and the strong law of large numbers, we see that this converges to

$$\begin{bmatrix} 1 & \mathbb{E}[E] & \mathbb{E}[H] & \mathbb{E}[E\cdot H] & \mathbb{E}[C] \\ \mathbb{E}[E] & \mathbb{E}[E^2] & \mathbb{E}[E\cdot H] & \mathbb{E}[E^2\cdot H] & \mathbb{E}[E\cdot C] \\ \mathbb{E}[H] & \mathbb{E}[E\cdot H] & \mathbb{E}[H^2] & \mathbb{E}[E\cdot H^2] & \mathbb{E}[H\cdot C] \\ \mathbb{E}[E\cdot H] & \mathbb{E}[E^2\cdot H] & \mathbb{E}[E\cdot H^2] & \mathbb{E}[E^2\cdot H^2] & \mathbb{E}[E\cdot H\cdot C] \\ \mathbb{E}[C] & \mathbb{E}[E\cdot C] & \mathbb{E}[H\cdot C] & \mathbb{E}[E\cdot H\cdot C] & \mathbb{E}[C^2] \end{bmatrix}^{-1}\begin{bmatrix} \mathbb{E}[A] \\ \mathbb{E}[E\cdot A] \\ \mathbb{E}[H\cdot A] \\ \mathbb{E}[E\cdot H\cdot A] \\ \mathbb{E}[C\cdot A] \end{bmatrix}.$$

Now, recalling the joint and marginal distributions of these variables, we have that

$$\mathbb{E}[E] = \frac{1}{2}, \qquad \mathbb{E}[H] = \frac{1}{2}, \qquad \mathbb{E}[C] = \frac{1}{2}.$$

Moreover,

$$\mathbb{E}[E^2] = \frac{1}{3}, \qquad \mathbb{E}[H^2] = \frac{1}{2}, \qquad \mathbb{E}[C^2] = \frac{1}{2}.$$

By the definition of covariance,

$$\rho = \frac{\mathbb{E}[H\cdot C] - \mathbb{E}[H]\cdot\mathbb{E}[C]}{\sqrt{\mathrm{Var}(H)\cdot\mathrm{Var}(C)}} = \frac{\mathbb{E}[H\cdot C] - \frac{1}{4}}{\sqrt{\frac{1}{4}\cdot\frac{1}{4}}} = 4\cdot\mathbb{E}[H\cdot C] - 1,$$

whence $\mathbb{E}[H\cdot C] = \frac{\rho+1}{4}$. By independence,

$$\mathbb{E}[E\cdot H] = \mathbb{E}[E]\cdot\mathbb{E}[H] = \frac{1}{4}, \qquad \mathbb{E}[E\cdot C] = \mathbb{E}[E]\cdot\mathbb{E}[C] = \frac{1}{4},$$

$$\mathbb{E}[E^2\cdot H] = \mathbb{E}[E^2]\cdot\mathbb{E}[H] = \frac{1}{6}, \qquad \mathbb{E}[E\cdot H^2] = \mathbb{E}[E\cdot H] = \frac{1}{4},$$

$$\mathbb{E}[E^2\cdot H^2] = \mathbb{E}[E^2\cdot H] = \frac{1}{6}, \qquad \mathbb{E}[E\cdot H\cdot C] = \mathbb{E}[E]\cdot\mathbb{E}[H\cdot C] = \frac{\rho+1}{8},$$

Finally,

$$\begin{aligned}\mathbb{E}[A] &= \mathbb{E}[\mathbb{E}[A \mid E, H]] \\ &= \mathbb{E}[E\cdot H] \\ &= \frac{1}{4},\end{aligned} \qquad \begin{aligned}\mathbb{E}[E\cdot A] &= \mathbb{E}[\mathbb{E}[E\cdot A \mid E, H]] \\ &= \mathbb{E}[E^2\cdot H] \\ &= \frac{1}{6},\end{aligned}$$

$$\begin{aligned}\mathbb{E}[H\cdot A] &= \mathbb{E}[\mathbb{E}[H\cdot A \mid E, H]] \\ &= \mathbb{E}[E\cdot H^2] \\ &= \frac{1}{4},\end{aligned} \qquad \begin{aligned}\mathbb{E}[E\cdot H\cdot A] &= \mathbb{E}[\mathbb{E}[E\cdot H\cdot A \mid E, H]] \\ &= \mathbb{E}[E^2\cdot H^2] \\ &= \frac{1}{6},\end{aligned}$$

$$\begin{aligned}\mathbb{E}[C\cdot A] &= \mathbb{E}[\mathbb{E}[C\cdot A \mid E, H, C]] \\ &= \mathbb{E}[E\cdot H\cdot C] \\ &= \frac{\rho+1}{8}.\end{aligned}$$

Thus, we have that

$$\begin{bmatrix} 1 & \mathbb{E}[E] & \mathbb{E}[H] & \mathbb{E}[E\cdot H] & \mathbb{E}[C] \\ \mathbb{E}[E] & \mathbb{E}[E^2] & \mathbb{E}[E\cdot H] & \mathbb{E}[E^2\cdot H] & \mathbb{E}[E\cdot C] \\ \mathbb{E}[H] & \mathbb{E}[E\cdot H] & \mathbb{E}[H^2] & \mathbb{E}[E\cdot H^2] & \mathbb{E}[H\cdot C] \\ \mathbb{E}[E\cdot H] & \mathbb{E}[E^2\cdot H] & \mathbb{E}[E\cdot H^2] & \mathbb{E}[E^2\cdot H^2] & \mathbb{E}[E\cdot H\cdot C] \\ \mathbb{E}[C] & \mathbb{E}[E\cdot C] & \mathbb{E}[H\cdot C] & \mathbb{E}[E\cdot H\cdot C] & \mathbb{E}[C^2] \end{bmatrix}^{-1}$$

equals

$$\begin{bmatrix} 1 & \frac{1}{2} & \frac{1}{2} & \frac{1}{4} & \frac{1}{2} \\ \frac{1}{2} & \frac{1}{3} & \frac{1}{4} & \frac{1}{6} & \frac{1}{4} \\ \frac{1}{2} & \frac{1}{4} & \frac{1}{2} & \frac{1}{4} & \frac{\rho+1}{4} \\ \frac{1}{4} & \frac{1}{6} & \frac{1}{4} & \frac{1}{6} & \frac{\rho+1}{8} \\ \frac{1}{2} & \frac{1}{4} & \frac{\rho+1}{4} & \frac{\rho+1}{8} & \frac{1}{2} \end{bmatrix}^{-1} = \begin{bmatrix} \frac{7\rho+9}{\rho+1} & -12 & -\frac{6\rho+8}{\rho+1} & 12 & -\frac{2}{\rho+1} \\ -12 & 24 & 12 & -24 & 0 \\ -\frac{6\rho+8}{\rho+1} & 12 & \frac{12\rho^2-16}{\rho^2-1} & -24 & \frac{4\rho}{\rho^2-1} \\ 12 & -24 & -24 & 48 & 0 \\ -\frac{2}{\rho+1} & 0 & \frac{4\rho}{\rho^2-1} & 0 & -\frac{4}{\rho^2-1} \end{bmatrix}$$

and so the OLS regression coefficients converge almost surely to

$$\begin{bmatrix} \frac{7\rho+9}{\rho+1} & -12 & -\frac{6\rho+8}{\rho+1} & 12 & -\frac{2}{\rho+1} \\ -12 & 24 & 12 & -24 & 0 \\ -\frac{6\rho+8}{\rho+1} & 12 & \frac{12\rho^2-16}{\rho^2-1} & -24 & \frac{4\rho}{\rho^2-1} \\ 12 & -24 & -24 & 48 & 0 \\ -\frac{2}{\rho+1} & 0 & \frac{4\rho}{\rho^2-1} & 0 & -\frac{4}{\rho^2-1} \end{bmatrix} \begin{bmatrix} \frac{1}{4} \\ \frac{1}{6} \\ \frac{1}{4} \\ \frac{1}{6} \\ \frac{\rho+1}{8} \end{bmatrix} = \begin{bmatrix} 0 \\ 0 \\ 0 \\ 1 \\ 0 \end{bmatrix}.$$

On the other hand, if the analyst were to drop high school graduation from their regression, then we would have that the regression coefficients converge almost surely to

$$\begin{bmatrix} 1 & \mathbb{E}[E] & \mathbb{E}[C] \\ \mathbb{E}[E] & \mathbb{E}[E^2] & \mathbb{E}[E \cdot C] \\ \mathbb{E}[C] & \mathbb{E}[E \cdot C] & \mathbb{E}[C^2] \end{bmatrix}^{-1} \begin{bmatrix} \mathbb{E}[A] \\ \mathbb{E}[E \cdot A] \\ \mathbb{E}[C \cdot A] \end{bmatrix} = \begin{bmatrix} 1 & \frac{1}{2} & \frac{1}{2} \\ \frac{1}{2} & \frac{1}{3} & \frac{1}{4} \\ \frac{1}{2} & \frac{1}{4} & \frac{1}{2} \end{bmatrix}^{-1} \begin{bmatrix} \frac{1}{4} \\ \frac{1}{6} \\ \frac{\rho+1}{8} \end{bmatrix} = \begin{bmatrix} -\frac{\rho}{4} \\ \frac{1}{2} \\ \frac{\rho}{2} \end{bmatrix}.$$

1.C.2. Non-Parametric Formulation. Recall the two different non-parametric estimands:

$$\mathbb{E}[\mathbb{E}[A \mid E, H, C = 1] - \mathbb{E}[A \mid E, H, C = 0]]$$

and

$$\mathbb{E}[\mathbb{E}[A \mid E, C = 1] - \mathbb{E}[A \mid E, C = 0]].$$

We note that in the first case, since $\mathbb{E}[A \mid E, H, C] = E \cdot H$, we have that

$$\mathbb{E}[\mathbb{E}[A \mid E, H, C = 1] - \mathbb{E}[A \mid E, H, C = 0]] = \mathbb{E}[E \cdot H - E \cdot H] = 0.$$

In the second case, using the expectations calculated above, we have that

$$\begin{aligned} \mathbb{E}[A \mid E, C = c] &= \mathbb{E}[A \mid E, H = 1, C = c] \cdot \Pr(H = 1 \mid E, C = c) \\ &\quad + \mathbb{E}[A \mid E, H = 0, C = c] \cdot \Pr(H = 0 \mid E, C = c) \\ &= E \cdot \Pr(H = 1 \mid E, C = c) \\ &= E \cdot \Pr(H = 1 \mid C = c) \\ &= E \cdot \frac{\mathbb{E}[H \cdot \mathbf{1}(C = c)]}{\mathbb{E}[\mathbf{1}(C = c)]} \\ &= E \cdot \frac{\frac{1-(-1)^c \cdot \rho}{4}}{\frac{1}{2}} \\ &= E \cdot \frac{1 - (-1)^c \cdot \rho}{2}. \end{aligned}$$

Here we have used the fact that $A = 0$ when $H = 0$ in the second equality, the independence of E from H and C in the third, and various expectations calculated above in the remaining equalities. Thus, it follows that

$$\mathbb{E}[\mathbb{E}[A \mid E, C = 1] - \mathbb{E}[A \mid E, C = 0]] = \mathbb{E}\left[E \cdot \frac{1+\rho}{2} - E \cdot \frac{1-\rho}{2}\right] = \mathbb{E}[E \cdot \rho] = \frac{\rho}{2}.$$

This exactly equals β'_C in Eq. (9).

2. Sensitivity analysis

In the following appendix, we develop our method for gauging the sensitivity of risk-adjusted regression to omitted-variable bias in the analyst's risk estimates. We discuss this sensitivity analysis in two parts. First, in Section 2.A, we motivate our approach to sensitivity analysis in risk-adjusted regression, more carefully introducing our formal setting, and laying out the key mathematical results underpinning our method. Then, in Section 2.B, we prove our method's correctness and derive bounds on its complexity and the accuracy of the approximations it makes use of.

2.A. Introduction.

2.A.1. Formal Setup. We model the data-generating process as consisting of draws of tuples $(X, \tilde{X}, C, W, A)$ where $X \in \mathcal{X}$, $\tilde{X} \in \tilde{\mathcal{X}}$, $C \in \{1, \ldots, m\}$, $W \in \{0, 1\}$, and $A \in \{0, 1\}$. Here A is a binary decision (either human or algorithmic), X is the set of covariates on which that decision is based, and C represents membership in some protected class. The binary variable W represents a latent property of interest to the decision maker—discussed further below. However, we assume the decision is made based on X alone, i.e.,

$$A \perp\!\!\!\perp W \mid X.$$

Finally, we assume that an analyst observes the tuple $(\tilde{X}, C, A, A \cdot W)$, based on which they seek to estimate disparate impact in the decision process. Here $\tilde{X}$ represents an alternative set of covariates available to the analyst that may differ from X in arbitrary ways. Additionally, the term $A \cdot W$ reflects the fact that W is only observed by the analyst when action $A = 1$ occurs. As noted in the main text, this general mathematical framework applies to a wide variety of policy contexts in which a decision maker, based on partial information X, aims to make a decision A targeting some binary outcome W.

2.A.2. Overview. Our definition of disparate impact in Eq. (2) depends on the true risks, R_i. But, in practice, analysts observe only partial information on X and W, and so at best can construct only imperfect estimates of risks, $\hat{R}_i$. For instance, in our running police example, officers might base search decisions in part on subtle behavioral cues that are not documented in the data; and analysts typically would not know whether individuals who were *not* searched were in fact carrying a weapon. In Section 3, in the main text, we discuss various approaches to estimating risk in light of these challenges. Whatever approach one adopts, the accuracy of one's conclusions depends critically on the accuracy of one's estimates of risk. To address this issue, we develop a novel sensitivity analysis (implemented in the `rar` `R` package on CRAN) that draws inspiration from methods for sensitivity analysis popular in the causal inference literature (17, 18)—though we emphasize that our framework does not itself involve estimating causal effects.

To start, we recall Eq. (5), in which we assumed that there is a known constant $\epsilon \geq 0$ such that

$$\frac{1}{n} \sum_{i=1}^{n} |R_i - \hat{R}_i| \leq \epsilon;$$

that is, that the true risks and the estimated risks differ on average by at most ϵ.[2] Given this assumption, the goal of our sensitivity analysis is to understand how different our estimate of disparate impact, $\hat{\beta}_j - \hat{\beta}_1$, could be if we had access to the true risk R_i instead of the estimated risk $\hat{R}_i$. In practice, an analyst would examine the robustness of conclusions to different choices of ϵ.

We could attempt to bound our estimate of disparate impact by searching over all possible choices of R_i satisfying the constraint in Eq. (5). But such an approach is overly conservative, as the observed data themselves rule out possible values of R_i. In our policing example, for instance, we expect the average true risk of searched individuals to approximately equal the proportion of searched individuals carrying a weapon. Since, by assumption, the observed data tell us about this latter quantity, they constrain the possible risk distributions we must consider.

To formalize our approach, a key quantity to consider is the average (true) risk on each stratum,

$$\frac{1}{n_{j,a}} \sum_{i \in \mathcal{S}_{j,a}} R_i, \qquad [10]$$

where

$$\mathcal{S}_{j,a} = \{\, i : C_i = j, A_i = a \,\}, \qquad n_{j,a} = |\mathcal{S}_{j,a}|,$$

i.e., $\mathcal{S}_{j,a}$ denotes the stratum containing all those individuals in group j for whom the decision maker took action a, and $n_{j,a}$ is its size. By the law of large numbers, the average risk on stratum $\mathcal{S}_{j,a}$ is approximated by $\mathbb{E}[R \mid C = j, A = a]$. Further,

$$\begin{aligned}\mathbb{E}[R \mid C = j, A = a] &= \mathbb{E}[\mathbb{E}[W \mid X] \mid C = j, A = a] \\ &= \mathbb{E}[\mathbb{E}[W \mid X, C, A] \mid C = j, A = a] \\ &= \mathbb{E}[W \mid C = j, A = a],\end{aligned}$$

[2] These differences could arise due to either measurement or modelling error. However, since measurement error tends to vanish as the sample size increases, it is less of a concern, and is better captured through bootstrapped bounds on the sensitivity analysis, as we discuss below.

where the first equality follows by definition; the second from $A \perp\!\!\!\perp W \mid X$ if we assume that C is a function of X; and the third by the law of iterated expectations. When $A = 1$, by our assumption in Section 2, the above quantity is identified by data available to the analyst. In particular, in our policing example, a consistent estimator of $\mathbb{E}[W \mid C = j, A = 1]$ is the proportion of searched individuals possessing a weapon, among those individuals belonging to group j. We denote by ρ_j the analyst's estimate of $\mathbb{E}[W \mid C = j, A = 1]$ based on the observed data.[3]

Finally, putting the pieces together, for each particular j^* we seek the largest and smallest values of $\hat{\beta}_{j^*} - \hat{\beta}_1$, as estimated with R_i, subject to two key constraints: (1) the average absolute deviation between R_i and $\hat{R}_i$ is less than ϵ; and (2) R_i is consistent with the observed data on each stratum $\mathcal{S}_{j,1}$. This is succinctly expressed as an optimization problem (henceforth **"the base problem"**):

$$\begin{aligned} \underset{\mathbf{R}\in\mathbb{R}^n}{\text{Optimize}} \quad & \hat{\beta}_{j^*} - \hat{\beta}_1 \\ \text{s.t.} \quad & \tfrac{1}{n}\textstyle\sum_{i=1}^n |R_i - \hat{R}_i| \le \epsilon \\ & \tfrac{1}{n_{j,1}}\textstyle\sum_{i\in\mathcal{S}_{j,1}} R_i = \rho_j, \ (j = 1, \ldots, m) \\ & R_i \le u_i, \ \ (i = 1, \ldots, n) \\ & R_i \ge \ell_i, \ \ (i = 1, \ldots, n) \end{aligned} \qquad [11]$$

where, by "Optimize," we simply mean both maximize and minimize, and $\hat{R}_i$ and ρ_j are fixed by the data as discussed above.[4] The additional upper and lower bounds on R_i arise from the fact that the risks must be probabilities, i.e., $0 \le R_i \le 1$, though our approach accommodates tighter, individual-specific bounds $\ell_i \le R_i \le u_i$ set by the analyst.

Below, we give a polynomial time approximation to the solution of the base problem when the constraints are "sortable." Sortability is a mild hypothesis which holds in the typical case, $\ell_i = 0$ and $u_i = 1$ for all i. It also holds if, e.g., ℓ_i and u_i differ from $\hat{R}_i$ by the additive constant Γ on the log odds scale, as is common in many kinds of sensitivity analysis (e.g., 20).

Definition S1 (Sortability). We say that the *constraints are sortable* if there exists a permutation π of $\{1, \ldots, n\}$ such that $\hat{R}_{\pi(1)} \le \ldots \le \hat{R}_{\pi(n)}$, $\ell_{\pi(1)} \le \ldots \le \ell_{\pi(n)}$, and $u_{\pi(1)} \le \ldots \le u_{\pi(n)}$.

There are two main obstacles to solving the base problem above. First, the dimension of the search space is $n - m$, where n is the number of observations and m is the number of groups. Second, the regression coefficients $\hat{\beta}_j$ are non-convex functions of the true risk vector $\mathbf{R} = (R_1, \ldots, R_n)$. Addressing these twin challenges involves a detailed analysis of the underlying geometry of the problem. Here we present an outline of our approach that illustrates the key ideas, with the full exposition in SI 2.B.

It is useful to think of the base problem as an optimization over subproblems (henceforth **"the parameterized problem"**) where the average (true) risk is assumed known and equal to some τ_j for *unobserved* strata $\mathcal{S}_{j,0}$ as well. Using the closed form of OLS regression, we can show that solving the parameterized problem reduces to—although is not equivalent to—solving the following optimization problem (henceforth **"the simplified problem"**):

$$\begin{aligned} \underset{\mathbf{R}\in\mathbb{R}^n}{\text{Optimize}} \quad & \frac{1}{n}\sum_{i=1}^n R_i^2 \\ \text{s.t.} \quad & \tfrac{1}{n}\textstyle\sum_{i=1}^n |R_i - \hat{R}_i| \le \epsilon, \\ & \tfrac{1}{n_{j,1}} \cdot \textstyle\sum_{i\in\mathcal{S}_{j,1}} R_i = \rho_j, \ (j = 1, \ldots, m) \\ & \tfrac{1}{n_{j,0}} \cdot \textstyle\sum_{i\in\mathcal{S}_{j,0}} R_i = \tau_j, \ (j = 1, \ldots, m) \\ & R_i \le u_i, \ \ (i = 1, \ldots, n) \\ & R_i \ge \ell_i. \ \ (i = 1, \ldots, n) \end{aligned} \qquad [12]$$

An efficient method for solving the simplified problem then yields an efficient method for solving the base problem simply by searching over this much smaller m-dimensional parameter space of $(\tau_1, \ldots, \tau_m)$. In practice, m typically equals two or three, and so this final optimization step can be solved either using a grid search, as we do in Section 3, or using other optimization techniques for low-dimensional non-convex problems.

The objective of the simplified problem, $\frac{1}{n}\sum_{i=1}^n R_i^2$, is convex, as are the other constraints on $\mathbf{R}$, and so, in principle, minimization in the simplified problem can be achieved efficiently and accurately using interior-point methods (21). However, by carefully examining the KKT conditions (22, 23), it is possible to see that the minimizing risk vector must adhere to a special form, which we term "minimization normal form." Specifically, there exist thresholds $t_{j,a}^{\text{lwr}}$ and $t_{j,a}^{\text{upr}}$ for each stratum such that the minimizing R_i is obtained by "pulling up" low estimated risks to $t_{j,a}^{\text{lwr}}$, "pulling down" high estimated risks to $t_{j,a}^{\text{upr}}$, and leaving unchanged intermediate risks. An example of a risk vector in minimization normal form is shown in Figure S1.

This observation reduces the dimension of the search space from $n - 2m$ to $2m$; that is, by the above, one need only search over the $2m$ thresholds. One can, however, do better. A careful examination of the KKT conditions reveals that while the

[3] One might alternatively estimate $\mathbb{E}[W \mid C = j, A = 1]$ by the average *estimated* risks on the stratum, namely $\frac{1}{|\mathcal{S}_{j,1}|} \cdot \sum_{i\in\mathcal{S}_{j,1}} \hat{R}_i$. This alternative, which we adopt in our empirical analysis in Section 3, has the interpretive advantage that our sensitivity analysis exactly recovers the original analysis when $\epsilon = 0$. In cases where risk is not conditioned on C, then true risks need not be calibrated within groups. As a result, the average estimated risk may approximate the average true risk less well. For instance, this might be the case when decision makers are legally prohibited from considering race. In our policing example, however, the estimated risks are essentially unchanged by the exclusion or inclusion of race, suggesting that the impact of potential miscalibration is small.

[4] It may seem inappropriate to fix the average risks on the observed stratum $\mathcal{S}_{j,1}$ to be exactly ρ_j, since ρ_j is estimated with error. One could instead allow ρ_j to vary within some range (e.g., a 95% confidence interval), solving the parameterized problem for various ρ_j as well as τ_j, as detailed below. However, doing so doubles the dimension of the search space, which is computationally costly, and does not fully take advantage of available information about the distribution of the estimation error. A more principled and computationally feasible approach to dealing with estimation error is to bootstrap confidence intervals for the bounds following Zhao et al. (19), as we do in Section B below, where ρ_j is re-estimated in each bootstrapped resample, and the base problem is solved with corresponding equality constraints.

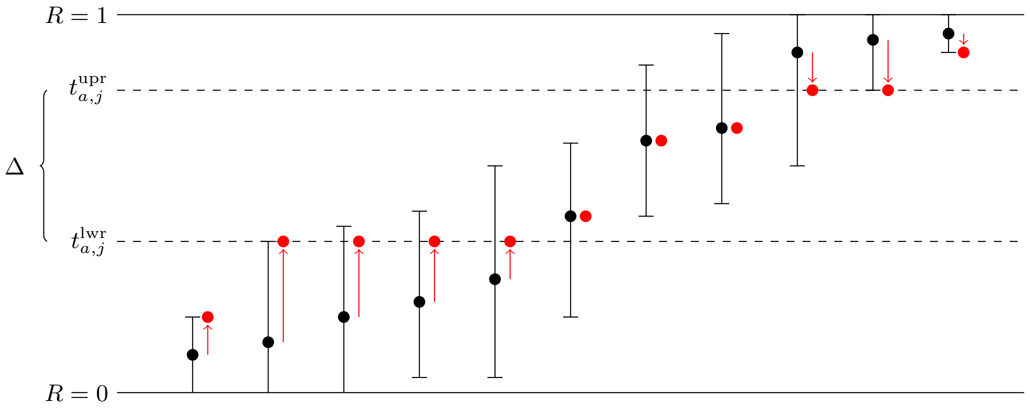

Fig. S1. *Minimization normal form. Black dots represent estimated risks $\hat{R}_i$, the error bars represent the allowable range $[\ell_i, u_i]$, and red dots indicate the corresponding value of the normal form vector R_i. Here Δ denotes the gap between the upper and lower thresholds.*

thresholds $t^{\mathrm{lwr}}_{j,a}$ and $t^{\mathrm{upr}}_{j,a}$ may vary by stratum, the gap between them $\Delta = t^{\mathrm{upr}}_{j,a} - t^{\mathrm{lwr}}_{j,a}$ must be the same across strata. Moreover, once Δ is fixed, the thresholds $t^{\mathrm{lwr}}_{j,a}$ and $t^{\mathrm{upr}}_{j,a}$ are determined by the strata-specific average risks ρ_j and τ_j. As a result, finding the minimizing risk vector for the simplified problem reduces to optimizing over a single parameter, Δ, which, in our setting, can be done very efficiently. In particular, we give an $O(n \cdot \log(m))$ algorithm that finds the exact solution.

Maximization requires more care, since the maximization problem is non-convex, and the general problem of maximizing a quadratic objective over a convex set is NP-hard (24). Using a similar, but more delicate, version of the techniques used to minimize the simplified problem, we give an $O(m \cdot (\epsilon/\gamma)^2 + n)$-time algorithm for approximating the maximum of the simplified problem. The key steps described above for optimizing the base problem are given in Algorithm 1.

Putting these results together, we have the following theorem, the proof of which constitutes the majority of SI 2.B.

Theorem S2. *Suppose the constraints are sortable. Consider Algorithm 1 with step-size parameters η and γ and maximum average absolute deviation ϵ. Let δ^* denote the true optimum of the base problem, and let $\delta^\dagger$ denote the value of the objective returned by Algorithm 1. Then, there exists a constant c_0 such that Algorithm 1 runs in time at most*

$$c_0 \cdot \left(n \cdot \log(n) + m/\gamma^2\right) \cdot \eta^{-m}.$$

Moreover, there exists a constant c_1 and a problem-specific constant $V(\epsilon)$ (i.e., depending on $\hat{R}_i$, ℓ_i, u_i, and ϵ) such that

$$|\delta^* - \delta^\dagger| \leq \frac{c_1 \cdot (\eta + m\gamma)}{V(\epsilon)^2}.$$

Here η denotes the step size of a grid search over average risks of the unobserved strata, and γ denotes the step size of a discrete grid of values of ϵ in the maximization routine. We take $\eta = 0.05$ p.p. and $\gamma = 0.01$ p.p. in Section 3. We give the definition of $V(\epsilon)$ in Eq. (35) in SI 2.B. Roughly, $V(\epsilon)$ is related to the within-group variance of the estimated risks V,

$$V = \frac{1}{n} \sum_{j=1}^{m} n_j \cdot \mathrm{VAR}\left((\hat{R}_i)_{i \in \mathcal{G}_j}\right).$$

In particular, $V(\epsilon) \geq V - 4\epsilon$. Moreover, $V(\epsilon)$ is positive whenever the base problem has a finite solution. In our principal application, it takes a few seconds on standard hardware for the algorithm to run for 100 values of ϵ with $m = 3$ race groups and $n > 10^6$ observations with negligible approximation error.

2.A.3. Comparison with other methods. As we note above, our approach to sensitivity analysis for risk-adjusted regression is related to sensitivity analysis methods developed in the causal inference literature, particularly to recent work on L_2 sensitivity bounds (25, 26). Our approach, however, is distinct in important ways that make it uniquely advantageous in our setting. First, methods for sensitivity analysis in the causal inference literature that can be adapted to our setting often involve a large number of parameters—making them difficult to credibly calibrate—or require parametric assumptions on the form of the confounding itself (e.g., 17, 18, 20). In contrast, the single parameter in our sensitivity analysis—ϵ, the average absolute deviation between the true and estimated risks—is straightforward to reason about and explain, particularly to policymakers or other non-technical stakeholders.

The popular sensitivity analysis developed by Cinelli and Hazlett (27), which makes use of two sensitivity parameters, readily conforms to the linear estimation strategy in Eq. (3). In our setting, it gauges how much $\hat{\beta}_{j^*} - \hat{\beta}_1$ changes between the following two regression specifications:

$$\sum_{j=1}^{m} \hat{\beta}_j \cdot \mathbf{1}(C_i = j) + \hat{\beta}_{\hat{R}} \cdot \hat{R}_i \qquad \text{and} \qquad \sum_{j=1}^{m} \hat{\beta}_j \cdot \mathbf{1}(C_i = j) + \hat{\beta}_{\hat{R}} \cdot \hat{R}_i + \hat{\beta}_R \cdot R_i.$$

Algorithm 1 (Sensitivity analysis)

Input: The estimated risk vector $\hat{\mathbf{R}}$, the lower and upper bounds $\boldsymbol{\ell}$ and $\mathbf{u}$, the average absolute difference ϵ, and the step-size parameters η and γ.
Output: The minimum and maximum values of $\hat{\beta}_j - \hat{\beta}_1$ for all groups $j = 2, \ldots, m$.

Set $\rho_j \leftarrow \frac{1}{|\mathcal{S}_{j,1}|} \cdot \sum_{i \in \mathcal{S}_{j,1}} \hat{R}_i$ for $j = 1, \ldots, m$
Generate a grid of $(\tau_1, \ldots, \tau_m)$ with step size η
repeat
  Calculate $\mathbf{R}^*_{\text{min}}$ minimizing the simplified problem with $\rho_1, \ldots, \rho_m$ and $\tau_1, \ldots, \tau_m$
  Calculate $\mathbf{R}^*_{\text{max}}$ maximizing the simplified problem to within $2m\gamma$ with $\rho_1, \ldots, \rho_m$ and $\tau_1, \ldots, \tau_m$
  Calculate $\hat{\beta}_j - \hat{\beta}_1$ using both $\mathbf{R}^*_{\text{min}}$ and $\mathbf{R}^*_{\text{max}}$
until The grid of $(\tau_1, \ldots, \tau_m)$ is exhausted
return The largest and smallest differences $\hat{\beta}_j - \hat{\beta}_1$ observed for all $j = 2, \ldots, m$

However, the sensitivity analysis's goal is to compare the former specification to the "true" regression specification in Eq. (3), $\sum_{j=1}^{m} \hat{\beta}_j \cdot \mathbf{1}(C_i = j) + \hat{\beta}_R \cdot R_i$. This can be effected when the coefficient $\hat{\beta}_{\hat{R}}$ is zero in the latter specification, i.e., when decisions (A) are independent of estimated risk $(\hat{R})$ given true risk (R) and group membership (C).[5] This assumption is implausible, though, when the influence of various factors on decision makers deviates from the factors' actual connection to risk, typically the key concern in studies of disparate impact.[6]

Other existing sensitivity methods that do involve a single parameter would, if suitably adapted to our setting, be parameterized in terms of an L_∞-bound Γ on the log odds ratio between true and estimated risks (e.g., 19, 28). However, in realistic models of confounding, the change in the odds ratio typically cannot be bounded in this way for all units: some—potentially very small—set of units will have an estimated risk of approximately 0 but a true risk of approximately 1 or *vice versa*. As a result, one may need to set Γ to be very large to strictly satisfy the assumptions of the approach, yielding sensitivity bounds that are, in practice, quite conservative. More recent methods involving L_2 bounds avoid this problem (25, 26), but would not yield tight bounds on the estimand of interest in our setting.

2.B. Theory. In what follows, we formally justify the sensitivity analysis laid out in SI 2.A, deriving algorithms to efficiently solve the optimization problem it gives rise to and rigorously verifying their correctness and run-times. This discussion is structured in four parts. In the first part, by introducing a new low-dimensional family of parameters, we show how to reduce the base optimization problem of Eq. (11) into the simplified problem of Eq. (12). In the second and third parts, we show how to efficiently find the minimum and maximum values of the simplified optimization problem, respectively. Finally, in the fourth part, we prove bounds on the quality of the approximation.

Remark S3. In SI 2.A, for expositional clarity, we parameterized our optimization problems in terms of *average* risks. However, it is mathematically more natural to work with the *total* risks. Therefore, throughout this appendix we let ρ_j and τ_j denote the *total* risk in each stratum, i.e., we rewrite the relevant constraints of the base problem and the simplified problem as follows:

$$\sum_{i \in \mathcal{S}_{j,1}} R_i = \rho_j, \qquad \sum_{i \in \mathcal{S}_{j,0}} R_i = \tau_j.$$

2.B.1. Simplifying the objective. To simplify the optimization problem in Eq. (11), we can avail ourselves of the fact that OLS has a closed-form solution. We augment the notation in SI 2.A above as follows:

$$\mathcal{G}_j = \mathcal{S}_{j,0} \cup \mathcal{S}_{j,1} = \{\, i : C_i = j \,\},$$

i.e., $\mathcal{G}_j$ denotes the set of indices belonging to the j-th group.

Lemma S4. *Let $n_j = |\mathcal{G}_j| > 0$ denote the number of individuals in the j-th group; let σ_j denote the search rate in group j, i.e., $\sigma_j = \frac{1}{n_j} \sum_{i \in \mathcal{G}_j} A_i$; and let r_j and t_j denote*

$$r_j = \sum_{i \in \mathcal{S}_{j,1}} R_i, \qquad t_j = \sum_{i \in \mathcal{S}_{j,0}} R_i,$$

i.e., the total risk of the searched and unsearched individuals belonging to the j-th group, respectively.

[5] Strictly speaking, it is required that the residuals of $\hat{\mathbf{R}}$ and $\mathbf{A}$, when regressed against $\mathbf{R}$ and $\mathbf{C}$, must be uncorrelated, a mathematically weaker condition that the independence assumption implies.

[6] As a simple example, suppose officers over-weight certain factors like "furtive movements" that are relatively uninformative about risk, as discussed in the main text. In this case, individuals who made furtive movements would be over-represented in the frisked population, relative to their risk. Consequently, in the more limited data available to the analyst, furtive movements would likely be associated with a *lower* chance of possessing a weapon, all else equal. Thus, conditional on true risk R and group C, individuals with lower predicted risks $\hat{R}$ would have greater odds of having engaged in furtive movements. Since officers over-weight furtive movements when making frisk decisions, lower predicted risk would consequently be associated with a higher chance of having been frisked, even conditional on R and C.

If R_i is constant within each of the m groups, then the OLS estimate need not exist due to collinearity. Otherwise, the OLS estimate of the coefficients $\hat{\boldsymbol{\beta}}$ in Eq. (3) *satisfies*

$$\hat{\boldsymbol{\beta}} = \begin{bmatrix} \sigma_1 \\ \vdots \\ \sigma_m \\ 0 \end{bmatrix} + \frac{\frac{1}{n}\left[\sum_{j=1}^m \sigma_j \cdot t_j - (1-\sigma_j)\cdot r_j\right]}{\frac{1}{n}\left[\sum_{i=1}^n R_i^2 - \sum_{j=1}^m \frac{(r_j+t_j)^2}{n_j}\right]} \begin{bmatrix} \frac{r_1+t_1}{n_1} \\ \vdots \\ \frac{r_m+t_m}{n_m} \\ -1 \end{bmatrix}. \qquad [13]$$

Proof. Recall that our design matrix and outcome variables take the form

$$\mathbf{X} = \begin{bmatrix} \mathbf{1}(C_1 = 1) & \dots & \mathbf{1}(C_1 = m) & R_1 \\ \vdots & \ddots & \vdots & \vdots \\ \mathbf{1}(C_n = 1) & \dots & \mathbf{1}(C_n = m) & R_n \end{bmatrix}, \qquad \mathbf{Y} = \begin{bmatrix} A_1 \\ \vdots \\ A_n \end{bmatrix}.$$

The OLS estimate of the coefficients $\hat{\boldsymbol{\beta}}$ is then simply $(\mathbf{X}^\top\mathbf{X})^{-1}\mathbf{X}^\top\mathbf{Y}$.

Note that by definition, we have that

$$\mathbf{X}^\top\mathbf{X} = \begin{bmatrix} n_1 & & & \sum_{i\in\mathcal{G}_1} R_i \\ & \ddots & & \vdots \\ & & n_m & \sum_{i\in\mathcal{G}_m} R_i \\ \sum_{i\in\mathcal{G}_1} R_i & \dots & \sum_{i\in\mathcal{G}_m} R_i & \sum_{i=1}^n R_i^2 \end{bmatrix} = \begin{bmatrix} n_1 & & & r_1+t_1 \\ & \ddots & & \vdots \\ & & n_m & r_m+t_m \\ r_1+t_1 & \dots & r_m+t_m & \sum_{i=1}^n R_i^2 \end{bmatrix}.$$

Similarly,

$$\mathbf{X}^\top\mathbf{Y} = \begin{bmatrix} \sum_{i\in\mathcal{G}_1} A_i \\ \vdots \\ \sum_{i\in\mathcal{G}_m} A_i \\ \sum_{i=1}^n R_i \cdot A_i \end{bmatrix} = \begin{bmatrix} n_1\cdot\sigma_1 \\ \vdots \\ n_m\cdot\sigma_m \\ \sum_{j=1}^m r_j \end{bmatrix}.$$

Note that $\mathbf{X}^\top\mathbf{X}$ has the form

$$\begin{bmatrix} D & v^\top \\ v & \sum_{i=1}^n R_i^2 \end{bmatrix},$$

where D is diagonal. Then, we see that since D is invertible, it follows that $\mathbf{X}^\top\mathbf{X}$ is invertible if the Schur complement of the D is invertible, i.e., non-zero; that is, if

$$\sum_{i=1}^n R_i^2 - \sum_{j=1}^m \frac{(r_j+t_j)^2}{n_j} \neq 0.$$

Recalling the definitions of r_j and t_j, we see that the left-hand expression equals

$$\sum_{j=1}^m \sum_{i\in\mathcal{G}_j} \left(R_i - \frac{r_j+t_j}{n_j}\right)^2 = \sum_{j=1}^m n_j \cdot \textsc{Var}\left((R_i)_{i\in\mathcal{G}_j}\right).$$

Now, the variance of a set is only zero if all the elements of the set are equal, i.e., if for all $i \in \mathcal{G}_j$, R_i equals the average risk in that group, $\frac{r_j+t_j}{n_j}$.

Assume this is not the case, i.e., that $\mathbf{X}^\top\mathbf{X}$ is invertible. Since $\mathbf{X}^\top\mathbf{X}$ is an arrowhead matrix, we can invert it using the Sherman-Morrison formula. In particular, we obtain that

$$(\mathbf{X}^\top\mathbf{X})^{-1} = \begin{bmatrix} \frac{1}{n_1} & & & \\ & \ddots & & \\ & & \frac{1}{n_m} & \\ & & & 0 \end{bmatrix} + \frac{1}{\sum_{i=1}^n R_i^2 - \sum_{j=1}^m \frac{(r_j+t_j)^2}{n_j}} \begin{bmatrix} \frac{r_1+t_1}{n_1} \\ \vdots \\ \frac{r_m+t_m}{n_m} \\ -1 \end{bmatrix} \begin{bmatrix} \frac{r_1+t_1}{n_1} \\ \vdots \\ \frac{r_m+t_m}{n_m} \\ -1 \end{bmatrix}^\top. \qquad [14]$$

Combining this with the expression derived for $\mathbf{X}^\top\mathbf{Y}$ above and dividing the numerator and denominator of the scalar term by n yields that

$$\hat{\boldsymbol{\beta}} = \begin{bmatrix} \sigma_1 \\ \vdots \\ \sigma_m \\ 0 \end{bmatrix} + \frac{\frac{1}{n}\left[\sum_{j=1}^m \sigma_j \cdot t_j - (1-\sigma_j)\cdot r_j\right]}{\frac{1}{n}\left[\sum_{i=1}^n R_i^2 - \sum_{j=1}^m \frac{(r_j+t_j)^2}{n_j}\right]} \begin{bmatrix} \frac{r_1+t_1}{n_1} \\ \vdots \\ \frac{r_m+t_m}{n_m} \\ -1 \end{bmatrix}. \qquad [15]$$

□

We note that Lemma S4 provides the following useful condition on when the base problem has a meaningful solution:

Corollary S5. *The maximum and minimum values of the base problem are both finite if and only if it is not the case that* $\ell_i \le \rho_j \le u_i$ *for all* $i \in \mathcal{G}_j$ *and* $j = 1, \ldots, m$*, and in addition*

$$\frac{1}{n}\sum_{j=1}^{m}\sum_{i\in\mathcal{G}_j}\left|\hat{R}_i - \frac{\rho_j}{n_{j,1}}\right| \le \epsilon.$$

Proof. The proof follows immediately from noting that since the averages of the risks on the observed strata $\mathcal{S}_{j,1}$ are fixed at ρ_j, the risks for the whole j-th group can only all be equal if the risks of the observed *and* the unobserved individuals are uniformly equal to ρ_j. □

The following corollary completes the simplification of the optimization problem in Eq. (11) in the case where the average true risk in the unobserved strata is also assumed known (**"the parameterized problem"** of SI 2.A),[7] i.e., of

$$\begin{aligned}\underset{\mathbf{R}\in\mathbb{R}^n}{\text{Optimize}} \quad & \hat{\beta}_{j^*} - \hat{\beta}_1 \\ \text{s.t.} \quad & \tfrac{1}{n}\textstyle\sum_{i=1}^{n}|R_i - \hat{R}_i| \le \epsilon, \\ & \textstyle\sum_{i\in\mathcal{S}_{j,1}} R_i = \rho_j,\ (j = 1,\ldots,m) \\ & \textstyle\sum_{i\in\mathcal{S}_{j,0}} R_i = \tau_j,\ (j = 1,\ldots,m) \\ & R_i \le u_i,\ \ (i = 1,\ldots,n) \\ & R_i \ge \ell_i.\ \ (i = 1,\ldots,n)\end{aligned} \qquad [16]$$

Corollary S6. *The optimal solutions of the parameterized problem in* Eq. (16)*, if they are both well-defined, are also the optimal solutions of the simplified problem in* Eq. (12)*.*

Proof. Let $\hat{\boldsymbol{\beta}}$ be as in Eq. (3), and r_j and t_j as in Lemma S4.[8] Then, it follows directly from Eq. (13) that we have that for all $\mathbf{R}$,

$$\hat{\beta}_j - \hat{\beta}_1 = \sigma_j - \sigma_1 + \frac{\frac{1}{n}\left[\sum_{j=1}^{m}\sigma_j\cdot t_j - (1-\sigma_j)\cdot r_j\right]}{\frac{1}{n}\left[\sum_{i=1}^{n}R_i^2 - \sum_{j=1}^{m}\frac{(r_j+t_j)^2}{n_j}\right]}\left(\frac{r_j+t_j}{n_j} - \frac{r_1+t_1}{n_1}\right).$$

Since the constraints of the parameterized problem fix $r_j = \rho_j$ and $t_j = \tau_j$ for all feasible $\mathbf{R}$, the objective function of the parameterized problem has the form

$$a + \frac{b}{x-c}, \qquad [17]$$

where

$$\begin{aligned} a &= \sigma_j - \sigma_1, \\ b &= \frac{1}{n}\left[\sum_{j=1}^{m}\sigma_j\cdot\tau_j - (1-\sigma_j)\cdot\rho_j\right]\cdot\left(\frac{\rho_j+\tau_j}{n_j} - \frac{\rho_1+\tau_1}{n_1}\right), \\ c &= \frac{1}{n}\sum_{j=1}^{m}\frac{(\rho_j+\tau_j)^2}{n_j}, \\ x &= \frac{1}{n}\sum_{i=1}^{n}R_i^2. \end{aligned}$$

If $b = 0$, then the objective does not depend on $\mathbf{R}$, and so there is nothing to prove. Otherwise, by continuity and the fact that the feasible region is convex, and hence connected, and compact, the range of the objective in Eq. (12) over the feasible region is a closed interval I. As shown in the proof of Lemma S4 and Corollary S5, $x \ge c$, and $x > c$ if the optima are both well-defined. Consequently, we can assume the interval is strictly positive, i.e., $I \subseteq (c, \infty)$. The derivative of Eq. (17) exists everywhere on I and is, moreover, non-zero and continuous, and so $a + \frac{b}{x-c}$ is strictly monotone on I. Therefore, the maximum and minimum must occur at the endpoints, i.e., at the maximum and minimum values of x. □

We note that since b is not guaranteed to be positive, the maximum of the simplified problem in Eq. (12) does not necessarily correspond to the maximum of the base problem in Eq. (11) and *vice versa*.

[7] See Remark S3 above for the slight difference in notation between here and SI 2.A.

[8] In particular, strictly speaking, $\hat{\boldsymbol{\beta}}$, r_j, and t_j should be written $\hat{\boldsymbol{\beta}}(\mathbf{R})$, $r_j(\mathbf{R})$, and $t_j(\mathbf{R})$, since they depend on $\mathbf{R}$.

2.B.2. Minimization. In the case of minimization, the simplified problem in Eq. (12) defines a convex optimization problem, which, as noted in SI 2.A above, can consequently be solved efficiently using standard interior-point methods. However, these results can be improved in a problem-specific way to yield a simpler, and, in practice, more computationally tractable method of finding exact solutions. Moreover, they parallel the solution method for maximization, which is not a convex problem, and hence to which standard interior point methods cannot be applied.

Optimizing over a single stratum We begin by solving a simpler version of the problem, where we restrict to a single stratum. In the case of minimization—as in the case of maximization, as we show below—solutions to the minimization problem must be in a certain normal form. While searching over all risk vectors is prohibitively difficult, searching over normal form risk vectors can be done straightforwardly in linear time.

Definition S7 (Minimization normal form). We say that a risk vector $\mathbf{R}$ is in *minimization normal form* if there exist thresholds $t^{\text{lwr}}_{a,j}$ and $t^{\text{upr}}_{a,j}$ such that for $i \in \mathcal{S}_{j,a}$,

$$R_i = \begin{cases} \min(u_i, t^{\text{lwr}}_{a,j}) & \hat{R}_i \le t^{\text{lwr}}_{a,j}, \\ \hat{R}_i & t^{\text{lwr}}_{a,j} \le \hat{R}_i \le t^{\text{upr}}_{a,j}, \\ \max(\ell_i, t^{\text{upr}}_{a,j}) & t^{\text{upr}}_{a,j} \le \hat{R}_i, \end{cases}$$

and, in addition, $t^{\text{upr}}_{a,j} - t^{\text{lwr}}_{a,j}$ is equal to some Δ for all $a = 0, 1$ and $j = 1, \dots, m$.

In the context of a single stratum, we refer to t^{upr} and t^{lwr} for notational simplicity. We also assume that $\mathcal{S}_{j,a} = \{1, \dots, n\}$ for the same reason.

Minimization normal form results from "pushing" $\hat{R}_i$ up to t^{lwr} or down to t^{upr} as far as possible. An example of a risk vector in minimization normal form is shown in Figure S1. As the name suggests, risk vectors minimizing the objective must be in minimization normal form.

Lemma S8. *Consider the optimization problem (**"the single stratum minimization problem"**)*

$$\begin{aligned} \underset{\mathbf{R}\in\mathbb{R}^n}{\text{Minimize}} \quad & \frac{1}{n}\sum_{i=1}^{n} R_i^2 \\ \text{s.t.} \quad & \tfrac{1}{n}\textstyle\sum_{i=1}^{n} |R_i - \hat{R}_i| \le \epsilon, \\ & \textstyle\sum_{i=1}^{n} R_i = \mu, \\ & R_i \le u_i, \ (\forall i) \\ & R_i \ge \ell_i. \ (\forall i) \end{aligned} \qquad [18]$$

Suppose that $\mathbf{R}^*$ is a minimizer. Then, $\mathbf{R}^*$ is unique and in minimization normal form. Moreover, $\mathbf{R}^*$ exhausts the L_1 budget, in the sense that either $\frac{1}{n}\|\mathbf{R}^* - \hat{\mathbf{R}}\|_1 = \epsilon$ or $t^{\text{lwr}} = t^{\text{upr}}$.

Proof. The proof proceeds by examining the first-order KKT conditions to derive weak necessary conditions that solutions must satisfy. These conditions are then strengthened to minimization normal form by directly comparing the objective function at different points satisfying the weak conditions. Finally, we show that the minimum is the point satisfying the "strengthened" conditions which is most distant from $\hat{\mathbf{R}}$, i.e., that exhausts the budget.

The problem is simplified by rewriting the first constraint as a collection of linear (and hence everywhere differentiable) constraints. In particular, $\frac{1}{n}\|\mathbf{R} - \hat{\mathbf{R}}\|_1 \le \epsilon$ is equivalent to the 2^n constraints of the form $\mathbf{S}^\top(\mathbf{R} - \hat{\mathbf{R}}) \le \epsilon \cdot n$ for all $\mathbf{S} \in \{-1, 1\}^n$. We assume throughout, without loss of generality, that $\ell_i < u_i$.

Weak conditions Let $\mathbf{e}_i$ denote the i-th standard basis vector and $\mathbf{1} = \sum_{i=1}^n \mathbf{e}_i$. Note that:

- The gradient of the objective is $\frac{2}{n} \cdot \mathbf{R}$;
- The gradient of $\mathbf{S}_k^\top(\mathbf{R} - \hat{\mathbf{R}}) - \frac{\epsilon}{n}$, where $\{\mathbf{S}_1, \dots, \mathbf{S}_{2^n}\} = \{-1, 1\}^n$, is $\mathbf{S}_k$;
- The gradient of $\sum_{i=1}^n R_i$ is $\mathbf{1}$;
- The gradient of $R_i - u_i$ is $\mathbf{e}_i$;
- The gradient of $\ell_i - R_i$ is $-\mathbf{e}_i$.

The first-order necessary KKT conditions therefore require that[9]

$$2 \cdot \mathbf{R}^* - \lambda \cdot \mathbf{1} + \sum_{i=1}^{n} \mu_{0,i} \cdot \mathbf{e}_i - \sum_{i=1}^{n} \mu_{1,i} \cdot \mathbf{e}_i + \sum_{k=1}^{2^n} \nu_k \cdot \mathbf{S}_k = 0 \qquad [19]$$

for some arbitrary λ, and for non-negative $\mu_{0,i}$, $\mu_{1,i}$, and ν_k for $k = 1, \dots, 2^n$ satisfying complementary slackness, i.e., such that $\mu_{0,i} \cdot (R_i^* - u_i) = 0$, $\mu_{1,i} \cdot (\ell_i - R_i^*) = 0$, and $\nu_k \cdot (\mathbf{S}_k^\top(\mathbf{R}^* - \hat{\mathbf{R}}) - n \cdot \epsilon) = 0$ for all $i = 1, \dots, n$ and $k = 1, \dots, 2^n$.

[9] For notational simplicity, we have multiplied through by n and absorbed constants into the corresponding Lagrange multipliers.

These consequences allow us to prove the following weak characterization of $\mathbf{R}^*$. Let $\Delta = \sum_{k=1}^{2^n} \nu_k$. Then, for all i,

$$R_i^* \in \left\{\hat{R}_i, u_i, \ell_i, \frac{1}{2}\cdot[\lambda - \Delta], \frac{1}{2}\cdot[\lambda+\Delta]\right\}. \qquad [20]$$

For, from Eq. (19), we get that

$$2 \cdot R_i^* - \lambda + \mu_{0,i} - \mu_{1,i} + \sum_{k=1}^{2^n} \nu_k \cdot S_{k,i} = 0, \qquad [21]$$

where $S_{k,i}$ refers to the i-th component of $\mathbf{S}_k$. If $R_i^* = \hat{R}_i$, there is nothing to prove. Therefore, assume that $R_i^* \neq \hat{R}_i$. We note that by complementary slackness, $\nu_k > 0$ only if $S_{k,i} \cdot (R_i^* - \hat{R}_i) \geq 0$ for all $i = 1, \ldots, n$. For, supposing without loss of generality that $R_i^* > \hat{R}_i$, if $S_{k,i} \cdot (R_i^* - \hat{R}_i) < 0$, then, since $\mathbf{S}_k + 2\mathbf{e}_i \in \{-1, 1\}^n$, we would have that

$$(\mathbf{S}_k + 2\mathbf{e}_i)^\top (\mathbf{R}^* - \hat{\mathbf{R}}) = \epsilon \cdot n + 2(R_i^* - \hat{R}_i) > \epsilon \cdot n,$$

which violates the constraints. Hence $S_{k,i} = S_{k',i}$ for all k and k' such that $\nu_k, \nu_{k'} > 0$. Therefore, our expression simplifies to

$$2 \cdot R_i^* - \lambda + \mu_{0,i} - \mu_{1,i} + \Delta \cdot s = 0,$$

where $s = \pm 1$. By complementary slackness, if $\mu_{0,i} > 0$, then $R_i^* = u_i$; if $\mu_{1,i} > 0$, then $R_i^* = \ell_i$. Therefore, we need only consider the case where $\mu_{0,i} = \mu_{1,i} = 0$, i.e., where

$$2 \cdot R_i^* - \lambda + s \cdot \Delta = 0,$$

whence $R_i^* = \frac{1}{2}[\lambda + s \cdot \Delta]$. Therefore, for all i, Eq. (20) holds.

Strengthening conditions Next, we strengthen Eq. (20) to minimization normal form. First, we show that if $R_{i_0}^* > \hat{R}_{i_0}$ and $R_{i_0}^* > R_{i_1}^*$, then $R_{i_1}^* = u_{i_1}$. For, suppose not. Then, there exists some $\delta > 0$ such that (1) $R_{i_0}^* - \delta > \hat{R}_{i_0}$, (2) $R_{i_1}^* + \delta < u_{i_1}$, and (3) $R_{i_0}^* - \delta > R_{i_1}^*$. Define $\mathbf{R}' = \mathbf{R}^* + \delta \cdot (\mathbf{e}_{i_1} - \mathbf{e}_{i_0})$. Then, we note that

$$\begin{aligned}
\|\mathbf{R}' - \hat{\mathbf{R}}\|_1 - \|\mathbf{R}^* - \hat{\mathbf{R}}\|_1 &= \sum_{i=1}^n |R_i' - \hat{R}_i| - |R_i^* - \hat{R}_i| \\
&= |R_{i_0}' - \hat{R}_{i_0}| - |R_{i_0}^* - \hat{R}_{i_0}| + |R_{i_1}' - \hat{R}_{i_1}| - |R_{i_1}^* - \hat{R}_{i_1}| \\
&= (R_{i_0}^* - \delta - \hat{R}_{i_0}) - (R_{i_0}^* - \hat{R}_{i_0}) + |R_{i_1}^* + \delta - \hat{R}_{i_1}| - |R_{i_1}^* - \hat{R}_{i_1}| \\
&\leq -\delta + |R_{i_1}^* - \hat{R}_{i_1}| + \delta - |R_{i_1}^* - \hat{R}_{i_1}| \\
&= 0,
\end{aligned}$$

so $\mathbf{R}'$ satisfies the L_1-distance constraint. Here, we have used the fact that both $R_{i_0}^*$ and $R_{i_0}^* - \delta$ are greater than $\hat{R}_{i_0}$ in the third equality, and the triangle inequality in the inequality. The remaining constraints also hold by construction, so $\mathbf{R}'$ is feasible.

Since $\mathbf{R}'$ is feasible, we will arrive at a contradiction if we can show that it achieves a smaller objective. However,

$$\begin{aligned}
\sum_{i=1}^n (R_i^*)^2 - (R_i')^2 &= (R_{i_0}^*)^2 - ((R_{i_0}^*)^2 + \delta^2 - 2 \cdot R_{i_0}^* \cdot \delta) + (R_{i_1}^*)^2 \\
&\quad - ((R_{i_1}^*)^2 + \delta^2 + 2 \cdot (R_{i_1}^*) \cdot \delta) + \sum_{i \neq i_0, i_1} (R_i^*)^2 - (R_i^*)^2 \\
&= 2 \cdot \delta \cdot (R_{i_0}^* - R_{i_1}^* - \delta) \\
&> 0,
\end{aligned}$$

where the inequality follows from the fact that $R_{i_0}^* - \delta > R_{i_1}^*$. Therefore $\mathbf{R}^*$ is not a minimum, contrary to hypothesis. In exactly the same manner, we see that if $R_{i_0}^* < \hat{R}_i$ and $R_{i_1}^* < R_{i_0}^*$, then $R_{i_1}^* = \ell_{i_1}$.

To apply the claim, let s^{lwr} be the maximum R_i^* for i such that $R_i^* > \hat{R}_i$, and similarly for s^{upr}. Then, for all i such that $R_i^* < s^{\text{lwr}}$, $R_i^* = u_i$; similarly, where $R_i^* > s^{\text{upr}}$, $R_i^* = \ell_i$. By Eq. (20), we must have that $s^{\text{lwr}} \leq s^{\text{upr}}$, since otherwise there would be some i such that $R_i^* = u_i$ and $R_i^* = \ell_i$, which is impossible. Therefore, in particular, by construction, $R_i^* = \hat{R}_i$ whenever $s^{\text{lwr}} < R_i^* < s^{\text{upr}}$. Thus, taking $t^{\text{lwr}} = s^{\text{lwr}}$ and $t^{\text{upr}} = s^{\text{upr}}$ gives the desired normal form.

Uniqueness and budget exhaustion If the budget is not exhausted, then, by complementary slackness, $\nu_k = 0$ for all $k = 1, \ldots, 2^n$, whence $\Delta = 0$. This immediately implies that $t^{\text{lwr}} = t^{\text{upr}} = \frac{1}{2}\lambda$.

To see uniqueness, suppose that $\mathbf{R}^{(0)}$ and $\mathbf{R}^{(1)}$ are two distinct minima, both in normal form, with corresponding thresholds t_k^{lwr} and t_k^{upr} for $k = 0, 1$. We note that since $\sum_{i=1}^n R_i^{(0)} = \sum_{i=1}^n R_i^{(1)} = \mu$, we must have, without loss of generality, that $t_0^{\text{lwr}} \leq t_1^{\text{lwr}}$ and $t_0^{\text{upr}} \geq t_1^{\text{upr}}$, with at least one of the two inequalities strict. However, it follows that $\|\mathbf{R}^{(0)} - \hat{\mathbf{R}}\|_1 < \|\mathbf{R}^{(1)} - \hat{\mathbf{R}}\|_1$, which is impossible by the preceding paragraph. □

Lemma S8 suggests a natural algorithm for solving the optimization problem in Eq. (12): we can sweep over all possible risk vectors in minimization normal form simply by sweeping over t^{lwr}. In particular, as we increase t^{lwr}, the sum constraint—i.e., that $\sum_{i=1}^n R_i = \mu$—forces t^{upr} to decrease so as to counterbalance it exactly.

More precisely, consider an index i to be "active" if one of the thresholds is between ℓ_i and u_i. If k^{lwr} represents the number of indices that are active because of t^{lwr}—and k^{upr} is also defined accordingly—then, locally, if we increase t^{lwr} at unit rate, the sum of the risks increases at the rate of k^{lwr}. Therefore, the rates of increase of the two thresholds, r^{lwr} and r^{upr}, must satisfy[10]

$$r^{\text{lwr}} \cdot k^{\text{lwr}} = r^{\text{upr}} \cdot k^{\text{upr}}. \tag{22}$$

In particular, we know that we have reached the minimum—and hence solved the optimization problem—once either the budget has been exhausted or the two thresholds have become equal. For fixed r^{lwr} and r^{upr}, this would be straightforward to calculate; however, these rates can, in principle, change whenever k^{upr} and k^{lwr} change. We call these change points "distinguished points"—that is, points at which a threshold t equals ℓ_i, $\hat{R}_i$, or u_i for some i. Therefore, the algorithm consists of repeating the following steps:

1. Determining the "next" distinguished point that either the lower or upper threshold will reach.

2. Checking whether the budget will be exhausted before that point is reached.

3. If not, advancing to that point and recalculating k^{lwr} or k^{upr} as appropriate.

In addition, since $\sum_{i=1}^n \hat{R}_i$ may not equal μ, a "preprocessing" step, where t^{lwr} is increased or t^{upr} is decreased may be needed to find the "initial" risk vector in minimization normal form satisfying the sum constraint.

We make two observations about the informal algorithm sketch given above needed for its extension to the case of multiple strata. First, we note that every risk vector $\mathbf{R}^*$ in minimization normal form is the minimizer for the corresponding single stratum minimization problem with $\epsilon = \frac{1}{n}\|\mathbf{R}^* - \hat{\mathbf{R}}\|_1$ and $\mu = \sum_{i=1}^n R_i^*$. Consequently, although intended to find the minimizer for a specific ϵ, the algorithm sketched above actually sweeps over the minimizers for *all possible* ϵ. As a result, it is not any harder to solve a single instance of the single stratum minimization problem than it is to solve it generally for all possible ϵ, given some fixed μ. Secondly, while the natural way to parameterize the risk vectors in the discussion above is in terms of t^{lwr}, when optimizing over multiple strata, it is actually more natural to reparameterize the risk vectors in terms of the gap $\Delta = t^{\text{upr}} - t^{\text{lwr}}$. If in addition to Eq. (22) we impose the condition that $r^{\text{lwr}} + r^{\text{upr}} = 1$, so that Δ decreases at a constant rate, then we have that

$$r^{\text{lwr}} = \frac{k^{\text{upr}}}{k^{\text{lwr}} + k^{\text{upr}}}, \qquad r^{\text{upr}} = \frac{k^{\text{lwr}}}{k^{\text{lwr}} + k^{\text{upr}}}. \tag{23}$$

To solve the single stratum minimization problem across the whole range of possible ϵ, let $\mathbf{R}_\Delta$ be the unique solution we can associate with a given gap Δ between t^{lwr} and t^{upr}.[11] Then, we can study two functions:

$$\epsilon(\Delta) = \|\mathbf{R}_\Delta - \hat{\mathbf{R}}\|_1, \qquad \Sigma(\Delta) = \sum_{i=1}^n R_{\Delta,i}^2 - \hat{R}_i^2.$$

By the preceding discussion we readily derive that $\epsilon(\Delta)$ is a piecewise linear function of Δ, whose slope at a given value of Δ is equal to[12]

$$K = 2 \cdot \frac{k^{\text{lwr}} \cdot k^{\text{upr}}}{k^{\text{lwr}} + k^{\text{upr}}},$$

and $\Sigma(\Delta)$ is a piecewise quadratic function of Δ satisfying

$$\begin{aligned}
\Sigma(\Delta + t) &= \Sigma(\Delta) + k^{\text{lwr}} \cdot [(t^{\text{lwr}} + r^{\text{lwr}} \cdot t)^2 - (t^{\text{lwr}})^2] + k^{\text{upr}} \cdot [(t^{\text{upr}} - r^{\text{upr}} \cdot t)^2 - (t^{\text{upr}})^2] \\
&= \Sigma(\Delta) + k^{\text{lwr}} \cdot r^{\text{lwr}} \cdot t \cdot (r^{\text{lwr}} \cdot t + 2t^{\text{lwr}}) + k^{\text{upr}} \cdot r^{\text{upr}} \cdot t \cdot (r^{\text{upr}} \cdot t - 2t^{\text{upr}}) \\
&= \Sigma(\Delta) - 2 \cdot \frac{k^{\text{lwr}} \cdot k^{\text{upr}}}{k^{\text{lwr}} + k^{\text{upr}}} \cdot (t^{\text{upr}} - t^{\text{lwr}}) \cdot t + \frac{k^{\text{lwr}}(k^{\text{upr}})^2 + k^{\text{upr}}(k^{\text{lwr}})^2}{(k^{\text{lwr}} + k^{\text{upr}})^2} \cdot t^2 \\
&= \Sigma(\Delta) - K\Delta t + \frac{K}{2} t^2,
\end{aligned}$$

as long as t is sufficiently small that the number of active indices does not change. It follows that $\epsilon(\Delta)$ and $\Sigma(\Delta)$ are fully determined by the following collections:

$$\boldsymbol{\Delta} = (\Delta_1, \ldots, \Delta_N), \qquad 1 = \Delta_1 > \cdots > \Delta_N = 0,$$

[10] If either k^{lwr} or k^{upr} is zero, then the corresponding threshold can be changed at any rate without affecting the sum constraint. (The rate of change for the other threshold will consequently be zero.) However, this is because changing that threshold in this case does not actually change the risk vector, since no indices are active, and so we can ignore this case in the subsequent discussion.

[11] For every Δ there is a unique $\mathbf{R}_\Delta$. In particular, any other distinct risk vector in minimization normal form with the same gap must be strictly greater or lesser, as argued at the end of Lemma S8, meaning that it cannot satisfy the sum constraint. However, the reverse is not necessarily true—that is, $\mathbf{R}_\Delta$ may equal $\mathbf{R}_{\Delta'}$ for $\Delta \neq \Delta'$—since achieving a given gap may require that $k^{\text{lwr}} = k^{\text{upr}} = 0$, in which case different thresholds can be chosen without altering the underlying risk vector.

[12] Or zero if both k^{lwr} and k^{upr} are zero.

where Δ_k represents the k-th value of Δ at which k^{lwr} and k^{upr} change; and

$$\mathbf{K} = (K_1, \ldots, K_N), \qquad K_1 = 0, \quad K_N = 0,$$

where K_k denotes the value of K beginning at $\Delta = \Delta_k$. From $\boldsymbol{\Delta}$ and $\mathbf{K}$, the Δ^* such that $\|\mathbf{R}_{\Delta^*} - \hat{\mathbf{R}}\|_1 = \epsilon$ can be calculated in linear time. For completeness, this algorithm is given in Algorithm 2.

Thus, a complete solution for the optimization problem for a single stratum across *all possible* ϵ requires only calculating $\boldsymbol{\Delta}$ and $\mathbf{K}$. As described above, this can be calculated by moving the thresholds toward each other at the prescribed rates r^{lwr} and r^{upr}, updating the rates each time an index is activated or deactivated, until the gap between the thresholds is zero. Similar to evaluating $\epsilon(\Delta)$ and $\Sigma(\Delta)$, this can be completed in linear time, as described in Algorithm 4. (As above, we note that if $\frac{1}{n}\sum_{i=1}^n \hat{R}_i \neq \mu$, it may be necessary to do a preprocessing step, also in linear time, as shown in Algorithm 3.)

Optimizing over all strata With a complete solution to the problem of minimizing the sum of squares for a single stratum, the problem of minimizing the sum of squares across all strata is straightforward. We begin by characterizing solutions to the minimization problem in the general case.

Lemma S9. *Consider the simplified minimization problem in* Eq. (12)*. Suppose that $\mathbf{R}^*$ is a solution. Then, the restriction of $\mathbf{R}^*$ to any stratum is in minimization normal form. Moreover, $\mathbf{R}^*$ exhausts the L_1 budget, in that either $\frac{1}{n}\|\mathbf{R}^* - \hat{\mathbf{R}}\|_1 = \epsilon$ or $t^{\text{lwr}} = t^{\text{upr}} = \mu_{a,j}$, where $\mu_{a,j}$ equals either ρ_j or τ_j depending on whether a equals 1 or 0.*

Proof. The proof is virtually identical to the proof of Lemma S8. The only difference is that the first-order necessary KKT conditions take the form

$$2 \cdot \mathbf{R}^* - \left(\sum_{j=1}^m \lambda_{0,j} \cdot \mathbf{1}_{0,j} + \lambda_{1,j} \cdot \mathbf{1}_{1,j} \right) + \sum_{i=1}^n \mu_{0,i} \cdot \mathbf{e}_i - \sum_{i=1}^n \mu_{1,i} \cdot \mathbf{e}_i + \sum_{k=1}^{2^n} \nu_k \cdot \mathbf{S}_k = 0, \qquad [24]$$

where $\mathbf{1}_{a,j} = \sum_{i \in \mathcal{S}_{j,a}} \mathbf{e}_i$. Restricting to a single i gives the following minor variant of Eq. (21):

$$2 \cdot R_i^* - \lambda_{a,j} + \mu_{0,i} - \mu_{1,i} + \sum_{k=1}^n \nu_k \cdot S_{k,i} = 0.$$

The proof then proceeds identically; the only difference is to note that while $\lambda_{a,j}$ varies by stratum, $\Delta = \sum_{k=1}^{2^n} \nu_k$ does not. ☐

It follows that solving the minimization problem across all strata can be carried out in almost the same way as across a single stratum:

1. Construct piecewise linear functions $\epsilon_{a,j}(\Delta)$ and piecewise quadratic functions $\Sigma_{a,j}(\Delta)$ for each stratum;
2. Note that because the sums $\sum_{a=0}^1 \sum_{j=1}^m \epsilon_{a,j}(\Delta)$ and $\sum_{a=0}^1 \sum_{j=1}^m \Sigma_{a,j}(\Delta)$ are also piecewise linear and quadratic, respectively, they can also be evaluated using Algorithm 2;
3. Find Δ^* such that $\sum_{a=0}^1 \sum_{j=1}^m \epsilon_{a,j}(\Delta^*) = \epsilon$, and evaluate $\sum_{a=0}^1 \sum_{j=1}^m \Sigma_{a,j}(\Delta^*)$.

Consequently, beyond the machinery established in Algorithms 2, 3, and 4, we only need a way of calculating the sums of these piecewise functions. This can be carried out straightforwardly using a variation on the standard merge-sort algorithm, which we give in Algorithm 5 for completeness.

Putting this all together, we obtain the following lemma summarizing the results of this section.

Lemma S10. *If $\boldsymbol{\ell}$, $\hat{\mathbf{R}}$, and $\boldsymbol{u}$ have been sorted, then there exists an $O(\log(m) \cdot n)$ algorithm solving the simplified minimization problem (*Eq. (12)*).*

Proof. The proof is straightforward. We begin by noting that Algorithm 2 requires linear time: the index i increases by one in the while loop on lines 6 through 10 on each iteration, and must be less than the length of the input. Similarly, in Algorithm 3, the while loop on lines 11 through 22 increments i^{lwr} by one on each loop, and limits it to three times the length of the input. The same analysis applies to Algorithm 4, in which either i^{upr} or i^{lwr} increases by one on each iteration of the loop on lines 14 through 33. We note that the run time of Algorithm 5 is proportional to the sum of the lengths of its inputs. Using a standard divide-and-conquer approach, we can accomplish the combination of the $2m$ strata in m total calls, where each element of the input appears in $1 + \lceil \log_2(m) \rceil$ of the function calls, for a total time complexity of $O(\log(m) \cdot n)$. Putting this all together, the total complexity is $O(\log(m) \cdot n)$. ☐

2.B.3. Maximization. Even in the simplified problem in Eq. (12), maximization is not a convex problem. In general, maximizing a quadratic objective over a linearly constrained convex set is NP-hard (24). However, the restricted forms of the objective and constraints allow us efficiently to solve the optimization problem exactly in the case of a single stratum and approximately in the case of multiple strata.

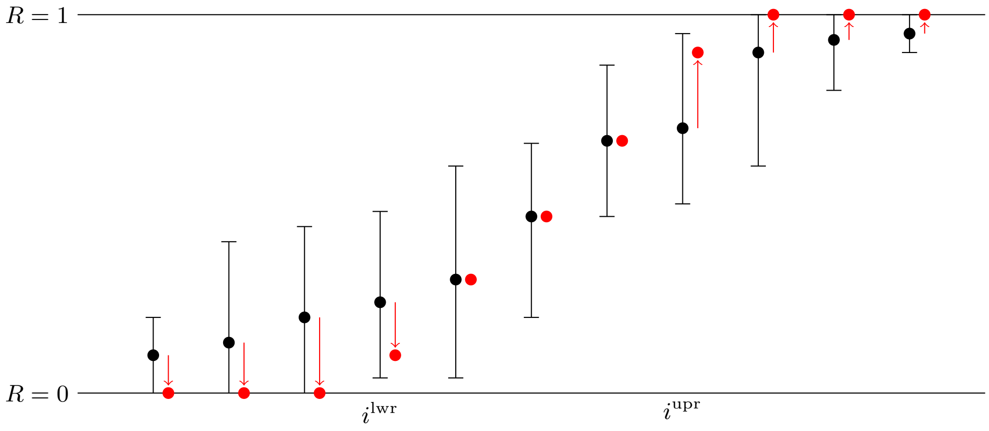

Fig. S2. *Maximization normal form. Black dots represent estimated risks $\hat{R}_i$, the error bars represent the allowable range $[\ell_i, u_i]$, and red dots indicate the corresponding value of the normal form vector R_i. The pivots are indicated on the x-axis.*

Optimizing over a single stratum As in the case of minimization, risk vectors that maximize the single-stratum maximization problem have a special normal form.

Definition S11 (Maximization normal form). We say that a risk vector $\mathbf{R}$ is in *maximization normal form* if there exist indices ("pivots") i^{lwr} and i^{upr} such that

$$R_i = \begin{cases} \ell_i & i < i^{\text{lwr}}, \\ \hat{R}_i & i^{\text{lwr}} < i < i^{\text{upr}}, \\ u_i & i^{\text{upr}} < i. \end{cases}$$

We additionally require that the risk values at the pivots themselves satisfy $R_{i^{\text{lwr}}} \in [\ell_{i^{\text{lwr}}}, \hat{R}_{i^{\text{lwr}}}]$ and $R_{i^{\text{upr}}} \in [\hat{R}_{i^{\text{upr}}}, u_{i^{\text{upr}}}]$.[13,14]

Maximization normal form is similar to minimization normal form, except that instead of being pushed toward the thresholds, values at indices *below* the lower pivot are pushed down, and values at indices *above* the upper pivot are pushed up. An illustration of maximum normal form is given in Figure S2.

An important difference from the case of minimization is that studying the KKT conditions does not yield the normal form directly; instead, it yields a weaker characterization.

Definition S12 (Weak normal form). We say that a risk vector is in *weak normal form* if there exist thresholds t^{lwr} and t^{upr} such that

$$R_i = \begin{cases} \ell_i & R_i < t^{\text{lwr}}, \\ t^{\text{lwr}} & R_i = t^{\text{lwr}}, \\ \hat{R}_i & t^{\text{lwr}} < R_i < t^{\text{upr}}, \\ t^{\text{upr}} & R_i = t^{\text{upr}}, \\ u_i & t^{\text{upr}} < R_i, \end{cases}$$

and there exists at most one lower index i^{lwr} such that $R_{i^{\text{lwr}}} = t^{\text{lwr}}$; and similarly, there exists at most one upper index i^{upr} such that $R_{i^{\text{upr}}} = t^{\text{upr}}$

We note that, in the case of *weak* maximization normal form, the indices i^{lwr} and i^{upr} are analogous to the pivots in maximization normal form; however, they do not share the key property, which is that all of the indices $i < i^{\text{lwr}}$ index risks that have been pushed down all the way to ℓ_i and all of the indices $i > i^{\text{upr}}$ index risks that have been pushed all the way up to u_i; see Figure S3.

Lemma S13. *Consider the single-stratum maximization problem—i.e., maximization in* Eq. (18). *Suppose that $\mathbf{R}^*$ is a solution. Then, $\mathbf{R}^*$ is in weak normal form. Moreover, either $\frac{1}{n}\|\mathbf{R}^* - \hat{\mathbf{R}}\|_1 = \epsilon$ or R_i^* equals ℓ_i or u_i for all but (at most) one i.*

Proof. The proof is quite similar to the proof of Lemma S8. We note that the first-order KKT conditions are almost the same, namely that

$$2 \cdot \mathbf{R}^* - \lambda \cdot \mathbf{1} - \sum_{i=1}^{n} \mu_{0,i} \cdot \mathbf{e}_i + \sum_{i=1}^{n} \mu_{1,i} \cdot \mathbf{e}_i - \sum_{k=1}^{2^n} \nu_k \cdot \mathbf{S}_k = 0 \qquad [25]$$

[13] Since it involves the ordering of the indices as well as the risk values, the appropriate generalization of maximization normal form is notationally awkward to express when multiple strata are involved. Fortunately, in contrast to the case of minimization, we will not have occasion to use such a generalization, and so do not give its definition.

[14] We note that we allow i^{lwr} and i^{upr} to take the values 0 or $n+1$ in addition to $\{1,\ldots,n\}$ to cover cases where, e.g., $R_i = \ell_i$ for all $i = 1,\ldots,n$.

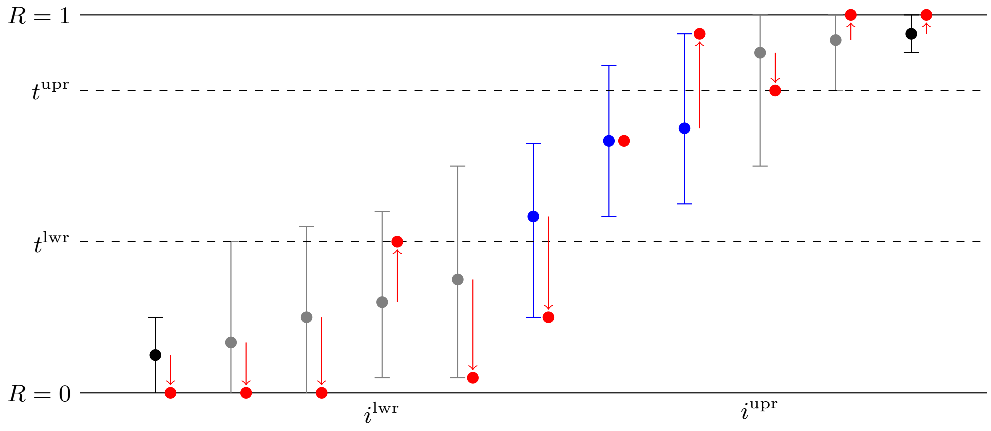

Fig. S3. *Weak maximization normal form. Black, blue, and grey dots represent estimated risks $\hat{R}_i$, the error bars represent the allowable range $[\ell_i, u_i]$, and red dots indicate the corresponding value of the normal form vector R_i. The pivots are indicated on the x-axis. The risk R_i at indices where the estimated risk is between the thresholds and the bounds straddle the thresholds (shown in blue) can take on a number of values (e.g., $R_i = \hat{R}_i$ or $R_i = u_i$). Likewise, i^{lwr} and i^{upr} can occur at any index i such that $R_i = t^{\mathrm{lwr}}$ or $R_i = t^{\mathrm{upr}}$ is feasible (shown in grey). As a result, the set of risk vectors in* weak *maximization normal form is a very complex search space.*

for some λ and $\mu_{0,i}, \mu_{1,i}, \nu_k \geq 0$ satisfying complementary slackness. Virtually exactly as before, it follows from this that Eq. (20) holds, i.e., that

$$R_i^* \in \left\{ \hat{R}_i, u_i, \ell_i, \frac{1}{2} \cdot [\lambda - \Delta], \frac{1}{2} \cdot [\lambda + \Delta] \right\}. \qquad [26]$$

Again, as in the case of minimization, we can strengthen Eq. (26). In particular, exactly the same argument used to strengthen the weak conditions in Lemma S8 yields that if $R_{i_0}^* > \hat{R}_{i_0}$ and $R_{i_0}^* < R_{i_1}^*$, then $R_{i_1}^* = u_{i_1}$; and, conversely, if $R_{i_0}^* < \hat{R}_{i_0}$ and $R_{i_0}^* > R_{i_1}^*$, then $R_{i_1}^* = \ell_{i_1}$. The proof is then otherwise the same as in Lemma S8.

To see that there can be at most one i such that such that $\ell_i < R_i^* < \hat{R}_i$, suppose that $\ell_{i_0} < R_{i_0}^* < \hat{R}_{i_0}$ and $\ell_{i_1} < R_{i_1}^* < \hat{R}_{i_1}$ for $i_0 \neq i_1$. By Eq. (26), $R_{i_0}^* = R_{i_1}^*$. Choose $\delta > 0$ sufficiently small that $R_{i_0}^* - \delta > \ell_{i_0}$ and $R_{i_1}^* + \delta < \hat{R}_{i_1}$. Then, as in the case of minimization, it follows immediately that $\mathbf{R}^* + \delta(\mathbf{e}_{i_1} - \mathbf{e}_{i_0})$ is feasible, but increases the objective by $2\delta^2$, contrary to the hypothesis that $\mathbf{R}^*$ was a maximizer. In the same way, it follows that there exists at most one i' such that $u_{i'} > R_{i'}^* > \hat{R}_{i'}$.

Combining this with our previous necessary condition on $\mathbf{R}^*$ and letting $t^{\mathrm{lwr}} = \frac{1}{2} \cdot [\lambda - \Delta]$ and $t^{\mathrm{upr}} = \frac{1}{2} \cdot [\lambda + \Delta]$ yields that for all i such that $R_i^* < t^{\mathrm{lwr}}$, $R_i^* = \ell_i$; that for all i such that $R_i^* > t^{\mathrm{upr}}$, $R_i^* = u_i$; that there is at most one index such that $\hat{R}_i > R_i^* > \ell_i$; and, finally, that there is at most one i' such that $\hat{R}_{i'} < R_{i'}^* < u_{i'}$. □

Lemma S13 is, by itself, insufficient to form the basis of an effective solution algorithm since it does not determine which indices are i^{lwr} and i^{upr}. Even if these indices were known, they do not pin down the value of R_i for $i \neq i^{\mathrm{lwr}}, i^{\mathrm{upr}}$, which could, almost without restriction, be ℓ_i, $\hat{R}_i$, or u_i. As a result, the search space of vectors in weak maximization normal form is extremely large.

Therefore, to construct an effective maximization algorithm, we must transform vectors merely in weak maximization normal form into vectors that are in full-fledged maximization normal form.

To do so, we will have need of the following elementary fact.

Lemma S14. *Suppose $a \geq b$ and $c \geq d$. Then*

$$|a - c| + |b - d| \leq |a - d| + |b - c|.$$

Proof. The proof is not complicated, but is simplest to understand if expressed in geometric terms. Consider the points $\mathbf{p}_0 = (a, b)$ and $\mathbf{p}_1 = (c, d)$. Then both $\mathbf{p}_0$ and $\mathbf{p}_1$ lie in the lower half-plane

$$H = \{ (x, y) : x \geq y \}.$$

Let $T : (x, y) \mapsto (y, x)$ be the linear transformation given by reflection across the line $y = x$. Then, the claim of the lemma can be reframed as follows: for any $\mathbf{p}_0, \mathbf{p}_1 \in H$,

$$\|\mathbf{p}_0 - \mathbf{p}_1\|_1 \leq \|\mathbf{p}_0 - T(\mathbf{p}_1)\|_1. \qquad [27]$$

This setup is shown in Figure S4a. We divide into four cases according to whether $a \geq c$ and $b \geq d$, or, equivalently, depending on in which of the four regions shown in Figure S4b the point $\mathbf{p}_1$ lies.

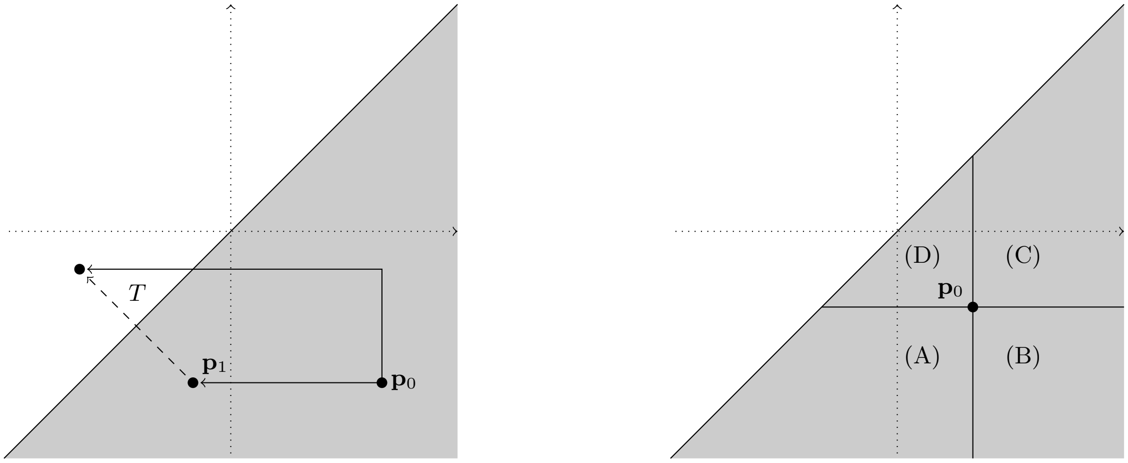

(a) Setup

(b) Division into cases

Fig. S4. *An illustration of the proof of Lemma S14. The half-plane H is shown in gray, with the dotted lines representing the coordinate axes.* Left: *The claim of the lemma is equivalent to the claim that the path joining $\mathbf{p}_0$ to $\mathbf{p}_1$ is no longer than the path joining $\mathbf{p}_0$ to $T(\mathbf{p}_1)$.* Right: *The division into four cases, depending on where $\mathbf{p}_0$ lies relative to $\mathbf{p}_1$.*

Case (A): $a \geq c$ **and** $b \geq d$ In this case, we have that

$$\begin{aligned} |a - c| + |b - d| &= a - c + b - d \\ &= a - d + b - c \\ &\leq |(a - d) + (b - c)| \\ &\leq |a - d| + |b - c|. \end{aligned}$$

Therefore, Eq. (27) holds.

Case (B): $a \leq c$ **and** $b \geq d$ In this case,

$$\begin{aligned} |a - c| + |b - d| &= c - a + b - d \\ &\leq c - a + b - d + 2(a - b) \\ &= c - b + a - d \\ &\leq |(c - b) + (a - d)| \\ &\leq |c - b| + |a - d|, \end{aligned}$$

so Eq. (27) holds in this case as well.

Before completing the proof, we note that T has the useful property that

$$\|T(\mathbf{p})\|_1 = \|\mathbf{p}\|_1. \qquad [28]$$

Case (C): $a \leq c$ **and** $b \leq d$ We note that this case is the same as Case (A) with the roles of $\mathbf{p}_0$ and $\mathbf{p}_1$ reversed. Consequently, by Eq. (27), we have that

$$\|\mathbf{p}_0 - \mathbf{p}_1\|_1 \leq \|T(\mathbf{p}_0) - \mathbf{p}_1\|_1.$$

Applying Eq. (28) and using the fact that T^2 is the identity, we have that

$$\|T(\mathbf{p}_0) - \mathbf{p}_1\|_1 = \|T(T(\mathbf{p}_0) - \mathbf{p}_1)\|_1 = \|\mathbf{p}_0 - T(\mathbf{p}_1)\|_1,$$

which yields Eq. (27).

Case (D): $a \geq c$ **and** $b \leq d$ We argue as in the previous case, noting that this case is equivalent to Case (B) with the roles of $\mathbf{p}_0$ and $\mathbf{p}_1$ reversed, whence

$$\|\mathbf{p}_0 - \mathbf{p}_1\|_1 \leq \|T(\mathbf{p}_0) - \mathbf{p}_1\|_1 = \|\mathbf{p}_0 - T(\mathbf{p}_1)\|_1.$$

□

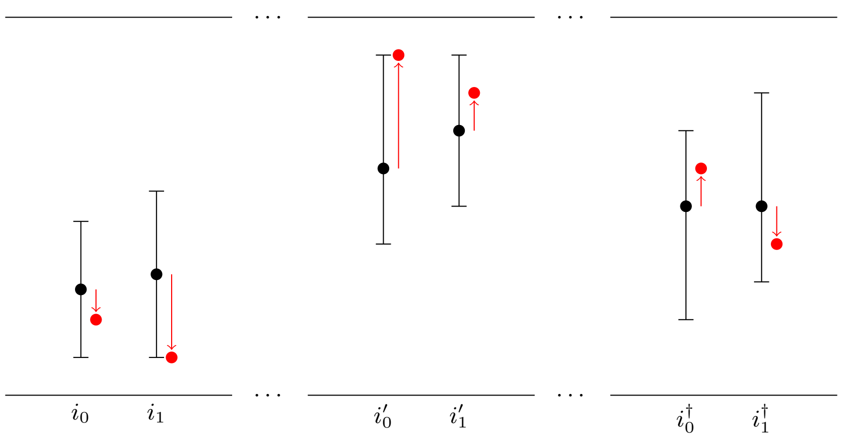

Fig. S5. *An illustration of* trivial deviations *from normality. As in Figures S1 and S2, the black dots represent estimated risks $\hat{R}_i$, the error bars represent the allowable range $[\ell_i, u_i]$, and the red dots represent the corresponding values of R_i, with indices increasing from left to right on the x-axis. There is a trivial deviation from normality relative to the lower bound on the left because $\ell_{i_0} = \ell_{i_1}$ but $R_{i_1} = \ell_{i_1}$, and a similar illustration of a trivial deviation from normality relative to the upper bound in the center. On the right there is a trivial deviation from the estimates.*

A further difference from minimization is that the additional assumption of sortability, Definition S1, is required to connect maximizers to maximization normal form. If sortability fails, there may be intervals $[\ell_i, u_i]$ that can be non-trivially nested for distinct i, which can cause i_0 and i_1 to "jump between" different indices a potentially exponential number of times in the search for a maximizer. For notational convenience, we assume throughout that sortable constraints are, in fact, already sorted.

The first step to deriving an effective maximization algorithm is to remove "trivial" deviations from maximization normal form.

Definition S15 (Trivial deviations from normality). We say that a risk vector in weak normal form has a *trivial deviation from normality* relative to its *bounds* if there are $i_0 < i_1$ such that $\ell_{i_0} = \ell_{i_1}$, $\ell_{i_0} < R_{i_0}$, and $R_{i_1} = \ell_{i_1}$; or there are $i'_1 > i'_0$ such that $u_{i'_0} = u_{i'_1}$, $R_{i'_1} < u_{i'_1}$, and $R_{i'_0} = u_{i'_0}$.

We say that a risk vector in weak normal form has a *trivial deviation from normality* relative to its *estimates* if there are $i_0 < i_1$ such that $\hat{R}_{i_0} = \hat{R}_{i_1}$ but $R_{i_0} > R_{i_1}$.

An illustration of trivial deviations from normality is shown in Figure S5. As their name suggests, trivial deviations from normality are straightforward to eliminate.

Lemma S16. *Suppose the constraints are sortable. If a feasible risk vector* $\mathbf{R}$ *is in weak normal form with trivial deviations from normality, then there exists a feasible* $\mathbf{R}'$ *in weak normal form with no trivial deviations from normality such that*

$$\frac{1}{n}\sum_{i=1}^{n} R_i^2 = \frac{1}{n}\sum_{i=1}^{n} (R'_i)^2.$$

Proof. We first define an algorithm for removing trivial deviations from normality, and then show that it must eventually terminate when all trivial deviations have been removed.

Algorithm If there were any indices i_0 and i_1 representing a trivial deviation from normality, then we could eliminate them by swapping R_{i_0} and R_{i_1}, i.e., by considering

$$R'_i = R_i \quad (i \neq i_0, i_1), \qquad R'_{i_0} = R_{i_1}, \qquad R'_{i_1} = R_{i_0}.$$

In particular, it follows immediately that $\mathbf{R}'$ is still in weak normal form and that $\frac{1}{n}\sum_{i=1}^{n} R_i^2 = \frac{1}{n}\sum_{i=1}^{n}(R'_i)^2$. Because the constraints sorted, the only feasibility constraint on $\mathbf{R}'$ that is not satisfied trivially is that $\|\mathbf{R}' - \hat{\mathbf{R}}\|_1 \leq \epsilon$. However, this constraint follows by applying Lemma S14 with $a = R_{i_0}, b = R_{i_1}, c = \hat{R}_{i_1}$, and $d = \hat{R}_{i_0}$, which yields

$$\|\mathbf{R}' - \hat{\mathbf{R}}\|_1 \leq \|\mathbf{R} - \hat{\mathbf{R}}\|_1 \leq \epsilon.$$

Termination We call a pair of indices (i_0, i_1) *out of order* if $i_0 < i_1$, but $R_{i_0} > R_{i_1}$. We note that any trivial deviation from normality requires that the corresponding indices i_0 and i_1 are out of order. Then, we observe the following facts:

Fact 1. There are only finitely many pairs of out-of-order indices.

Fact 2. As long as there is a trivial deviation of any kind, it is possible to swap indices so as to remove that trivial deviation.

Fact 3. Any swap undertaken as part of the algorithm strictly reduces the number of out-of-order pairs of indices.

We note that it follows immediately from these three facts that a greedy algorithm removing trivial deviations in any order whatsoever must eventually terminate.

Fact 1 is immediate, and Fact 2 follows from the discussion of the algorithm above. To see that Fact 3 holds, it suffices to show that *any* swap of out-of-order pairs of indices strictly reduces the number of out-of-order pairs of indices.

To this end, suppose $i_0 < i_1$ are out of order. As before, we use $\mathbf{R}'$ to denote the risk vector *after* swapping indices i_0 and i_1. First, suppose there is an index i^* such that $i_0 < i^* < i_1$. Then, if (i_0, i^*) is out of order *after* swapping i_0 and i_1, it must have been out of order before swapping, since

$$R_{i_0} > R_{i_1} = R'_{i_0} > R'_{i^*} = R_{i^*}.$$

Likewise, if (i^*, i_1) is out of order *after* swapping i_0 and i_1, it too must have been out of order before swapping, since

$$R_{i^*} = R'_{i^*} > R'_{i_1} = R_{i_0} > R_{i_1}.$$

Therefore, swapping cannot increase the number of out-of-order pairs involving $i_0 < i^* < i_1$.

On the other hand, since $R'_{i_0} < R'_{i_1}$, if $i^* < i_0$, then there are three possibilities:

Case 1. After swapping, neither (i^*, i_0) nor (i^*, i_1) is out of order.

Case 2. After swapping, both (i^*, i_0) and (i^*, i_1) are out of order.

Case 3. After swapping, (i^*, i_0) is out of order, but (i^*, i_1) is not.

In the first case, the number of out-of-order pairs cannot have increased, so we only need to consider the second and third cases. In Case 2, we must have that

$$R_{i^*} = R'_{i^*} > R'_{i_1} = R_{i_0} > R_{i_1},$$

so both (i^*, i_0) and (i^*, i_1) were out of order before swapping. In Case 3, we must have that

$$R_{i_0} = R'_{i_1} \geq R'_{i^*} = R_{i^*} > R'_{i_0} = R_{i_1},$$

so that before swapping (i^*, i_1) was out of order, but (i^*, i_0) was not. Therefore, it follows that swapping again cannot increase the number of out-of-order pairs involving $i^* < i_0$. A similar argument shows that the number of out-of-order pairs involving $i_1 < i^*$ does not increase after swapping. Therefore swapping strictly reduces the number of out-of-order pairs, since it at least eliminates the pair (i_0, i_1), which is Fact 3. □

With these preliminaries in hand, we are prepared to solve the maximization problem.

Lemma S17. *Suppose the constraints are sortable. Then the single stratum maximization problem in* Eq. (18) *is maximized by a risk vector in maximization normal form and that either exhausts the budget or has* $i^{\text{lwr}} = i^{\text{upr}}$.

Proof. We adopt a strategy similar to that used in the "strengthening" portion of the proof of Lemma S8. Namely, we show that any point satisfying the necessary conditions of Lemma S13 but without pivots can be "improved" to a feasible point that achieves a greater objective.

Let $\mathbf{R}^*$ be a maximizer. By Lemma S13, $\mathbf{R}^*$ is in weak maximization normal form; by Lemma S16, we may assume that $\mathbf{R}^*$ has no trivial deviations from normality.

To prove the theorem, we first observe that it suffices to prove the following two claims:

1. There do not exist $i_0 < i_1$ such that $R^*_{i_0} > \ell_{i_0}$ and $R^*_{i_1} < \hat{R}_{i_1}$,
2. There do not exist $i_0 < i_1$ such that $R^*_{i_1} < u_{i_1}$ and $R^*_{i_0} > \hat{R}_{i_0}$.

First, we see how the theorem follows from the two claims. Define i^{lwr} and i^{upr} as follows:

$$i^{\text{lwr}} = \min\{\, i : R^*_i > \ell_i \,\}, \qquad i^{\text{upr}} = \max\{\, i : R^*_i < u_i \,\},$$

then, for all $i < i^{\text{lwr}}$, $R^*_i = \ell_i$, and for all $i > i^{\text{upr}}$, $R^*_i = u_i$. Moreover, for all i satisfying $i^{\text{lwr}} < i < i^{\text{upr}}$, by Claim 1, $R^*_i \geq \hat{R}_i$; and, by Claim 2, $R^*_i \leq \hat{R}_i$. Consequently, $R^*_i = \hat{R}_i$ for these indices. Moreover, if $i^{\text{lwr}} \neq i^{\text{upr}}$, by Claim 2, $R^*_{i^{\text{lwr}}} \leq \hat{R}_{i^{\text{lwr}}}$, i.e., $R^*_{i^{\text{lwr}}} \in [\ell_{i^{\text{lwr}}}, \hat{R}_{i^{\text{lwr}}}]$. Similarly, by Claim 1, we conclude that $R^*_{i^{\text{upr}}} \in [\hat{R}_{i^{\text{upr}}}, u_{i^{\text{upr}}}]$.

Therefore it suffices to prove the two claims. Since the proofs are virtually identical, we focus on Claim 1.

The key observation is the following. Suppose there exist indices $R^*_{i_0}$ and $R^*_{i_1}$ with $R^*_{i_1} \geq R^*_{i_0}$, and suppose that there is some δ such that after decreasing $R^*_{i_0}$ and increasing $R^*_{i_1}$ by δ the result is still feasible. Then $\mathbf{R}^*$ cannot be maximal, since, letting $\mathbf{R}' = \mathbf{R}^* + \delta(\mathbf{e}_{i_1} - \mathbf{e}_{i_0})$, we have that

$$\begin{aligned}\left[\sum_{i=1}^{n}(R'_i)^2\right] - \left[\sum_{i=1}^{n}(R^*_i)^2\right] &= (R^*_{i_0} - \delta)^2 + (R^*_{i_1} + \delta)^2 - (R^*_{i_0})^2 - (R^*_{i_1})^2 \\ &= 2\delta(\delta + [R^*_{i_1} - R^*_{i_0}]) \\ &\geq 2\delta^2 \\ &> 0.\end{aligned} \qquad [29]$$

To actually prove the claim, we proceed by contradiction. In particular, suppose that $i_0 < i_1$ represented an exception to Claim 1, so that $R^*_{i_0} > \ell_{i_0}$ and $R^*_{i_1} < \hat{R}_{i_1}$.

If $R^*_{i_0} \leq R^*_{i_1}$, then Eq. (29) immediately applies, since increasing $R^*_{i_1}$ *decreases* the L_1 distance between $\hat{\mathbf{R}}$ and $\mathbf{R}^*$ and decreasing $R^*_{i_0}$, at worst, *increases* it at the same rate. More formally, for $0 < \delta$ sufficiently small,

$$\begin{aligned}|(R^*_{i_0} - \delta) - \hat{R}_{i_0}| + |(R^*_{i_1} + \delta) - \hat{R}_{i_1}| &= |(R^*_{i_0} - \delta) - \hat{R}_{i_0}| + \hat{R}_{i_1} - (R^*_{i_1} + \delta) \\ &\leq |R^*_{i_0} - \hat{R}_{i_0}| + \delta + \hat{R}_{i_1} - R^*_{i_1} - \delta \\ &= |R^*_{i_0} - \hat{R}_{i_0}| + |R^*_{i_1} - \hat{R}_{i_1}|,\end{aligned}$$

and so $\mathbf{R}'$ is feasible. Because $R^*_{i_0} \leq R^*_{i_1}$, by Eq. (29), $\mathbf{R}'$ achieves a greater average sum of squares that $\mathbf{R}^*$, contrary to the assumed maximality of $\mathbf{R}^*$.

On the other hand, if $R^*_{i_0} > R^*_{i_1}$, then, by Lemma S14, the vector $\mathbf{R}^\dagger$ given by switching the indices i_0 and i_1, i.e.,

$$R^\dagger_i = R^*_i \quad (i \neq i_0, i_1), \qquad R^\dagger_{i_0} = R^*_{i_1}, \qquad R^\dagger_{i_1} = R^*_{i_0},$$

satisfies $\|\mathbf{R}^\dagger - \hat{\mathbf{R}}\|_1 \leq \epsilon$. Moreover, applying our sortability hypothesis, we have that

$$u_{i_0} \geq R^*_{i_0} > R^*_{i_1} \geq \ell_{i_1} \geq \ell_{i_0},$$

and

$$u_{i_1} \geq u_{i_0} \geq R^*_{i_0} > R^*_{i_1} \geq \ell_{i_1},$$

whence for all $i = 1, \ldots, n$, $\ell_i \leq R^\dagger_i \leq u_i$. Therefore $\mathbf{R}^\dagger$ is feasible. However, since $\mathbf{R}^*$ has no trivial deviations from normality, it follows that

$$R^\dagger_{i_0} = R^*_{i_1} \geq \ell_{i_1} > \ell_{i_0}, \qquad R^\dagger_{i_1} = R^*_{i_0} \leq u_{i_0} < u_{i_1}.$$

Since $R^\dagger_{i_0} < R^\dagger_{i_1}$, we would like to apply Eq. (29) to derive a contradiction. To that end, we require that $\mathbf{R}^\dagger + \delta(\mathbf{e}_{i_1} - \mathbf{e}_{i_0})$ is feasible for sufficiently small $\delta > 0$.

To see that such δ exists, we divide into three cases. Observe that one of the following three conditions must hold: (1) $R^*_{i_0} < \hat{R}_{i_1}$, (2) $R^*_{i_1} > \hat{R}_{i_0}$, or (3) $R^*_{i_0} \geq \hat{R}_{i_1}$ and $R^*_{i_1} \leq \hat{R}_{i_0}$. We treat each case separately.

Case 1 ($R^*_{i_0} < \hat{R}_{i_1}$)**.** Note that if it were the case that

$$\|\mathbf{R}^\dagger + \delta(\mathbf{e}_{i_1} - \mathbf{e}_{i_0}) - \hat{\mathbf{R}}\|_1 \leq \|\mathbf{R}^\dagger - \hat{\mathbf{R}}\|_1, \tag{30}$$

then the result follows immediately. Choosing $\delta < \hat{R}_{i_1} - R^*_{i_0}$, we have, using the fact that $R^\dagger_{i_0} = R^*_{i_1}$ and $R^\dagger_{i_1} = R^*_{i_0}$ that

$$|R^\dagger_{i_0} - \delta - \hat{R}_{i_0}| + |R^\dagger_{i_1} + \delta - \hat{R}_{i_1}| = |R^\dagger_{i_0} - \delta - \hat{R}_{i_0}| + \hat{R}_{i_1} - R^\dagger_{i_1} - \delta \geq |R^\dagger_{i_0} - \hat{R}_{i_0}| + \delta + \hat{R}_{i_1} - R^\dagger_{i_1} - \delta.$$

The final expression is $|R^\dagger_{i_0} - \hat{R}_{i_0}| + |\hat{R}_{i_1} - R^\dagger_{i_1}|$, whence Eq. (30) holds.

Case 2 ($R^*_{i_1} > \hat{R}_{i_0}$)**.** This case is essentially identical to the previous case.

Case 3 ($R^*_{i_0} \geq \hat{R}_{i_1}$ and $R^*_{i_1} \leq \hat{R}_{i_0}$)**.** In this case, we have by sortability that

$$\begin{aligned}|R^*_{i_0} - \hat{R}_{i_0}| + |R^*_{i_1} - \hat{R}_{i_1}| &= R^*_{i_0} - \hat{R}_{i_0} + \hat{R}_{i_1} - R^*_{i_1} \\ &> (R^*_{i_0} - \hat{R}_{i_0} + \hat{R}_{i_1} - R^*_{i_1}) + 2(\hat{R}_{i_0} - \hat{R}_{i_1}) \\ &= R^*_{i_0} - \hat{R}_{i_1} + \hat{R}_{i_0} - R^*_{i_1} \\ &= |R^\dagger_{i_0} - \hat{R}_{i_0}| + |R^\dagger_{i_1} - \hat{R}_{i_1}|,\end{aligned}$$

where we have used the fact that, because there are no trivial deviations from normality relative to the estimates, $\hat{R}_{i_1} > \hat{R}_{i_0}$. Therefore $\|\mathbf{R}^\dagger - \hat{\mathbf{R}}\|_1 < \epsilon$ and so we can choose δ sufficiently small that $\|\mathbf{R}^\dagger + \delta(\mathbf{e}_{i_1} - \mathbf{e}_{i_0}) - \hat{\mathbf{R}}\|_1 \leq \epsilon$.

Therefore, no such $i_0 < i_1$ exist, and so $\mathbf{R}^*$ is in maximization normal form. □

Lemma S17 allows us to efficiently search over the space of risk vectors to find the maximizer. In particular, all that is necessary is to decrease the risk at the lower pivot and increase it at the upper pivot until the L_1 budget is expended. The linear-time algorithm for doing so is given in Algorithm 7. (As in the case of Algorithm 4, there is a potential preprocessing step, given in Algorithm 6.) As we did in the minimization algorithm, we capture the maximization algorithm's value at a variety of L_1 budgets, controlled by some step-size γ—which, we assume, satisfies $k\gamma = \epsilon$ for some $k \in \mathbb{N}$—rather than just the full budget ϵ. We denote this collection with $\mathbf{\Sigma}$. The reason for doing this is to allow us to maximize across strata.

Optimizing over all strata Consider the simplified maximization problem in Eq. (12). Since the objective is separable, the problem would be separable in the different strata, except that the L_1 budget constraint ranges over all of the strata simultaneously. Much as we reduced the base problem to the parameterized problem by introducing additional parameters τ_j, $j = 1, \ldots, m$, if the optimal budget allocation to each stratum were known, then we could reduce to the single stratum case, applying Algorithm 7. In particular, we note that the following optimization problem (hereafter **"the separable problem"**) parameterized by the stratum-specific budgets $\epsilon_{1,0}, \ldots, \epsilon_{m,0}, \epsilon_{1,1}, \ldots, \epsilon_{m,1}$ is separable:

$$\begin{aligned}
\underset{\mathbf{R}\in\mathbb{R}^n}{\text{Optimize}} \quad & \frac{1}{n}\sum_{i=1}^{n} R_i^2 \\
\text{s.t. } & \tfrac{1}{n}\textstyle\sum_{i\in\mathcal{S}_{j,a}}^{n} |R_i - \hat{R}_i| \le \epsilon_{j,a}, \; (a = 0, 1;\; j = 1, \ldots, m) \\
& \textstyle\sum_{i\in\mathcal{S}_{j,1}} R_i = \rho_j, \qquad (j = 1, \ldots, m) \\
& \textstyle\sum_{i\in\mathcal{S}_{j,0}} R_i = \tau_j, \qquad (j = 1, \ldots, m) \\
& R_i \le u_i, \qquad (i = 1, \ldots, n) \\
& R_i \ge \ell_i. \qquad (i = 1, \ldots, n)
\end{aligned} \tag{31}$$

In particular, the separable problem can be solved stratum by stratum, since the constraints and objective are both separable at the stratum level.

Reducing solving the simplified problem to solving the separable problem, however, is complex: There is no obvious way to determine the optimal L_1 budget allocation other than by sweeping over all possible budgets. However, it is nevertheless possible to efficiently discover an *approximately* optimal budget allocation using a divide-and-conquer approach. In particular, as shown in Algorithm 7, it is only marginally more difficult computationally to output the maximum value of the objective for the entire budget than to output the maximum value of the objective for a range of budgets, i.e., to output $\mathbf{\Sigma}$, which gives the maximum value of the objective for the budgets 0, γ, 2γ, ..., ϵ.

Given $\mathbf{\Sigma}^{(0)}$ and $\mathbf{\Sigma}^{(1)}$ corresponding to two different strata when the allocated budget is 0, γ, 2γ, ..., ϵ, we can find the approximate maximum value of the objective when the *total* budget allocated to both strata (in increments of γ) is 0, γ, 2γ, ..., ϵ by sweeping over the two dimensional grid of points $\{0, \gamma, \ldots, \epsilon\} \times \{0, \gamma, \ldots, \epsilon\}$, and, for $k\gamma$, outputting

$$\max\left(\Sigma_0^{(0)} + \Sigma_{k+1}^{(1)}, \Sigma_1^{(0)} + \Sigma_k^{(1)}, \ldots, \Sigma_{k+1}^{(0)} + \Sigma_0^{(1)}\right).$$

For completeness, we explicitly give this maximization routine in Algorithm 8.

Lemma S18. *If $\boldsymbol{\ell}$, $\hat{\mathbf{R}}$ and $\mathbf{u}$ have been sorted, then the approximate solution to the maximization problem in* Eq. (12) *given by applying Algorithms 7 and 8 is*

$$O\left(m \cdot \left(\frac{\epsilon}{\gamma}\right)^2 + n\right).$$

Proof. We begin by analyzing Algorithm 6. We note that the number of iterations of the while loop on lines 7 through 12 is capped by the length of the input, since i^{upr} is decremented on each input. Likewise, Algorithm 7 is almost linear in the size of the input: In the while loop on lines 14 though 32, either i^{lwr} is incremented, or the subsequent multiple of γ is reached, meaning that the algorithm as a whole is linear in the size of the input and ϵ/γ. Therefore, running Algorithm 7 over all $2m$ strata requires $O(n + \frac{m\epsilon}{\gamma})$ time.

All of the strata can be combined using $2m - 1$ applications of Algorithm 8. Reviewing the for loop on lines 3 through 10 and the while loop on lines 5 through 8, we see that exactly

$$\frac{\frac{\epsilon}{\gamma} \cdot (\frac{\epsilon}{\gamma} + 1)}{2}$$

iterations are performed. Adding this to the previous runtime obtained and simplifying gives the desired expression. □

2.B.4. Controlling approximation error. There are two sources of approximation error in our algorithm. The first is the approximation error introduced by solving the maximization problem only approximately. The second is the approximation error introduced by the fact that we cannot sweep over all possible values of $\boldsymbol{\tau} = (\tau_1, \ldots, \tau_m)$ when solving the simplified problem; instead, we must sweep over a grid. Characterizing how much error is introduced by each of these approximations is our final task.

We begin with the following simple lemma.

Lemma S19. *Suppose $\mathbf{R}^{(0)}$ and $\mathbf{R}^{(1)}$ are risk vectors. Then*

$$\left|\sum_{i=1}^{n}(R_i^{(1)})^2 - \sum_{i=1}^{n}(R_i^{(0)})^2\right| \le 2 \cdot \|\mathbf{R}^{(1)} - \mathbf{R}^{(0)}\|_1. \tag{32}$$

Proof. The claim, which is a version of Hölder's inequality, follows straightforwardly from considering the following difference:

$$\begin{aligned}\left|\sum_{i=1}^{n}(R_i^{(1)})^2-(R_i^{(0)})^2\right| &\leq \sum_{i=1}^{n}|(R_i^{(1)})^2-(R_i^{(0)})^2| \\ &\leq \sum_{i=1}^{n}(R_i^{(1)}+R_i^{(0)})\cdot|R_i^{(1)}-R_i^{(0)}| \\ &\leq \sum_{i=1}^{n}2\cdot|R_i^{(1)}-R_i^{(0)}| \\ &= 2\cdot\|\mathbf{R}^{(1)}-\mathbf{R}^{(0)}\|_1.\end{aligned}$$

The first inequality follows from the triangle inequality, and the second and third from the fact that $0 \leq R_i^{(0)}, R_i^{(1)} \leq 1$. □

Using Lemma S19, we obtain the following characterization of the potential error in our maximization algorithm.

Lemma S20. *The difference between the true maximum of the simplified problem—i.e.,* Eq. (12)*—and the quantity obtained from applying Algorithms 7 and 8 is at most* $2m\gamma$.

Proof. Let $\mathbf{R}^*$ be the true maximizing solution. We will show that Algorithm 8 computes the value of the objective at a "close" point at which we can apply Lemma S19.

For each stratum $\mathcal{S}_{j,a}$, let $\epsilon_{j,a}$ denote

$$\frac{1}{n}\sum_{i\in\mathcal{S}_{j,a}}|R_i^*-\hat{R}_i|.$$

Then, there exist $\tilde{\epsilon}_{j,a} \in \{0,\gamma,2\gamma,\ldots,\epsilon\}$ such that $\tilde{\epsilon}_{j,a} < \epsilon_{j,a}$ and $\epsilon_{j,a}-\tilde{\epsilon}_{j,a}$ is less than γ.

Let

$$\Delta = \sum_{a=0}^{1}\sum_{j=1}^{m}\epsilon_{j,a}-\tilde{\epsilon}_{j,a} = \epsilon - k\gamma$$

for some k. Since ϵ is a multiple of γ, it follows that $\Delta = k'\gamma$ for some $k \in \mathbb{N}$, where $k' < 2m$, the number of strata.

Set $\epsilon'_{j,a} = \tilde{\epsilon}_{j,a}$ except for k' arbitrarily chosen strata, where we set $\epsilon'_{j,a} = \tilde{\epsilon}_{j,a}+\gamma$ instead. Then, there exists $\mathbf{R}$ that is in (maximization) normal form such that

$$\frac{1}{n}\sum_{i=1}^{n}|R_i-\hat{R}_i| = \epsilon, \qquad \frac{1}{n}\sum_{i\in\mathcal{S}_{j,a}}|R_i-\hat{R}_i| = \epsilon'_{j,a}.$$

The remainder of the proof follows simply by comparing $\mathbf{R}$ and $\mathbf{R}^*$ stratum by stratum. In particular, consider the restrictions of $\mathbf{R}$ and $\mathbf{R}^*$ to a single stratum $\mathcal{S}_{j,a}$. By Eq. (12), we have that

$$\sum_{i\in\mathcal{S}_{j,a}}R_i = \sum_{i\in\mathcal{S}_{j,a}}R_i^*,$$

and, moreover, by Lemma S17, both $\mathbf{R}_{j,a}$ and $\mathbf{R}^*_{j,a}$ can be assumed to be in maximization normal form. Assume, without loss of generality, that $\epsilon'_{j,a} \leq \epsilon_{j,a}$. (The proof is virtually identical if the inequality is reversed.)

Let i_0 and i_1 denote the pivots of $\mathbf{R}$, and i_0^* and i_1^* of $\mathbf{R}^*$, so that $i_0 \leq i_0^* \leq i_1^* \leq i_1$. (To avoid unnecessary notational complication, we write as if all n individuals belong to the stratum $\mathcal{S}_{j,a}$ and the constraints were sorted.) Then, we note that

$$R_i \geq R_i^* \quad (i_0 \leq i \leq i_0^*), \qquad R_i \leq R_i^* \quad (i_1^* \leq i \leq i_1), \qquad R_i = R_i^* \quad (\text{otherwise}). \tag{33}$$

It follows that

$$\sum_{i=1}^{n}(R_i^*)^2 - R_i^2 = \left[\sum_{i=i_1^*}^{i_1}(R_i^*)^2-R_i^2\right] - \left[\sum_{i=i_0}^{i_0^*}R_i^2-(R_i^*)^2\right]. \tag{34}$$

Both terms in Eq. (34) are positive by Eq. (33). Moreover, by Lemma S19, both terms are less than or equal to

$$2\cdot\left[\sum_{i=i_1}^{i_1^*}R_i^*-R_i\right] = 2\cdot\left[\sum_{i=i_0}^{i_0^*}R_i-R_i^*\right] = 2n\cdot\frac{\epsilon_{a,j}-\epsilon'_{a,j}}{2} \leq n\gamma.$$

In particular, their difference must also be less than this quantity, and so, summing across strata, we have that

$$\sum_{i=1}^{n}(R_i^*)^2 - R_i^2 \leq 2nm\gamma.$$

Dividing through by n gives the result. □

The second element we need is a bound on the sensitivity of the objective of the simplified problem to changes in the parameters. (We recall Remark S3 on notation above.)

Lemma S21. *Let* $\mathbf{R}^{(0)}$ *and* $\mathbf{R}^{(1)}$ *be solutions to the simplified problem in* Eq. (12) *with parameters*

$$\boldsymbol{\tau}^{(0)} = (\tau_1^{(0)}, \dots, \tau_m^{(0)}), \qquad \boldsymbol{\tau}^{(1)} = (\tau_1^{(1)}, \dots, \tau_m^{(1)}),$$

respectively. Then the difference between the objective values of the two solutions is at most $4 \cdot \|\boldsymbol{\tau}^{(0)} - \boldsymbol{\tau}^{(1)}\|_1$.

Proof. The strategy is simple: we will transform $\mathbf{R}^{(0)}$ into a feasible solution of the simplified problem with parameters $\boldsymbol{\tau}^{(1)}$ and *vice versa* for $\mathbf{R}^{(1)}$ and $\boldsymbol{\tau}^{(0)}$. These new solutions lower or upper bound—depending on whether we are considering minimization or maximization—the objective of the new problem; however, by Lemma S19, we can also bound their difference in objective value from the original solutions, giving a bound on the difference in objective value of the original solutions.

In particular, we see that the lemma immediately follows from Lemma S19 and if there exist $\mathbf{R}^{(2)}$ and $\mathbf{R}^{(3)}$ such that

$$\|\mathbf{R}^{(0)} - \mathbf{R}^{(2)}\|_1, \|\mathbf{R}^{(1)} - \mathbf{R}^{(3)}\|_1 \leq 2 \cdot \|\boldsymbol{\tau}^{(0)} - \boldsymbol{\tau}^{(1)}\|_1$$

and such that $\mathbf{R}^{(2)}$ and $\mathbf{R}^{(3)}$ are feasible for the simplified problem with parameters $\boldsymbol{\tau}^{(1)}$ and $\boldsymbol{\tau}^{(0)}$, respectively. For, in that case, we have that, assuming without loss of generality that we are solving the minimization problem,

$$\sum_{i=1}^n (R_i^{(0)})^2 \leq \sum_{i=1}^n (R_i^{(3)})^2 \leq \sum_{i=1}^n (R_i^{(1)})^2 + 4 \cdot \|\boldsymbol{\tau}^{(0)} - \boldsymbol{\tau}^{(1)}\|_1,$$

and similarly

$$\sum_{i=1}^n (R_i^{(1)})^2 \leq \sum_{i=1}^n (R_i^{(2)})^2 \leq \sum_{i=1}^n (R_i^{(0)})^2 + 4 \cdot \|\boldsymbol{\tau}^{(0)} - \boldsymbol{\tau}^{(1)}\|_1.$$

Therefore, it suffices to construct $\mathbf{R}^{(2)}$ and $\mathbf{R}^{(3)}$. The construction involves two steps:

1. Ensure that the sum of the risk vector is correct within each stratum;

2. Ensure that the L_1 budget constraint is satisfied.

The L_1 distance needed to achieve each of these steps is bounded by $\|\boldsymbol{\tau}^{(0)} - \boldsymbol{\tau}^{(1)}\|_1$, and so $\mathbf{R}^{(2)}$ and $\mathbf{R}^{(3)}$ will have the required property.

Assume again that we are solving the minimization problem. Consider the j-th unobserved stratum (i.e., $i \in \mathcal{S}_{j,1}$) with associated upper and lower thresholds t^{lwr} and t^{upr} and suppose without loss of generality that $\tau_j^{(0)} \leq \tau_j^{(1)}$. Then, to achieve the first step, we simply raise t^{upr} until the sum of the risk vector is $\tau_j^{(1)}$ or $t^{\text{upr}} = 1$. If $t^{\text{upr}} = 1$, then we raise t^{lwr} until the sum of the risk vector is $\tau_j^{(1)}$. We note that since our risk vector has strictly increased, the L_1 distance between the original and new risk vectors is exactly the difference in their sums, i.e., $\tau_j^{(1)} - \tau_j^{(0)}$. Call the risk vector that results from performing this operation across all strata $\mathbf{R}'$. Then, it follows that

$$\|\mathbf{R}' - \hat{\mathbf{R}}\|_1 \leq \|\mathbf{R}^{(0)} - \hat{\mathbf{R}}\|_1 + \tau_j^{(1)} - \tau_j^{(0)} \leq \epsilon + \tau_j^{(1)} - \tau_j^{(0)}.$$

To achieve the second step, in strata $\mathcal{S}_{j,a}$ where $t^{\text{upr}} < 1$ and $t^{\text{lwr}} > 0$, we simply raise t^{upr} and lower t^{lwr} so as to ensure that the sum of the risk vector does not change. Doing so reduces the L_1 distance between the risk vector and $\hat{\mathbf{R}}$, so we do so until the L_1 budget constraint is satisfied. In particular, this process can be carried out until $t^{\text{upr}} = 1$ or $t^{\text{lwr}} = 0$ in every stratum. If the budget constraint is still not satisfied, then the simplified problem for $\boldsymbol{\tau}^{(1)}$ is not feasible, contrary to our assumption that there exists a solution $\mathbf{R}^{(1)}$. Therefore, the process halts, and, moreover, requires moving at most $\|\boldsymbol{\tau}^{(0)} - \boldsymbol{\tau}^{(1)}\|_1$ in L_1 distance. Call the resulting risk vector $\mathbf{R}^{(2)}$. Then, it follows that

$$\sum_{i \in \mathcal{S}_{j,0}} R_i^{(2)} = \tau_j^{(1)} \quad (j = 1, \dots, m), \qquad \|\mathbf{R}^{(2)} - \hat{\mathbf{R}}\|_1 \leq \epsilon.$$

The other constraints are satisfied trivially. We can construct $\mathbf{R}^{(3)}$ similarly, and the proof is complete for minimization. For maximization, the argument is exactly similar, except that we increase and decrease R_{i_0} and R_{i_1} instead of t^{lwr} and t^{upr}. □

We can extend the previous results to bound the error in the calculation of the coefficients themselves. (Recall Remark S3 on notation above.)

Lemma S22. *Let $V(\epsilon)$ denote the value of*

$$\begin{aligned} \underset{\mathbf{R}\in\mathbb{R}^n}{\text{Minimize}} \ & \frac{1}{n}\sum_{j=1}^{m} n_j \cdot \text{VAR}\left((R_i)_{i\in\mathcal{G}_j}\right) \\ \text{s.t.} \ & \tfrac{1}{n}\textstyle\sum_{i=1}^{n} |R_i - \hat{R}_i| \le \epsilon, \\ & \textstyle\sum_{i\in\mathcal{S}_{j,1}} R_i = \rho_j, \quad (j = 1,\ldots,m) \\ & R_i \le u_i, \quad (i = 1,\ldots,n) \\ & R_i \ge \ell_i. \quad (i = 1,\ldots,n) \end{aligned} \qquad [35]$$

To run Algorithm 1 with parameter ϵ, let

$$\boldsymbol{\eta} = (\eta_1, \ldots, \eta_m)$$

denote the step size of our grid search in each of the m strata on the $\boldsymbol{\tau} = (\tau_1, \ldots, \tau_m)$ scale. Let δ^* denote the true optimum of the base problem, and let $\delta^\dagger$ denote the value of the objective returned by applying Algorithm 1. Then

$$|\delta^* - \delta^\dagger| \le \frac{\frac{16}{n}\|\boldsymbol{\eta}\|_1 + \frac{8}{n}\|\boldsymbol{\eta}/\mathbf{n}\|_1 + 8m\gamma}{V(\epsilon)^2} + \frac{\frac{2}{n_1}\eta_1 + \frac{2}{n_j}\eta_j}{V(\epsilon)} + \frac{\frac{2}{n}\|\boldsymbol{\eta}\|_1}{V(\epsilon)},$$

where $\boldsymbol{\eta}/\mathbf{n} = (\eta_1/n_1, \ldots, \eta_m/n_m)$.

Proof. The proof is a straightforward exercise in bounding the various terms in Eq. (13) in Lemma S4. In particular, by Eq. (13), $|\delta^* - \delta^\dagger|$ is equivalent to

$$\left|\frac{a_0 \cdot b_0}{c_0 + d_0} - \frac{a_1 \cdot b_1}{c_1 + d_1}\right|, \qquad [36]$$

where

$$a_0 = \frac{1}{n}\sum_{j=1}^{m} \sigma_j \cdot \tau_j^* - (1-\sigma_j)\cdot\rho_j, \qquad a_1 = \frac{1}{n}\sum_{j=1}^{m} \sigma_j \cdot \tau_j^\dagger - (1-\sigma_j)\cdot\rho_j,$$

$$b_0 = \frac{\tau_j^* + \rho_j}{n_j} - \frac{\tau_1^* + \rho_1}{n_1}, \qquad b_1 = \frac{\tau_j^\dagger + \rho_j}{n_j} - \frac{\tau_1^\dagger + \rho_1}{n_1},$$

$$c_0 = \frac{1}{n}\sum_{i=1}^{n}(R_i^*)^2, \qquad c_1 = \frac{1}{n}\sum_{i=1}^{n}(R_i^\dagger)^2,$$

$$d_0 = \frac{1}{n}\sum_{j=1}^{m}\left(\frac{\rho_j + \tau_j^*}{n_j}\right)^2, \qquad d_1 = \frac{1}{n}\sum_{j=1}^{m}\left(\frac{\rho_j + \tau_j^\dagger}{n_j}\right)^2.$$

Here, $\mathbf{R}^*$ is the optimal risk vector—which, by Corollary S6, must correspond to an optimum of the simplified problem—and $\mathbf{R}^\dagger$ is the risk vector found by Algorithm 1.[15] Since the gap between the optimal and approximate solutions must be at least as large at any particular gridpoint as it is at the optimal gridpoint, we may assume without loss of generality that $\mathbf{R}^\dagger$ is the risk vector found by Algorithm 5 or Algorithm 8 at the gridpoint nearest $(\tau_1^*, \ldots, \tau_m^*)$. Finally, since only maximizing the objective involves approximation error, we may assume without loss of generality that $\mathbf{R}^\dagger$ is the risk vector found by Algorithm 8.

Now, it follows by a straightforward algebraic manipulation that

$$\delta^* - \delta^\dagger = a_0 \cdot b_0 \cdot \left(\frac{1}{c_0 + d_0} - \frac{1}{c_1 + d_1}\right) + a_0 \cdot (b_0 - b_1) \cdot \frac{1}{c_1 + d_1} + (a_0 - a_1) \cdot b_1 \cdot \frac{1}{c_1 + d_1}.$$

Therefore, we seek bounds A, B, and V such that

$$|a_0|, |a_1| \le A, \qquad |b_0|, |b_1| \le B, \qquad |c_0 + d_0|, |c_1 + d_1| \ge V$$

and Δ_a, Δ_b, and Δ_v such that

$$|a_0 - a_1| \le \Delta_a, \qquad |b_0 - b_1| \le \Delta_b, \qquad \left|\frac{1}{c_0 + d_0} - \frac{1}{c_1 + d_1}\right| \le \Delta_v.$$

Then, it will follow that

$$|\delta^* - \delta^\dagger| \le AB\Delta_v + \frac{A}{V}\Delta_b + \frac{B}{V}\Delta_a.$$

To find A, B, and V, we note that for any possible t_j and r_j, we have that

$$\sum_{j=1}^{m} |\sigma_j \cdot t_j| \le n, \qquad \sum_{j=1}^{m} |(1-\sigma_j)\cdot r_j| \le n,$$

[15] We note that Algorithms 5 and 8, for clarity, do not actually return the risk vector $\mathbf{R}$ itself, but could easily be modified to do so. This modification is likewise easily carried through to Algorithm 1.

whence $|a_i| \leq 2$ for $i = 0, 1$. Therefore we can take $A = 2$. In the same way, we have the bound

$$\left| \frac{\tau_j + \rho_j}{n_j} - \frac{\tau_1 + \rho_1}{n_1} \right| \leq 2,$$

so we take $B = 2$ as well. Finally, we have that $|c_0 + d_0| \geq V(\epsilon)$ and $|c_1 + d_1| \geq V(\epsilon)$ by definition, so we take $V = V(\epsilon)$.

Now, note that

$$|a_0 - a_1| = \left| \frac{1}{n} \sum_{j=1}^{m} \sigma_j \cdot (\tau_j^* - \tau_j^\dagger) \right| \leq \frac{1}{n} \sum_{j=1}^{m} \left| \tau_j^* - \tau_j^\dagger \right| \leq \frac{1}{n} \sum_{j=1}^{m} \eta_j = \frac{1}{n} \cdot \|\boldsymbol{\eta}\|_1,$$

so we take $\Delta_a = \frac{1}{n}\|\boldsymbol{\eta}\|_1$. Next, since $|\tau_j^* - \tau_j^\dagger| \leq \eta_j$ and similarly for τ_1^* and $\tau_1^\dagger$, we have that

$$|b_0 - b_1| \leq \left| \frac{\tau_j^* - \tau_j^\dagger}{n_j} \right| + \left| \frac{\tau_1^* - \tau_1^\dagger}{n_1} \right| \leq \frac{\eta_1}{n_1} + \frac{\eta_j}{n_j},$$

so we take $\Delta_b = \frac{\eta_1}{n_1} + \frac{\eta_j}{n_j}$. Finally, we have that

$$\left| \frac{1}{c_0 + d_0} - \frac{1}{c_1 + d_1} \right| = \left| \frac{c_1 - c_0 + d_1 - d_0}{(c_0 + d_0)(c_1 + d_1)} \right| \leq \frac{|c_0 - c_1| + |d_0 - d_1|}{V^2}.$$

Therefore, it only remains to bound $|c_0 - c_1|$ and $|d_0 - d_1|$. By Lemma S21, if $\mathbf{R}^\dagger$ were the *true* solution to the simplified problem, then $|c_0 - c_1|$ would be bounded by $\frac{4}{n}\|\boldsymbol{\eta}\|_1$, since the L_1 distance to the nearest gridpoint is at most $\|\boldsymbol{\eta}\|_1$. However, since $\mathbf{R}^\dagger$ is only an approximate solution, applying Lemma S20 gives that $|c_0 - c_1|$ is bounded by $2m\gamma + \frac{4}{n}\|\boldsymbol{\eta}\|_1$. Similarly, using the fact that

$$\left| \frac{\tau_j^* + \rho_j}{n_j} - \frac{\tau_j^\dagger + \rho_j}{n_j} \right| \leq \frac{\eta_j}{n_j},$$

we can apply Lemma S19 to obtain that $|d_0 - d_1|$ is less than or equal to $\frac{2}{n}\|\boldsymbol{\eta}/\mathbf{n}\|_1$. Thus, we can take $\Delta_v = (\frac{4}{n}\|\boldsymbol{\eta}\|_1 + \frac{2}{n}\|\boldsymbol{\eta}/\mathbf{n}\|_1 + 2m\gamma)/V^2$. Combining these bounds gives that

$$|\delta^* - \delta^\dagger| \leq \frac{\frac{16}{n}\|\boldsymbol{\eta}\|_1 + \frac{8}{n}\|\boldsymbol{\eta}/\mathbf{n}\|_1 + 8m\gamma}{V(\epsilon)^2} + \frac{\frac{2}{n_1}\eta_1 + \frac{2}{n_j}\eta_j}{V(\epsilon)} + \frac{\frac{2}{n}\|\boldsymbol{\eta}\|_1}{V(\epsilon)},$$

as desired. □

Lemma S22 gives intuition for how to choose the step sizes appropriately so as to minimize the error in the coefficients for a given amount of computation. In particular, since solving the parameterized problem requires roughly the same number of steps at each grid point, the computation scales like the number of gridpoints, i.e., like

$$\frac{1}{\prod_{j=1}^{m} \eta_j}.$$

The problem of choosing step sizes $\boldsymbol{\eta}$ so as to maximize accuracy for a given amount of computation is therefore essentially equivalent to the following optimization problem:

$$\begin{aligned} \underset{\boldsymbol{\eta} \in \mathbb{R}^m}{\text{Minimize}} \quad & \sum_{j=1} \lambda_j \eta_j \\ \text{s.t.} \quad & \eta_j > 0, \quad (j = 1, \dots, m) \\ & \prod_{j=1}^{m} \eta_j = M. \end{aligned}$$

Applying the first-order necessary KKT conditions yields that

$$\lambda_j = \nu \prod_{k \neq j} \eta_k, \qquad \text{i.e.,} \quad \lambda_j \eta_j = \nu M,$$

whence we have that

$$\eta_j = \frac{\sqrt[m]{M \prod_{j=1}^{m} \lambda_j}}{\lambda_j}.$$

In particular, the step sizes should be chosen so that they are inversely proportional to their weights in the error bound in Lemma S22.

Unfortunately, these weights vary with ϵ. For large ϵ, when $V(\epsilon)$ is close to zero, the weights are dominated by the first term, which is optimized when $\eta_1 = \cdots = \eta_m$. For small ϵ, when $V(\epsilon)$ is large, the weights are mixture of (somewhat larger) terms that would be optimized by $\eta_1 = \cdots = \eta_m$ and (somewhat smaller) terms that would be optimized by $\frac{\eta_1}{n_1} = \cdots = \frac{\eta_m}{n_m}$.

Thus, one reasonable heuristic that is likely to perform well across a range of ϵ is to choose η_j to be equal to some fixed η for all j. We use this heuristic in our experiments and software implementation.

Alternatively, to simplify the error bounds and eliminate the dependence on the data through the n_j, one could choose η_j to be proportional to n_j for all j. Making this choice gives the error bounds in Theorem S2, while the runtime bounds are given by Lemmata S10 and S18.

Lastly, since it is a convex problem, it is generally practical to compute $V(\epsilon)$. However, to obtain a bound on the error entirely in terms of the input parameters, we can use the following lemma.

Lemma S23. *Let $V(\epsilon)$ be defined as in Lemma S22. Then*

$$V(\epsilon) \geq \left[\frac{1}{n}\sum_{j=1}^{m} \text{Var}\left((\hat{R}_i)_{i\in\mathcal{S}_{j,1}}\right)\right] - 4\epsilon. \qquad [37]$$

Proof. Let $\mathbf{R}^*$ be the solution to the optimization problem defining $V(\epsilon)$. Then, we note that $\|\mathbf{R}^* - \hat{\mathbf{R}}\|_1 \leq \epsilon$, and, moreover, that

$$V(\epsilon) - \frac{1}{n}\sum_{j=1}^{m} \text{Var}\left((\hat{R}_i)_{i\in\mathcal{S}_{j,1}}\right) = \frac{1}{n}\left[\left(\sum_{i=1}^{n}(R_i^*)^2 - \hat{R}_i^2\right) + \left(\sum_{j=1}^{m}\left[\frac{\sum_{i\in\mathcal{G}_j} R_i^*}{n_j}\right]^2 - \left[\frac{\sum_{i\in\mathcal{G}_j} \hat{R}_i}{n_j}\right]^2\right)\right].$$

Applying Lemma S19 to the first term yields a bound of 2ϵ. For the second term, we note that

$$\sum_{j=1}^{m}\left|\frac{\sum_{i\in\mathcal{G}_j} R_i^*}{n_j} - \frac{\sum_{i\in\mathcal{G}_j} \hat{R}_i}{n_j}\right| \leq \sum_{i=1}^{n}|R_i^* - \hat{R}_i| \leq \epsilon,$$

and so we can apply Lemma S19 to obtain a bound of 2ϵ on the second term as well. Combining these bounds gives the desired result. □

Algorithm 2 Piecewise quadratic function evaluation

Input: The collections $\mathbf{\Delta}$ and $\mathbf{K}$, as well as the L_1 "budget" ϵ.
Output: The value of $\Sigma(\Delta^*)$, where $\epsilon(\Delta^*) = \epsilon$.

Set $N \leftarrow$ `length`$(\mathbf{\Delta})$
Initialize $i \leftarrow 1$ {*Pointer to current position in* $\mathbf{\Delta}$}
Initialize $\varepsilon \leftarrow 0$ {*Budget used so far*}
Initialize $t \leftarrow 0$ {*Gap between current and next value of* $\mathbf{\Delta}$}
Initialize $\Sigma \leftarrow 0$ {*Cumulative sum of squares*}
while $\varepsilon + K_i(\Delta_{i+1} - \Delta_i) < \epsilon$ **and** $i < N$ **do**
  Set $\varepsilon \leftarrow \varepsilon + K_i(\Delta_{i+1} - \Delta_i)$
  Set $\Sigma \leftarrow \Sigma - K_i \cdot \Delta_i \cdot (\Delta_{i+1} - \Delta_i) + \frac{K_i}{2} \cdot (\Delta_{i+1} - \Delta_i)^2$
  Set $i \leftarrow i + 1$
end while
if $K_i = 0$ **then**
  return Σ {$K_i = 0$ *if and only if the budget was exactly exhausted on the last iteration of the loop*}
else if $i = N$ **then**
  return Σ {$i = N$ *if and only if we have made it to the end of* $\mathbf{\Delta}$*, i.e., to* $\Delta = 0$}
else
  Set $t \leftarrow (\epsilon - \varepsilon)/K_i$
  Set $\Sigma \leftarrow \Sigma - K_i \cdot \Delta_i \cdot t + \frac{K_i}{2} \cdot t^2$
  return Σ
end if

Algorithm 3 Minimization algorithm (sum adjustment)

Input: The bounds $\boldsymbol{\ell}$ and $\mathbf{u}$, the estimates $\hat{\mathbf{R}}$, and the sum μ.
Output: A risk vector $\mathbf{R}$ of the form in Lemma S8 minimizing $\|\mathbf{R} - \hat{\mathbf{R}}\|_1$ and satisfying $\sum_{i=1}^{n} R_i = \mu$, along with the ending k^{lwr}, k^{upr}, i^{lwr}, i^{upr}, t^{lwr}, and t^{upr}.

Set `pts` to be the concatenation of $\boldsymbol{\ell}$, $\hat{\mathbf{R}}$, and $\mathbf{u}$ in ascending order {*Points at which rates can change*}
Set $\mathbf{R} \leftarrow \hat{\mathbf{R}}$ {*Risk vector*}
Set $n \leftarrow$ `length`(`pts`)
Set $i^{\text{lwr}} = 1$, $i^{\text{upr}} = n$ {*Indices of next values at which rates might change*}
Set $t^{\text{lwr}} \leftarrow$ `pts`$[i^{\text{lwr}}]$, $t^{\text{upr}} \leftarrow$ `pts`$[i^{\text{upr}}]$ {*Thresholds*}
Set $k^{\text{lwr}} \leftarrow 0$, $k^{\text{upr}} \leftarrow 0$ {*Number of active indices*}
$D \leftarrow \mu - \sum_{i=1}^{n} R_i$ {*Difference between required sum and actual sum*}
if $D > 0$ **then**
    $t^{\text{nxt}} \leftarrow$ `pts`$[i^{\text{lwr}} + 1]$
    $d \leftarrow 0$ {*Change in sum from moving from t^{lwr} to t^{nxt}*}
    while $D > d$ **and** $i^{\text{lwr}} < n$ **do**
        $D \leftarrow D - d$
        $i^{\text{lwr}} \leftarrow i^{\text{lwr}} + 1$
        $t^{\text{lwr}} \leftarrow t^{\text{nxt}}$
        $t^{\text{nxt}} \leftarrow$ `pts`$[i^{\text{lwr}} + 1]$
        if t^{nxt} corresponds to an element of $\hat{\mathbf{R}}$ **then**
            $k^{\text{lwr}} \leftarrow k^{\text{lwr}} + 1$
        else if t^{nxt} corresponds to an element of $\mathbf{u}$ **then**
            $k^{\text{lwr}} \leftarrow k^{\text{lwr}} - 1$
        end if
        $d \leftarrow k^{\text{lwr}}(t^{\text{nxt}} - t^{\text{lwr}})$
    end while
    if $i^{\text{lwr}} < n$ **then**
        $t^{\text{lwr}} \leftarrow t^{\text{lwr}} + \frac{D}{k^{\text{lwr}}}$
    end if
else if $D < 0$ **then**
    {*Similar steps to the $D > 0$ case but adapted for the upper threshold*}
end if

return $\mathbf{R}$, k^{lwr}, k^{upr}, i^{lwr}, i^{upr}, t^{lwr}, and t^{upr}

Algorithm 4 Minimization algorithm (single stratum)

Input: The bounds $\boldsymbol{\ell}$ and $\mathbf{u}$, the estimates $\hat{\mathbf{R}}$, and the sum μ.
Output: The collections $\boldsymbol{\Delta}$ and $\mathbf{K}$.

Initialize `pts`, $\mathbf{R}$, t^{lwr}, t^{upr}, k^{lwr}, k^{upr}, i^{lwr}, and i^{upr} as in Algorithm 3
Set $n \leftarrow$ `length(pts)`
Set $\varepsilon \leftarrow 0$ {*Amount of budget expended so far*}
Set $\Delta \leftarrow t^{\text{upr}} - t^{\text{lwr}}$ {*Gap between thresholds*}
Using Algorithm 3, update $\mathbf{R}$, k^{lwr}, k^{upr}, i^{lwr}, i^{upr}, t^{lwr}, and t^{upr} {*Ensure that sum is correct*}
Set $\epsilon \leftarrow \epsilon - \|\mathbf{R} - \hat{\mathbf{R}}\|_1$, returning that the problem is infeasible if the result is negative
if $n = 1$ **then**
  return $\boldsymbol{\Delta} = (\Delta_0)$ and $\mathbf{K} = 0$
end if
$D^{\text{lwr}} \leftarrow k^{\text{lwr}}(\texttt{pts}[i^{\text{lwr}} + 1] - t^{\text{lwr}})$ {*Cost of moving from lower threshold to next change point*}
$D^{\text{upr}} \leftarrow k^{\text{upr}}(t^{\text{upr}} - \texttt{pts}[i^{\text{upr}} - 1])$ {*Cost of moving from upper threshold and next change point*}
$D \leftarrow \min(D^{\text{lwr}}, D^{\text{upr}})$ {*Smaller of the costs*}
$K \leftarrow 0$
while $i^{\text{upr}} - i^{\text{lwr}} > 1$ **do**
  Append Δ to $\boldsymbol{\Delta}$ and K to $\mathbf{K}$
  if $D^{\text{lwr}} = D$ **then**
    $i^{\text{lwr}} \leftarrow i^{\text{lwr}} + 1$ {*Increment lower active index*}
    $t^{\text{lwr}} \leftarrow \texttt{pts}[i^{\text{lwr}}]$ {*Update the lower threshold*}
    if t^{lwr} came from $\hat{\mathbf{R}}$ **then**
      $k^{\text{lwr}} \leftarrow k^{\text{lwr}} + 1$ {*Increment k^{lwr} if an index is activated*}
    else if t^{lwr} came from $\mathbf{u}$ **then**
      $k^{\text{lwr}} \leftarrow k^{\text{lwr}} - 1$ {*Decrement k^{lwr} if an index is deactivated*}
    end if
    $D^{\text{upr}} \leftarrow D^{\text{upr}} - D^{\text{lwr}}$ {*Calculate new upper active gap*}
    $t^{\text{upr}} \leftarrow t^{\text{upr}} - \frac{D^{\text{lwr}}}{k^{\text{upr}}}$ {*Calculate new upper threshold*}
    $D^{\text{lwr}} \leftarrow k^{\text{lwr}}(\texttt{pts}[i^{\text{lwr}} + 1] - t^{\text{lwr}})$ {*Calculate new lower active gap*}
  else
    {*Similar steps as in previous branch, adapted to the upper threshold*}
  end if
  $D \leftarrow \min(D^{\text{lwr}}, D^{\text{upr}})$
  $\Delta \leftarrow t^{\text{upr}} - t^{\text{lwr}}$
  $K \leftarrow 2 \cdot \frac{k^{\text{lwr}} \cdot k^{\text{upr}}}{k^{\text{lwr}} + k^{\text{upr}}}$
end while
Append 0 to $\boldsymbol{\Delta}$ and $\mathbf{K}$
return $\boldsymbol{\Delta}, \mathbf{K}$

Algorithm 5 Minimization algorithm (combining strata)

Input: The collections $\boldsymbol{\Delta}^{(0)}$, $\mathbf{K}^{(0)}$, $\boldsymbol{\Delta}^{(1)}$, and $\mathbf{K}^{(1)}$.
Output: A pair $(\boldsymbol{\Delta}^{(2)}, \mathbf{K}^{(2)})$ representing the sum of functions $\epsilon^{(0)}(\Delta) + \epsilon^{(1)}(\Delta)$ and $\Sigma^{(0)}(\Delta) + \Sigma^{(1)}(\Delta)$.

Initialize $i_0 \leftarrow 0$, $i_1 \leftarrow 0$ {*Pointers to smallest indices not yet combined*}
Initialize $K_0 \leftarrow 0$, $K_1 \leftarrow 0$, $\Delta_0 \leftarrow 1$, $\Delta_1 \leftarrow 1$ {*Current values of the parameters*}
while $i_0 \leq \texttt{length}(\boldsymbol{\Delta}_0)$ **and** $i_1 \leq \texttt{length}(\boldsymbol{\Delta}_1)$ **do**
  if $\Delta_0 > \Delta_1$ **then**
    Set $i_0 \leftarrow i_0 + 1$, $K_0 \leftarrow K^{(0)}_{i_0}$, and $\Delta_0 \leftarrow \Delta^{(0)}_{i_0}$
    Append Δ_0 to $\boldsymbol{\Delta}^{(2)}$ and $K_0 + K_1$ to $\mathbf{K}^{(2)}$
  else
    Set $i_1 \leftarrow i_1 + 1$, $K_1 \leftarrow K^{(1)}_{i_1}$, and $\Delta_1 \leftarrow \Delta^{(1)}_{i_1}$
    Append Δ_1 to $\boldsymbol{\Delta}^{(2)}$ and $K_0 + K_1$ to $\mathbf{K}^{(2)}$
  end if
end while
return $\boldsymbol{\Delta}^{(2)}$ and $\mathbf{K}^{(2)}$

Algorithm 6 Maximization algorithm (sum adjustment)

Input: The bounds $\boldsymbol{\ell}$ and $\mathbf{u}$, the estimates $\hat{\mathbf{R}}$, and the sum μ.
Output: A risk vector $\mathbf{R}$ of the form in Lemma S13 minimizing $\|\mathbf{R} - \hat{\mathbf{R}}\|_1$ and satisfying $\sum_{i=1}^{n} R_i = \mu$, along with i^{lwr}, i^{upr}.

Set $\mathbf{R} \leftarrow \hat{\mathbf{R}}$ {*Risk vector*}
Set $n \leftarrow \texttt{length}(\mathbf{R})$
Set $i^{\text{lwr}} = 1$, $i^{\text{upr}} = n$ {*Pivots*}
$D \leftarrow \mu - \sum_{i=1}^{n} R_i$ {*Difference between required sum and actual sum*}
if $D > 0$ **then**
    $d \leftarrow u_{i^{\text{upr}}} - R_{i^{\text{upr}}}$
    while $D > d$ **and** $i^{\text{upr}} > 0$ **do**
        $R_{i^{\text{upr}}} \leftarrow u_{i^{\text{upr}}}$
        $D \leftarrow D - d$
        $i^{\text{upr}} \leftarrow i^{\text{upr}} - 1$
        $d \leftarrow u_{i^{\text{upr}}} - R_{i^{\text{upr}}}$
    end while
    $R_{i^{\text{upr}}} \leftarrow R_{i^{\text{upr}}} + D$
else if $D < 0$ **then**
    {*Similar steps to the* $D > 0$ *case but adapted for the lower pivot*}
end if
return $\mathbf{R}$, i^{lwr}, and i^{upr}.

Algorithm 7 Maximization algorithm (single stratum)

Input: The bounds $\boldsymbol{\ell}$ and $\mathbf{u}$, the estimates $\hat{\mathbf{R}}$, the sum μ, and the step size γ.
Output: The collection $\boldsymbol{\Sigma}$.

Initialize $\mathbf{R}$, i^{lwr}, and i^{upr} as in Algorithm 6
Set $n \leftarrow \texttt{length}(R)$
Set $\varepsilon \leftarrow 0$ {*Amount of budget expended so far*}
Using Algorithm 6, update $\mathbf{R}$, i^{lwr}, and i^{upr} {*Ensure that sum is correct*}
Set $\varepsilon \leftarrow \|\mathbf{R} - \hat{\mathbf{R}}\|_1$ and $\epsilon \leftarrow \epsilon - \varepsilon$, returning that the problem is infeasible if $\varepsilon > \epsilon$
while $\varepsilon \geq \gamma$ **do**
  Append ∞ to $\boldsymbol{\Sigma}$ {*Sentinel value to indicate infeasibility*}
  $\varepsilon \leftarrow \varepsilon - \gamma$
end while
$\Delta^{\text{lwr}} \leftarrow R_{i^{\text{lwr}}} - \ell_{i^{\text{lwr}}}$
$\Delta^{\text{upr}} \leftarrow u_{i^{\text{upr}}} - R_{i^{\text{upr}}}$
$\Delta \leftarrow \min(\Delta^{\text{lwr}}, \Delta^{\text{upr}})$
$\Sigma \leftarrow \sum_{i=1}^{n} R_i^2$ {*Sum of squares*}
while $i^{\text{upr}} > i^{\text{lwr}}$ **do**
  if $\Delta \geq \gamma - \varepsilon$ **then**
    $g \leftarrow \gamma - \varepsilon$
    $\Sigma \leftarrow \Sigma + 2g \cdot (g + [R_{i^{\text{upr}}} - R_{i^{\text{lwr}}}])$ {*If next step would exceed step size, record change*}
    Append Σ to $\boldsymbol{\Sigma}$
    $R_{i^{\text{lwr}}} \leftarrow R_{i^{\text{lwr}}} - g$, $R_{i^{\text{upr}}} \leftarrow R_{i^{\text{upr}}} + g$ {*Update risk vector at pivots*}
    $\Delta \leftarrow \Delta - g$, $\Delta^{\text{lwr}} \leftarrow \Delta^{\text{lwr}} - g$, $\Delta^{\text{upr}} \leftarrow \Delta^{\text{upr}} - g$ {*Update gaps*}
    $\varepsilon \leftarrow 0$ {*Only adjust step size on first iteration*}
  else
    if $\Delta^{\text{lwr}} = \Delta$ **then**
      $\Sigma \leftarrow \Sigma + 2\Delta \cdot (\Delta + [R_{i^{\text{upr}}} - R_{i^{\text{lwr}}}])$
      $R_{i^{\text{lwr}}} \leftarrow \ell_{i^{\text{lwr}}}$, $R_{i^{\text{upr}}} \leftarrow R_{i^{\text{upr}}} + \Delta$
      $i^{\text{lwr}} \leftarrow i^{\text{lwr}} + 1$ {*Increment lower active index*}
      $\Delta^{\text{lwr}} \leftarrow R_{i^{\text{lwr}}} - \ell_{i^{\text{lwr}}}$ {*Update the lower gap*}
      $\Delta \leftarrow \min(\Delta^{\text{lwr}}, \Delta^{\text{upr}})$ {*Update the gap*}
    else
      {*Similar steps as in previous branch, adapted to the upper pivot*}
    end if
  end if
end while
Repeatedly append Σ to $\boldsymbol{\Sigma}$ until it has the appropriate length
return $\boldsymbol{\Sigma}$

Algorithm 8 Maximization algorithm (combining strata)

Input: The collections $\mathbf{\Sigma}^{(0)}$ and $\mathbf{\Sigma}^{(1)}$.
Output: A collection $\mathbf{\Sigma}^{(2)}$ representing the (approximate) maximum objective across both collections.

Initialize $i_0 \leftarrow 1$, $i_1 \leftarrow 1$ {*Pointers to indices currently being combined*}
Initialize $M \leftarrow -\infty$ {*Current maximum*}
for $i = 1, \ldots, \epsilon/\gamma + 1$ **do**
  $i_0 \leftarrow 1$, $i_1 \leftarrow i$, $M \leftarrow -\infty$ {*Reset pointers and maximum for each budget*}
  while $i_0 \leq i + 1$ **do**
    $M \leftarrow \max(M, \Sigma^{(0)}_{i_0} + \Sigma^{(1)}_{i_1})$ {*Find the maximum across gridpoints whose total budget is* $(i-1) \cdot \gamma$}
    $i_0 \leftarrow i_0 + 1$, $i_1 \leftarrow i_1 - 1$
  end while
  Append M to $\mathbf{\Sigma}^{(2)}$
end for
return $\mathbf{\Sigma}^{(2)}$

3. Estimation of the Non-Parametric Estimand by Linear Regression

To assess the quality of risk-adjusted regression as an estimator of the non-parametric estimand in Eq. (2), we construct synthetic datasets based on the NYPD data. On this synthetic dataset, we compare the true value of the risk adjusted disparities—as defined in Eq. (2)—to estimates from a risk-adjusted regression.

To construct the synthetic datasets, we first use the real NYPD data to estimate the probability of frisk, $\Pr(A = 1 \mid \tilde{X})$, conditional on the same observed pre-frisk covariates $\tilde{X}$ used to estimate risk in the main analysis, excluding race, suspected crime, and precinct.[16] We similarly estimate the probability a weapon is recovered on a frisked individual, $\Pr(W = 1 \mid \tilde{X}, A = 1)$, again conditional on the same pre-frisk covariates. In both cases, we use logistic regression to estimate the probabilities. The frisk model is trained on all data from 2008 and 2009, and the weapon-recovery model is trained on the subset of stops of the same in which an individual was frisked.

With the resulting covariate estimates $\hat{\boldsymbol{\beta}}_{\text{weapon}}$ and $\hat{\boldsymbol{\beta}}_{\text{frisk}}$, for the k-th iteration of the $m = 100$ simulation instances, we generate a population of $n = 100{,}000$ observations as follows:

1. Sample noise terms $\boldsymbol{\epsilon}_{\text{weapon}} \sim \mathcal{N}(0, \frac{1}{50} \cdot I)$ and $\boldsymbol{\epsilon}_{\text{frisk}} \sim \mathcal{N}(0, \frac{1}{50} \cdot I)$, resulting in new weapon possession and frisk model coefficients $\boldsymbol{\beta}_{k,\text{weapon}} = \hat{\boldsymbol{\beta}}_{\text{weapon}} + \boldsymbol{\epsilon}_{\text{weapon}}$ and $\boldsymbol{\beta}_{k,\text{frisk}} = \hat{\boldsymbol{\beta}}_{\text{frisk}} + \boldsymbol{\epsilon}_{\text{frisk}}$.

2. Sample, with replacement, n covariate vector and race pairs $(\tilde{X}_i, C_i)_{i=1,\dots,n}$ from the original NYPD dataset covering the years 2010 and 2011.

3. For all $i = 1, \dots, n$:

   (a) Define the probability the i-th individual is frisked: $p_i = \text{logit}^{-1}(\boldsymbol{\beta}_{k,\text{frisk}}^\top \tilde{X}_i)$. Then sample $A_i \sim \text{Bernoulli}(p_i)$.

   (b) Define the probability the i-th individual has a weapon: $R_i = \text{logit}^{-1}(\boldsymbol{\beta}_{k,\text{weapon}}^\top \tilde{X}_i)$. Then, sample $W_i \sim \text{Bernoulli}(R_i)$.

The above procedure produces a set of tuples, $\Omega^j = \{(\tilde{X}_i, C_i, A_i, R_i, W_i)\}_{i=1,\dots,n}$, each of which is a synthetic population. On this population, we compute the ground-truth risk-adjusted disparities via Eq. (2).[17] These disparities are typically non-zero; note, though, that there is no disparate *treatment* in this example since, by construction, the probability p_i of frisking an individual does not explicitly depend on race. The disparate impact we measure in this scenario thus arises from decisions that are simply not appropriately tailored to risk, as in *Griggs*.

We next evaluate the ability of our own risk-adjusted regression to recover the ground-truth disparate impact. To do so, we first divide Ω^j into two random sets of equal size, Ω_1^j and Ω_2^j. On Ω_1^j, we use logistic regression to estimate the probability that frisked individuals are found to have a weapon,[18] based on the pre-frisk covariates $\tilde{X}$. This model is trained on the subset of stops in Ω_1^j in which a frisk was carried out. We then use this fitted model to predict *ex ante* risk $\hat{R}_i$ for every stop in Ω_2^j. Lastly, we fit a linear risk-adjusted regression on Ω_2^j to estimate the Black-White and Hispanic-White disparities.

We note that to simulate real-world settings, we have ensured that, by construction, the linear probability model used to estimate disparities is misspecified. Nevertheless, it is a reasonably robust estimator of the true disparity, as can be seen in Figure S6. For both Black and Hispanic pedestrians in the synthetic data, our risk-adjusted estimates of disparate impact are very close to the ground-truth estimands, with model misspecification leading to slight overestimates in some instances, and slight underestimates in others.

[16] We exclude race to demonstrate that disparate impact can occur in the absence of disparate treatment. We exclude suspected crime and precinct because the large number of strata for these covariates leads to sampling difficulties.

[17] The definition in Eq. (2) involves conditioning on risk, which is continuous. This is problematic on a finite population, as there may be only one individual with any given risk. Binning risks avoids this issue, and, in this case, we get nearly identical values for any appropriately small bin size.

[18] To avoid risk estimates not being well defined when certain feature levels appear in Ω_2^j but not Ω_1^j, the models are fit using penalized maximum likelihood with the `glmnet` package and an elastic net penalty of $\alpha = \frac{1}{10}$.

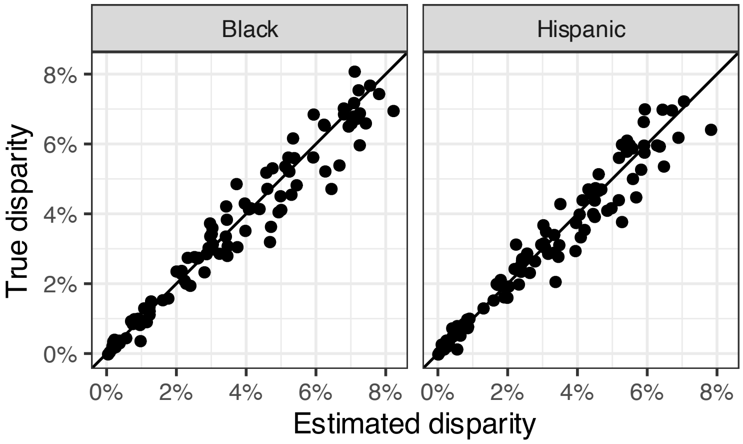

Fig. S6. *Estimates of disparate impact using risk-adjusted regressions on* $m = 100$ *synthetic datasets, each consisting of* $n = 1{,}000{,}000$ *simulated stops. Despite model misspecification, the risk-adjusted regressions coefficient is a reasonably robust estimator of the true disparity.*

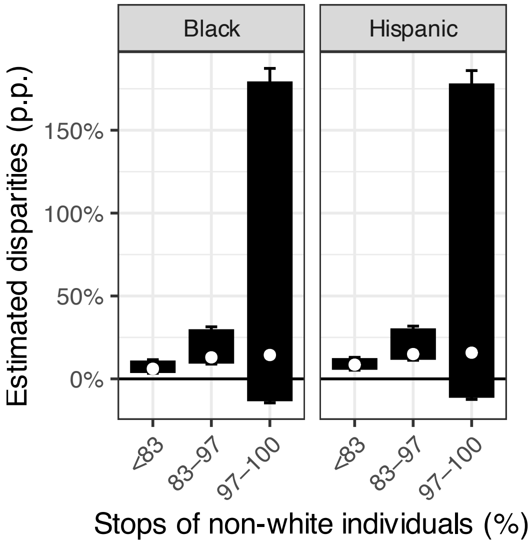

Fig. S7. *Estimates of disparate impact across precincts stratified by racial composition of stops, with the black bands showing the range of possible estimates if the odds ratio of estimated risk and true risk differ by at most* $\epsilon = 0.7$ p.p.

4. Accounting for factors beyond risk

Our analysis in the main text implicitly assumed that risk of weapon possession is the only legitimate consideration for carrying out a frisk—an assumption motivated by the fact that a frisk is legally allowed only to ensure officer safety. Officers, however, are not required to frisk every individual whom they are legally allowed to frisk (i.e., the law only sets a lower bar), leaving open the possibility that risk is not the only factor officers consider when making frisk decisions.[19] For example, in theory, resource-constrained officers might choose to frisk only the riskiest individuals they stop, effectively setting the bar to frisk individuals higher than the law demands. If resources differ across neighborhoods, which in turn correlate with racial composition, then the risk-adjusted disparities we see may accordingly have a policy-relevant justification.

In Figure S7, we aim to account for this possibility by repeating our analysis on three subsets of stops stratified by geography. Specifically, we split the 76 police precincts in our data into three bins based on the racial composition of stopped individuals. Across strata, we find qualitatively similar estimates of disparate impact, corroborating our main results. These within-strata estimates are also reasonably robust to mismeasurement of risk, as indicated by the black bands, although less so than our main result—a 0.7 p.p. average absolute difference in risk would potentially be sufficient to change the sign of the disparity in the most non-White neighborhoods, or to mask a disparity roughly ten times larger than our estimate.

That factors beyond risk may justifiably inform frisk decisions can be viewed as a form of omitted-variable bias, but one distinct in kind from that typically considered in studies of discrimination. Our disparate impact analysis is predicated on the understanding that risk, appropriately defined and estimated, captures nearly all policy-relevant considerations. Although it can be important in practice to accommodate exceptions when there is a clearly articulated rationale—as we have done above—care must be taken not to blindly adjust for every available factor, lest one re-introduce included-variable bias.

[19] In *Floyd*, the court found that officers at times frisked individuals even in the absence of safety concerns, in violation of the Fourth Amendment of the U.S. Constitution (29).

5. Additional figures

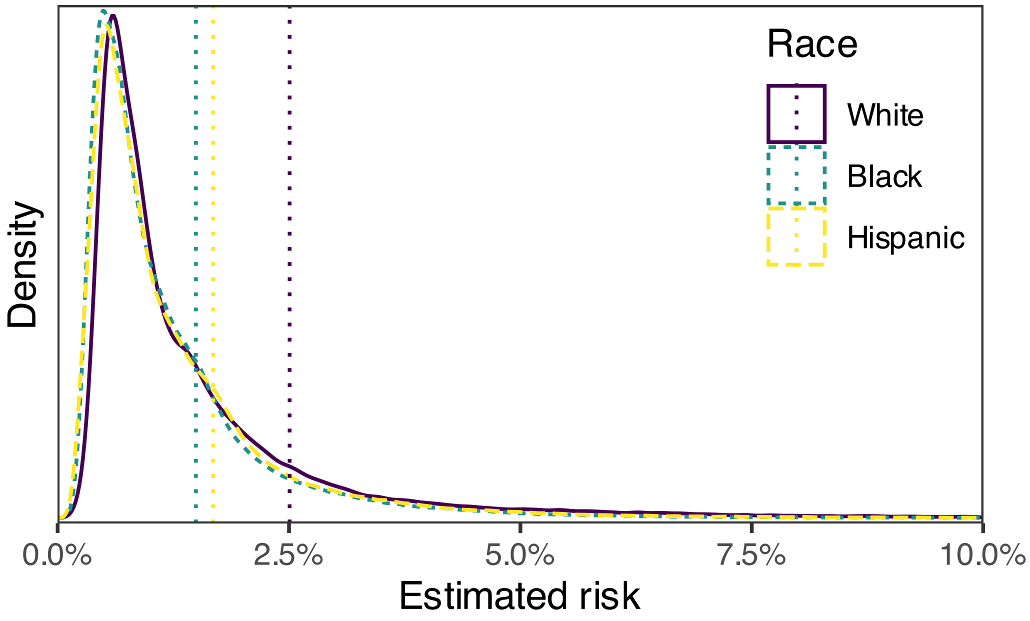

Fig. S8. *Distributions of the estimated* ex ante *probability of carrying a weapon for stopped pedestrians, on a log scale. The vertical lines indicate each group's average risk (i.e., the estimated rate at which stopped members of the group carry weapons): 2.7% for White pedestrians, 1.5% for Black pedestrians, and 1.7% for Hispanic pedestrians.*

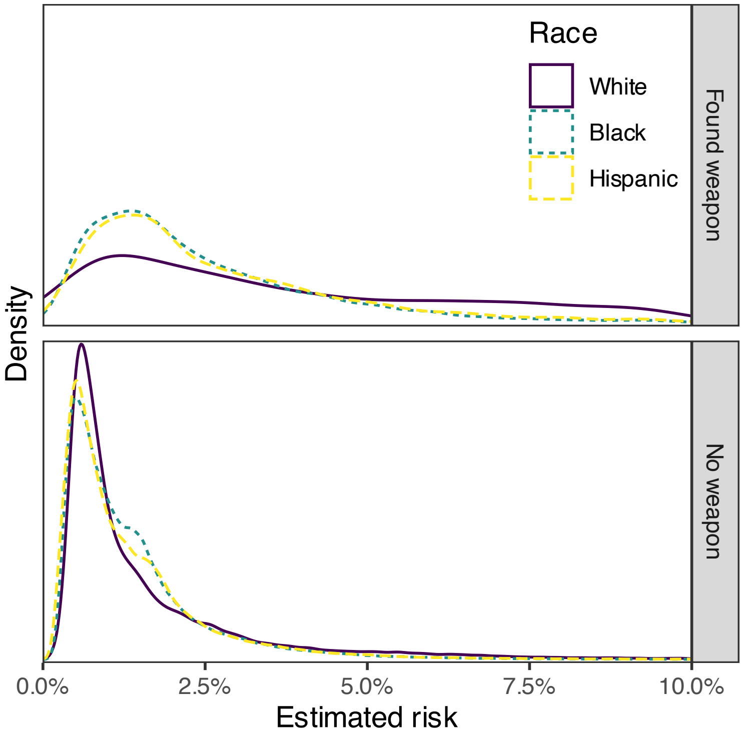

Fig. S9. *Distributions of the estimated* ex ante *probability of carrying a weapon for stopped pedestrians, on a log scale among frisked individuals, faceted by whether or not a weapon was found.*

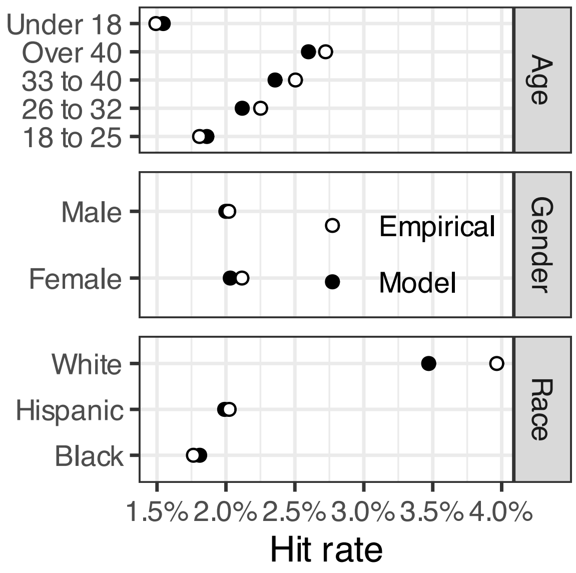

(a) Demographic groups

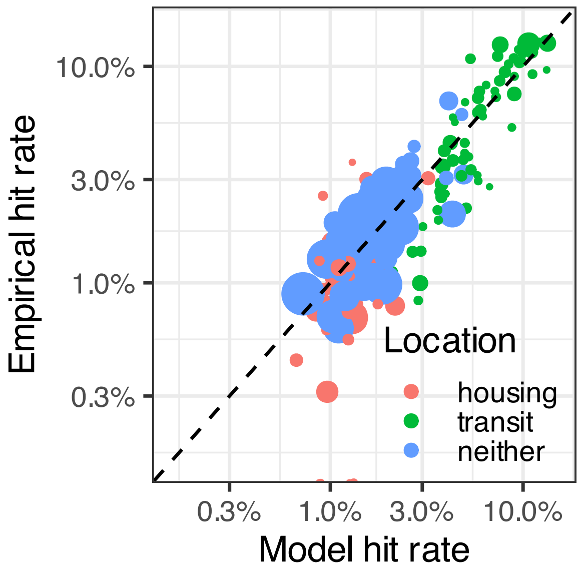

(b) Locations

Fig. S10. *Comparison of model-predicted versus empirical weapon recovery rate ("hit rate"). Figure S10a shows that the model-predicted hit rates are close to their empirical counterparts, conditional on values of age, race, and gender. In Figure S10b, stops are binned by precinct and stop location. Points are plotted for each bin with more than 100 stops, sized by the number of stops, with colors representing the stop location type: transit, housing, or other. The plotted points are near the diagonal, suggesting that the outcome model is well-calibrated and predicts well over the full range of hit rates and frisk rates, respectively. The model itself achieves an AUC of 81% on the second half of the data.*

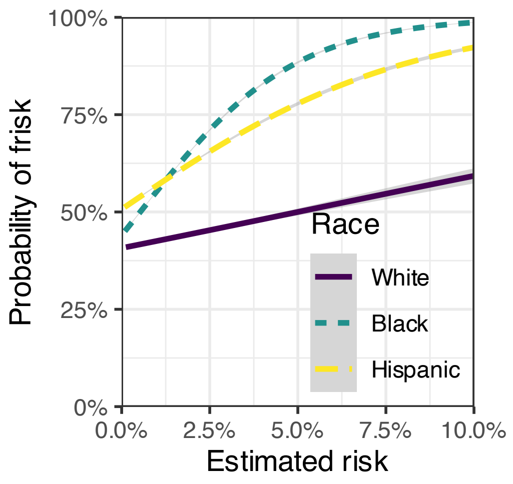

Fig. S11. *Frisk rates vs. risk (where risk has been estimated without race as a covariate), as estimated via logistic regression curves fit separately for each race group. Across risk levels, stopped Black and Hispanic pedestrians are frisked substantially more frequently than comparably risky White individuals, indicative of disparate impact.*

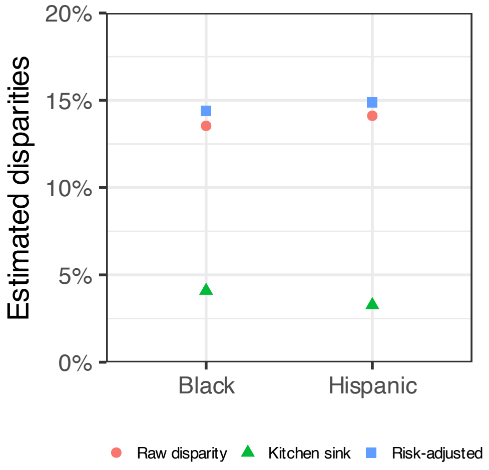

Fig. S12. *Racial gaps in frisk rates adjusting for different sets of covariates, where the y-axis shows the percentage point difference relative to stopped White individuals. The red dots show the raw disparities in frisk rates. As a measure of discrimination, raw disparities suffer from omitted-variable bias: there may, in theory, be legitimate reasons why Black and Hispanic pedestrians are more likely to be frisked. The green triangles show the estimated race effects in a kitchen-sink regression, adjusting for all pre-frisk covariates. These estimates suffer from included-variable bias because they adjust for features that are correlated with race but unrelated to risk. The blue squares show the results of our risk-adjusted regression, adjusting exclusively for estimated risk of weapon possession, where risk has been estimated without race as a covariate. In all cases, estimated standard errors are less than 0.2 percentage points, and so are not visible in the plot.*

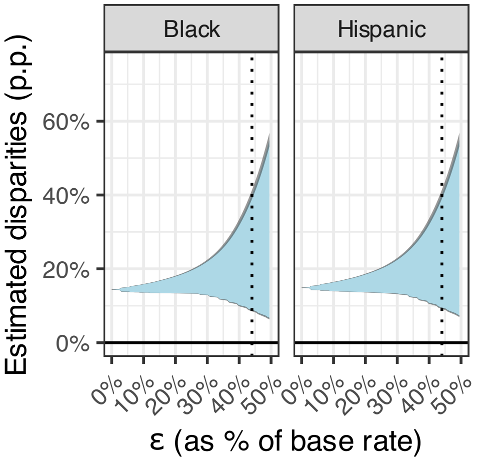

Fig. S13. *Sensitivity of the risk-adjusted disparities in frisk decisions to mismeasurement of risk, where risk has been estimated without race as a covariate. The blue bands bound our estimates of disparate impact as a function of the average absolute difference between the true and estimated risks ϵ, relative to the base rate (1.7%). The dotted line at 44% ($\epsilon = 0.7$ p.p.) corresponds to a simulated situation with severe confounding. The grey bands represent 95% percentile bootstrapped confidence intervals ($N = 1000$; 19). The step size for the grid search over group-level total risks was 0.05 p.p. for all groups, and $\gamma = 0.01$ p.p. was the approximation parameter for the maximization routine.*

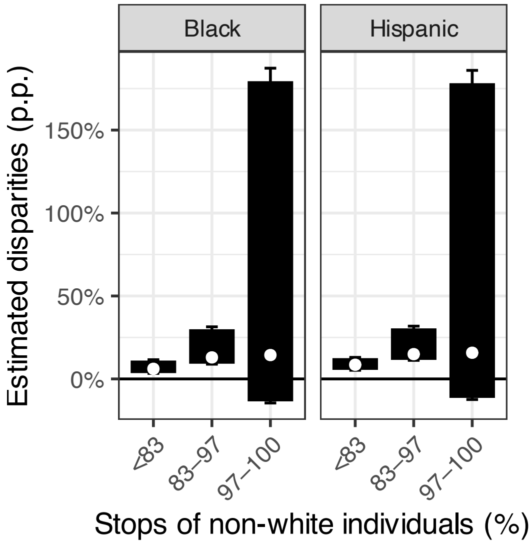

Fig. S14. *Estimates of disparate impact across precincts stratified by racial composition of stops, with the black bands showing the range of possible estimates if the odds ratio of estimated risk and true risk differ by at most* $\epsilon = 0.7\ \mathrm{p.p.}$ *Here, risk is estimated without race as a covariate.*

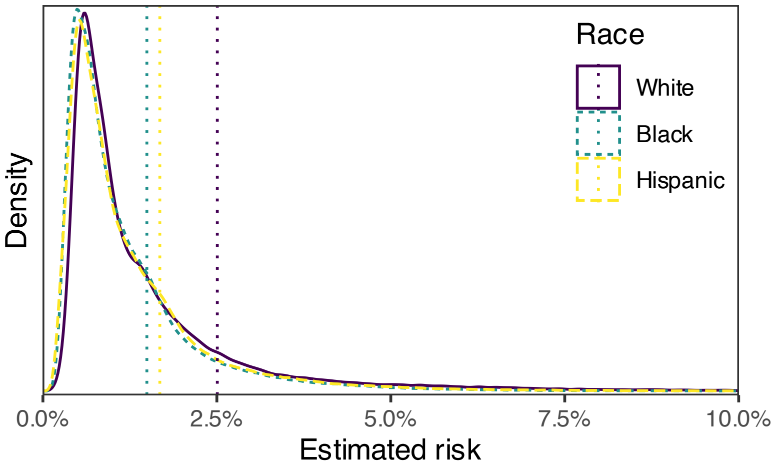

Fig. S15. *Distributions of the estimated* ex ante *probability of carrying a weapon for stopped pedestrians, on a log scale and estimated without race as a covariate. The vertical lines indicate each group's average risk (i.e., the estimated rate at which stopped members of the group carry weapons): 2.7% for White pedestrians, 1.5% for Black pedestrians, and 1.7% for Hispanic pedestrians.*

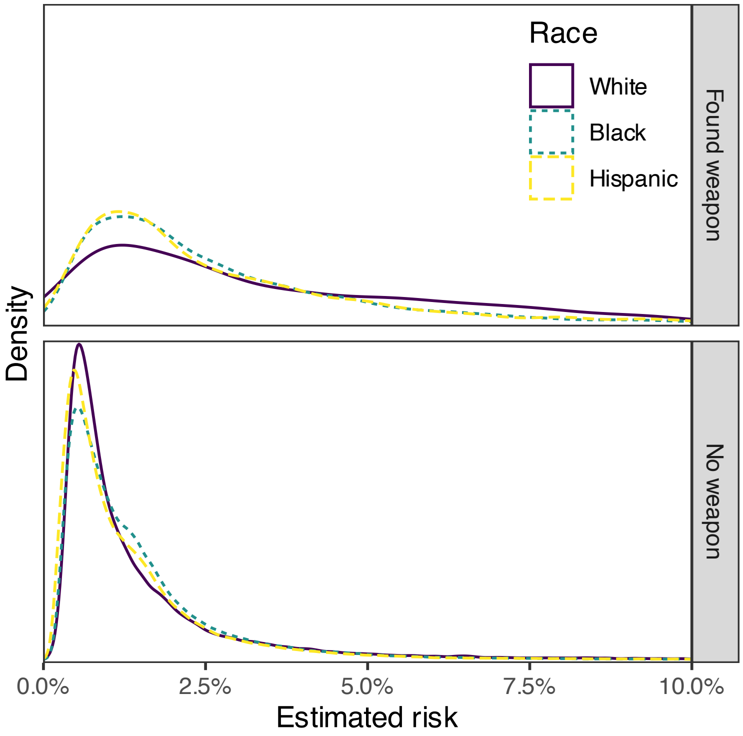

Fig. S16. *Distributions of the estimated* ex ante *probability of carrying a weapon—estimated without race as a covariate—for stopped pedestrians, on a log scale among frisked individuals, faceted by whether or not a weapon was found.*

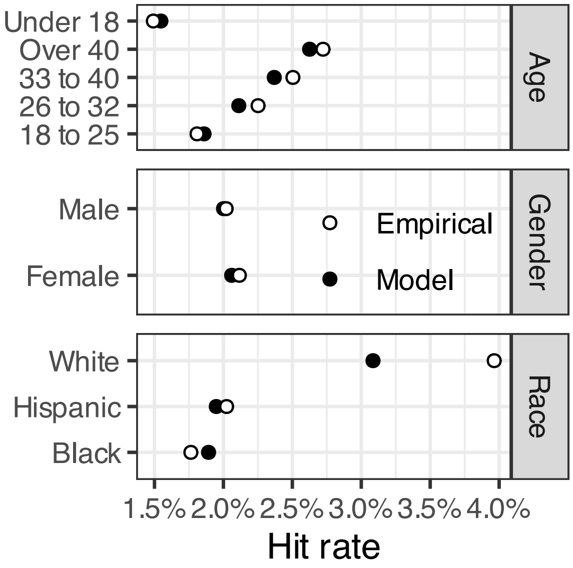

(a) Demographic groups

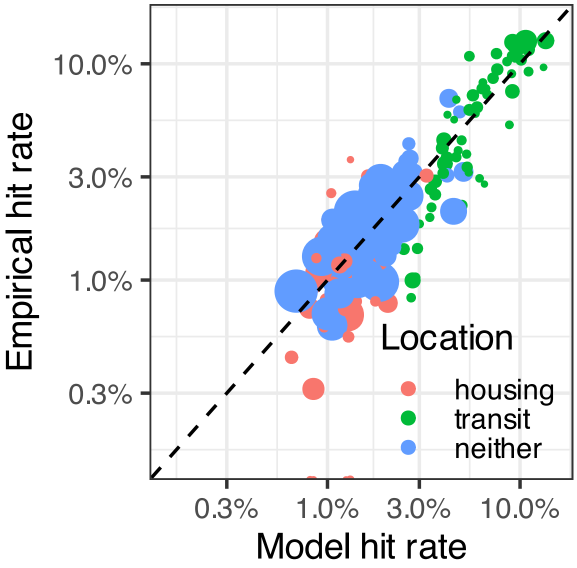

(b) Locations

Fig. S17. *Comparison of model-predicted versus empirical weapon recovery rate ("hit rate"), where the risk model does not include race as a covariate. Figure S17a shows that the model-predicted hit rates are close to their empirical counterparts, conditional on values of age, race, and gender. In Figure S17b, stops are binned by precinct and stop location. Points are plotted for each bin with more than 100 stops, sized by the number of stops, with colors representing the stop location type: transit, housing, or other. The plotted points are near the diagonal, suggesting that the outcome model is well-calibrated and predicts well over the full range of hit rates and frisk rates, respectively. The model itself achieves an AUC of 81% on the second half of the data.*

References

1. Washington v. Davis, 426 U.S. 229 (1976).
2. SFFA v. Harvard, Students for Fair Admissions, Inc., Petitioner, v. President and Fellows of Harvard College. Students for Fair Admissions, Inc., Petitioner, v. University of North Carolina, et al. (2023) https://www.supremecourt.gov/opinions/22pdf/20-1199_l6gn.pdf.
3. TJ VanderWeele, WR Robinson, Confounding and mediating variables. *Epidemiology* **25**, 473–484 (2014).
4. DJ Greiner, DB Rubin, Causal effects of perceived immutable characteristics. *Rev. Econ. Stat.* **93**, 775–785 (2011).
5. J Fagan, Report of Jeffrey Fagan in the case of Floyd v. the City of New York (2010) Available at https://www.law.columbia.edu/sites/default/files/microsites/policing-litigation-conference/files/Fagan%20Report%20with%20Technical%20Appendices.pdf.
6. J Gaebler, et al., A causal framework for observational studies of discrimination. *Stat. Public Policy* **9**, 26–48 (2022).
7. J Grossman, J Nyarko, S Goel, Reconciling legal and empirical conceptions of disparate impact: An analysis of police stops across California. *J. Law & Empir. Analysis* **1**, 118–133 (2024).
8. D Arnold, W Dobbie, P Hull, Measuring racial discrimination in bail decisions. *Am. Econ. Rev.* **112**, 2992–3038 (2022).
9. D Arnold, W Dobbie, P Hull, Measuring racial discrimination in algorithms in *AEA Papers and Proceedings.* Vol. 111, pp. 49–54 (2021).
10. JA Bohren, P Hull, A Imas, Systemic discrimination: Theory and measurement. *The Q. J. Econ.* pp. 1743–1799 (2025).
11. A Coston, A Mishler, EH Kennedy, A Chouldechova, Counterfactual risk assessments, evaluation, and fairness in *Proceedings of the 2020 Conference on Fairness, Accountability, and Transparency.* pp. 582–593 (2020).
12. M Hardt, E Price, N Srebro, Equality of opportunity in supervised learning. *Adv. Neural Inf. Process. Syst.* **29** (2016).
13. I Ayres, Three tests for measuring unjustified disparate impacts in organ transplantation: The problem of "included variable" bias. *Perspectives Biol. Medicine* **48**, 68–S87 (2005).
14. SG Mayson, Bias in, bias out. *Yale Law J.* **128**, 2218 (2018).
15. H Nilforoshan, JD Gaebler, R Shroff, S Goel, Causal conceptions of fairness and their consequences in *International Conference on Machine Learning.* (PMLR), pp. 16848–16887 (2022).
16. S Corbett-Davies, JD Gaebler, H Nilforoshan, R Shroff, S Goel, The measure and mismeasure of fairness. *J. Mach. Learn. Res.* **24**, 1–117 (2023).
17. PR Rosenbaum, DB Rubin, Assessing sensitivity to an unobserved binary covariate in an observational study with binary outcome. *J. Royal Stat. Soc. Ser. B (Methodological)* **45**, 212–218 (1983).
18. J Jung, C Concannon, R Shroff, S Goel, DG Goldstein, Simple rules to guide expert classifications. *J. Royal Stat. Soc. Ser. A (Statistics Soc.).* **183**, 771–800 (2020).
19. Q Zhao, DS Small, BB Bhattacharya, Sensitivity analysis for inverse probability weighting estimators via the percentile bootstrap. *J. Royal Stat. Soc. Ser. B: Stat. Methodol.* **81**, 735–761 (2019).
20. PR Rosenbaum, *Observational studies.* (Springer), (2002).
21. SP Boyd, L Vandenberghe, *Convex optimization.* (Cambridge University Press), (2004).
22. W Karush, Minima of functions of several variables with inequalities as side constraints. *M. Sc. Diss. Dept. Math. Univ. Chic.* (1939).
23. HW Kuhn, AW Tucker, Nonlinear programming in *Proceedings of the Second Berkeley Symposium on Mathematical Statistics and Probability, 1950.* (University of California Press, Berkeley-Los Angeles, Calif.), pp. 481–492 (1951).
24. S Sahni, Computationally related problems. *SIAM J. on computing* **3**, 262–279 (1974).
25. Y Zhang, Q Zhao, Bounds and semiparametric inference in l^∞- and l^2-sensitivity analysis for observational studies. *arXiv preprint arXiv:2211.04697* (2022).
26. M Huang, SD Pimentel, Variance-based sensitivity analysis for weighting estimators results in more informative bounds. *Biometrika* **112**, asae040 (2024).
27. C Cinelli, C Hazlett, Making sense of sensitivity: Extending omitted variable bias. *J. Royal Stat. Soc. Ser. B-Statistical Methodol.* **82**, 39–67 (2020).
28. J Dorn, K Guo, Sharp sensitivity analysis for inverse propensity weighting via quantile balancing. *J. Am. Stat. Assoc.* pp. 1–13 (2022).
29. S Goel, JM Rao, R Shroff, Precinct or prejudice? Understanding racial disparities in New York City's stop-and-frisk policy. *Annals Appl. Stat.* **10** (2016).
